# Structural MRI across lifespan reveals differential thalamic trajectories in Down syndrome

**DOI:** 10.1002/alz.71671

**Published:** 2026-07-14

**Authors:** Muhammad Shaikh, Paul Fletcher, Shahid Zaman, The Alzheimer's Biomarker Consortium on Down Syndrome, Stephanie Brown

**Affiliations:** ^1^ Department of Psychiatry University of Cambridge Cambridge Cambridgeshire UK; ^2^ Cambridgeshire and Peterborough NHS Foundation Trust Fulbourn Hospital Cambridge Cambridgeshire UK

**Keywords:** Alzheimer's disease, Down syndrome, neurodegeneration, Papez Network, thalamus

## Abstract

**INTRODUCTION:**

Up to 90% of Down syndrome (DS) patients develop Alzheimer's disease (AD). Sleep disturbance, affecting over 75% of DS patients, is implicated in AD pathogenesis. The thalamus, central to sleep and arousal, shows early vulnerability in DS‐related AD.

**METHODS:**

Structural 3T MRI scans from 253 DS participants (119 males, mean age 43.0 ± 9.4 years) and 36 controls (30 males, mean age 43.1 ± 12.2 years) from the Alzheimer's Biomarker Consortium‐Down Syndrome (ABC‐DS) study were analyzed, alongside neurodegenerative plasma biomarker assays (phosphorylated tau [pTau]181, pTau217, neurofilament light chain [NfL], amyloid beta [Aβ]40, Aβ42).

**RESULTS:**

In DS, intracranial volume‐adjusted thalamic volume declined with age (*t* = −2.589, *p* = 0.00987), approximating 2.5% loss per decade. This was linear, gray matter involution‐independent, and heterogeneous across nuclei, correlating negatively with pTau and NfL. Controls exhibited no significant volumetric changes.

**DISCUSSION:**

Anteromedial and posterior thalamic shrinkage in DS AD mirrors sporadic AD. Associations with neurodegenerative biomarkers support thalamic atrophy as a sensitive marker of DS‐related AD progression.

## Highlights

- Thalamic volume declines linearly with age in Down syndrome (DS)
- Age‐related thalamic atrophy in DS approximates 2.5% loss per decade
- Thalamic subnuclei show heterogeneous vulnerability in DS‐related Alzheimer's disease (AD)
- Thalamic size correlates negatively with plasma pTau and neurofilament light
- Thalamic degeneration in DS mirrors patterns seen in sporadic AD

## 1 BACKGROUND

Down syndrome (DS) is a genetic syndrome resulting from complete or partial trisomy of chromosome 21. It is phenotypically characterized by multiple co‐morbidities, including congenital heart defects and intellectual disability. Advances in the management of these co‐morbidities have significantly increased life expectancy. This has simultaneously revealed near‐universal Alzheimer's disease (AD) neuropathology in middle age, in the form of amyloid plaques and neurofibrillary changes. This neuropathology mediates an exceptional risk of later developing Alzheimer's dementia.^1^

In DS, the mean age of dementia diagnosis is 55 years, with a prevalence of approximately 90% after the age of 65 years.^1^ This penetrance is underpinned by triplication of the amyloid precursor protein (*APP*) gene on chromosome 21, upon which AD is dose‐dependent. The *APP* gene is epistatic to additional genes on chromosome 21 that may further modulate the disease course. Once manifest, AD progresses similarly in DS as in the general population. Across both populations, the apolipoprotein E (APOE) ε2 and ε4 alleles, respectively, decrease and increase amyloid beta (Aβ) deposition and risk of Alzheimer's dementia. Likewise, variants of the *PICALM* and *SORL1* genes influence age of dementia onset in both DS and late‐onset Alzheimer's dementia, suggesting a shared genetic architecture. The overall neuroanatomical distribution and biochemical composition of plaques (predominantly composed of Aβ) and neurofibrillary changes (tau protein tangles and neuropil threads) is similar across DS and the general population ^2^. The correspondence of the phenotypes across both populations validates and motivates the study of DS to better characterize AD more generally, including in its nascence.

Relevant to this research program, thalamic abnormalities may contribute to mnemonic impairments in Alzheimer's dementia, in addition to medial temporal lobe structures. Braak and Braak's seminal paper, which describes the chronology of neuropathological change in AD, found that while amyloid deposition was variable, the pattern of neurofibrillary changes was more consistent across individuals, beginning in the entorhinal region (stages I–II). Notably, the anterodorsal thalamic nuclei exhibited neurofibrillary changes contemporaneously with the hippocampus (stages III–IV), prior to many other regions.^3^ Later work confirms amyloidosis in almost all thalamic nuclei, and neurofibrillary changes in the limbic thalamus (anteroventral, laterodorsal, central medial, paratenial, and reticular nuclei).^4^, ^5^

This pattern is salient as it disproportionately maps onto thalamic sites reciprocally connected with the hippocampus. This substantiates the proposition that episodic memory deficits (one of the earliest cognitive deficits) in AD dementia reflect wider neurodegeneration in what is referred to as the Papez network, an extended mnemonic system which critically involves the limbic thalamus.^6^, ^7^, ^8^, ^9^ This extends the classical view, which primarily implicates medial temporal degeneration and may therefore underestimate the extent to which thalamic dysfunction explains cognitive impairment.

In DS, medial temporal structures, which are smaller even after adjustment for brain volume,^10^, ^11^ are well characterized longitudinally, degenerating with a strong positive correlation with fluid biomarkers of AD.^12^, ^13^ However, longitudinal characterization of the thalamus is less understood. A striking loss of volume (approximating a 70% loss of neurons) in the anterior thalamic nuclei in aged DS.^14^ suggests that other thalamic nuclei may also follow a similar degenerative pattern to the medial temporal lobe. In line with a Papez network specification of the network degeneration hypothesis,^15^ APOE ε2 carriers exhibit greater resting‐state functional connectivity involving the thalamus,^16^ whereas APOE ε4 carriers exhibit reduced hippocampal functional connectivity with the thalamus.^17^

Another line of evidence motivates longitudinal thalamic characterization. An emerging correlational evidence base increasingly implicates disordered sleep as a modifiable risk factor for AD.^18^ Sleep disorders are prevalent in the majority of DS cases,^19^ potentially accounting for a fraction of AD risk, should the sleep‐AD association prove causal. The thalamus is implicated in the regulation of consciousness and arousal,^20^, ^21^, ^22^, ^23^ constituting a potential intervention target in insomnia.^24^ Hence, we sought to longitudinally characterize the thalamus in two complementary cohorts: Sleep‐DS (a younger cohort with ultra‐high resolution neuroimaging), and Alzheimer's Biomarker Consortium‐Down Syndrome (ABC‐DS; an older and much larger cohort, conferring greater statistical power) to uncover patterns of early brain vulnerability.

**RESEARCH IN CONTEXT**

1. **Systematic review**: We noted from a literature survey (PubMed, recent conference abstracts) that > 75% of individuals with Down syndrome (DS) experience sleep abnormalities that may accelerate amyloid and tau accumulation. Neuroimaging studies in sporadic Alzheimer's disease (AD) highlight early thalamic involvement. The thalamus is implicated in the regulation of consciousness and arousal. However, thalamic subnuclei have not been systematically studied in DS‐related AD.
2. **Interpretation**: We show that thalamic atrophy is detectable in mid‐adult DS, follows a heterogeneous subnuclear pattern, and mirrors vulnerability patterns in sporadic AD. Strong associations between subnuclear volume loss and plasma AD biomarkers suggest AD‐related neurodegeneration. These findings position thalamic subnuclei as sensitive structural biomarkers of AD progression in DS.
3. **Future directions**: Longitudinal multimodal studies combining high‐resolution MRI, temporal macrostructural segmentation, plasma biomarkers, and sleep measures are needed to clarify temporal relationships. Sleep‐focused intervention trials should test whether improving sleep stabilizes thalamic decline or slows biomarker progression.

## 2 METHODS

### 2.1 Participants

Data from two cohorts were analyzed in this study: Sleep‐DS, and ABC‐DS^25^. Sleep‐DS is a single‐site cross‐sectional study based in the University of Cambridge Department of Psychiatry, led by the authors. A variety of recruitment strategies were used to recruit a cohort without a clinical AD dementia diagnosis, where approximately half the participants had DS and half did not. ABC‐DS is an ongoing multisite longitudinal study, from which we used data from the first session for cross‐sectional analysis.

For Sleep‐DS, the inclusion criteria for participants with DS were: trisomy 21 (as confirmed by medical record review), between 25 and 50 years of age, reliable carer capable of aiding the participant where necessary, agreement of carer and clinician/researcher that the subject is able to cooperate with the protocol tasks, provision of legally valid consent or assent, and adequate visual and auditory acuity to complete neuropsychological testing.

The inclusion criteria for control participants were: age between 25 and 50 years, and provision of informed consent. Exclusion criteria for all participants were: lack of participant consent, willingness, and ability to comply with the study protocol, significant disease (including clinical diagnoses of mild cognitive impairment or dementia), known use of psychotropic drugs, and unstable medical conditions. Control participants had the supplementary exclusion criteria of a diagnosed or known sleep condition.

The ABC‐DS study recruitment strategy has previously been published and is hence not reiterated here^26^. Cohort demographics for both cohorts are summarized in Table 1.

**TABLE 1:** Per‐cohort demographic statistics.

Parameter	Control	DS	Total	Test statistic	*p*‐Value
**Sleep‐DS**					
*N*	18	22	40	–	–
M (F)	9 (9)	10 (12)	19 (21)	X^2 ^= 0	1
Age (SD)	37.2 (8.1)	37.4 (6.3)	37.3 (7.0)	*t* = ‐0.10	0.92
**ABC‐DS**					
*N*	36	253	289	–	–
M (F)	30 (6)	119 (134)	149 (140)	X^2 ^= 15.2	<0.0001
Age (SD)	43.1 (12.2)	43.0 (9.4)	43.0 (9.8)	*t* = 0.05	0.96

Abbreviations: ABC‐DS, Alzheimer's Biomarker Consortium‐Down Syndrome; DS, Down syndrome; SD, standard deviation.

Genetic testing was performed for participants whose karyotype had not been previously determined. Informed assent or consent was obtained from all participants or their legal representatives prior to inclusion in the study. A standardized study protocol was implemented across all sites, with Research Ethics Committee (REC) approval obtained in accordance with the ethical principles of the 1964 Declaration of Helsinki and its later revisions. The REC reference for ABC‐DS is 21/WA/0365; the REC reference for Sleep‐DS is 22/YH/0123.

The preparation of this manuscript was made possible from data obtained by the ABC‐DS, a longitudinal study of AD biomarkers in adults with DS supported by grants from the National Institute on Aging (NIA) and the Eunice Kennedy Shriver National Institute of Child Health and Human Development (NICHD). The Principal Investigators of the ABC‐DS study are Benjamin Handen, PhD, and William Klunk, MD, PhD (University of Pittsburgh), Bradley Christian, PhD (University of Wisconsin‐Madison), Nicole Schupf, PhD, Dr PH (Columbia University), and Ira Lott, MD, and Wayne Silverman, PhD (University of California‐Irvine). The ABC‐DS study is a collaboration of field sites at Pittsburgh, Madison, Phoenix (Banner Alzheimer's Institute; Marwan Sabbagh, MD), the University of Cambridge (Shahid Zaman, MD), Washington University (Beau Ances, MD, PhD, and John Constantino, MD), Columbia University, Harvard University (Florence Lai, MD, and H Diana Rosas, MD) and University of California‐Irvine.

### 2.2 Cognitive testing

The Cued Recall Task, which evaluates episodic memory, was completed by DS participants only. The present test has been modified from the version developed for the typical population^27^ and has been evidenced to be a reliable marker of cognitive decline in DS.^28^ The free recall component of this test is known to reliably associate with blood biomarkers in pre‐clinical AD.^28^

Three cards with four pictures per card were shown to participants, one card at a time. During the training phase, participants were given a unique category cue and were asked to point to and name the relevant picture. After naming each picture on the card, the card was hidden from sight, and participants were asked to recall the pictures. This was repeated for all three cards. During the test phase, participants were asked to recall all the pictures (Free Recall). If they were not able to name all pictures spontaneously, they were given cues for the remaining items (Cued Recall).

### 2.3 Neuroimaging

Sleep‐DS participants were imaged at 7T field strength using a Siemens 7T Terra MRI scanner. For these participants, a T1‐weighted image was created by optimizing an MP2RAGE sequence (self‐biased corrected sequence for improved saturation and T1‐mapping at high field) to obtain two UNI images (INV1 & INV2),^29^ which were later combined and denoised using LayNii's ‘DNoise’ tool^30^ to improve signal‐to‐noise ratio. Acquisition parameters were as follows: repetition time (TR) = 4300 ms, echo time (TE) = 1.99 ms, inversion time (TI) = 840/2370 ms, flip angle (FA) = 5/6, bandwidth (BW) = 250 Hz, integrated parallel acquisition technique (iPAT) = 3, acquisition time (TA) = 8'50‘’, voxel size = 0.75 mm isotropic resolution.

ABC‐DS participants were imaged at 3T field strength as previously reported^26^ to obtain high‐resolution T1‐magnetization‐prepared rapid gradient echo (MPRAGE) scans. These were subsequently harmonized using the ComBat method^31^ to control for multisite scanner effects.

For all participants, cortical and sub‐cortical reconstruction and volumetric segmentation were performed with the Freesurfer image analysis suite (version 7.4.1), which is documented and freely available for download online (http://surfer.nmr.mgh.harvard.edu/). The technical details of these procedures are described in prior publications^32^, ^33^. The thalamus was parcellated into 25 different nuclei using a probabilistic atlas created with histological data and ex‐vivo brain MRI scans, a technique directly operationalized in Freesurfer and shown to be reliable between subjects and robust to imaging inhomogeneities.^33^, ^34^ All parcellations were visually inspected to confirm anatomical accuracy.

### 2.4 Plasma samples and analysis

Plasma concentrations of Aβ42, Aβ40, phosphorylated tau (pTau) 217, pTau181, neurofilament light chain (NfL), and C‐reactive protein (CRP) were quantified in accordance with established protocols.^35^ Samples were first analyzed at the University of North Texas, where Aβ42, Aβ40, and NfL levels were measured using single‐molecule array (Simoa) assays (Quanterix). Subsequently, samples from the same cohort were sent to Lund University, where pTau181 and pTau217 concentrations were quantified using an immunoassay on the Meso Scale Discovery platform, according to protocols developed by Lilly Research Laboratories.^36^, ^37^ APOE ε4 genotype was identified using a KASP genotyping assay (LGC Genomics, Beverly, MA, USA), and individuals carrying one or more ε4 alleles were classified as ε4 carriers. The full protocol has been extensively detailed.^26^

### 2.5 Statistical analysis

Statistical analyses were conducted using R version 4.5.1, with the ggplot2 package (version 4.0.0) used for data visualization. Chi‐squared tests were used to examine statistical differences in baseline characteristics within cohorts. Group differences in estimated total intracranial volume (eTIV) ‐normalized thalamic and subnuclear volumes were assessed using two‐sample Welch t‐tests. This approach does not assume equal variances between groups and applies the Welch‐Satterthwaite approximation, resulting in fractional degrees of freedom.

Volumes of interest were normalized for intracranial volume by simple division of the former by the latter. A series of generalized linear regressions with Gaussian link functions were used to model volumetric differences with age. For our age‐related modelling, we used the equation:

$$\begin{array}{ccc} & & Thalamic\;volume\;∼\;age\;\times group\;\times hemisphere+sex\\ & & +total\;gray\;matter\;volume \end{array}$$

To examine the interaction of APOE4 genotype with thalamic age trajectory, we used:

Thalamicvolume∼age×hemisphere×APOE4copynumber+sex

Finally, to ascertain biomarker relationships, we used:

Thalamicnucleusvolume∼biomarker

For our biomarker analyses, we used the sensemakr 0.1.6 package for R^38^ to compute partial R^2^ values for each biomarker by comparing the full (with biomarker) and reduced (without biomarker) models using model deviances. Cohen's f^2^ was derived therefrom. Where multiple comparisons were made (including all t‐tests and generalized linear model [GLM] results), *p*‐values were corrected using the Benjamini‐Hochberg procedure for false discovery rate (FDR) correction.

### 2.6 Participant demographics

Results from analyses of two cohorts are presented in this study: Sleep‐DS, and ABC‐DS. In both cohorts, participants with a genetic DS diagnosis and demographically matched control participants underwent structural MRI scans, a visual memory test, and gave blood samples which were assayed for biomarkers of neurodegeneration. Cohort demographics are summarized in Table 1.

## 3 RESULTS

### 3.1 Thalamic volumetrics

#### 3.1.1 Absolute thalamic volume decrements in DS are abolished by normalizing for intracranial volume

**FIGURE 1:**
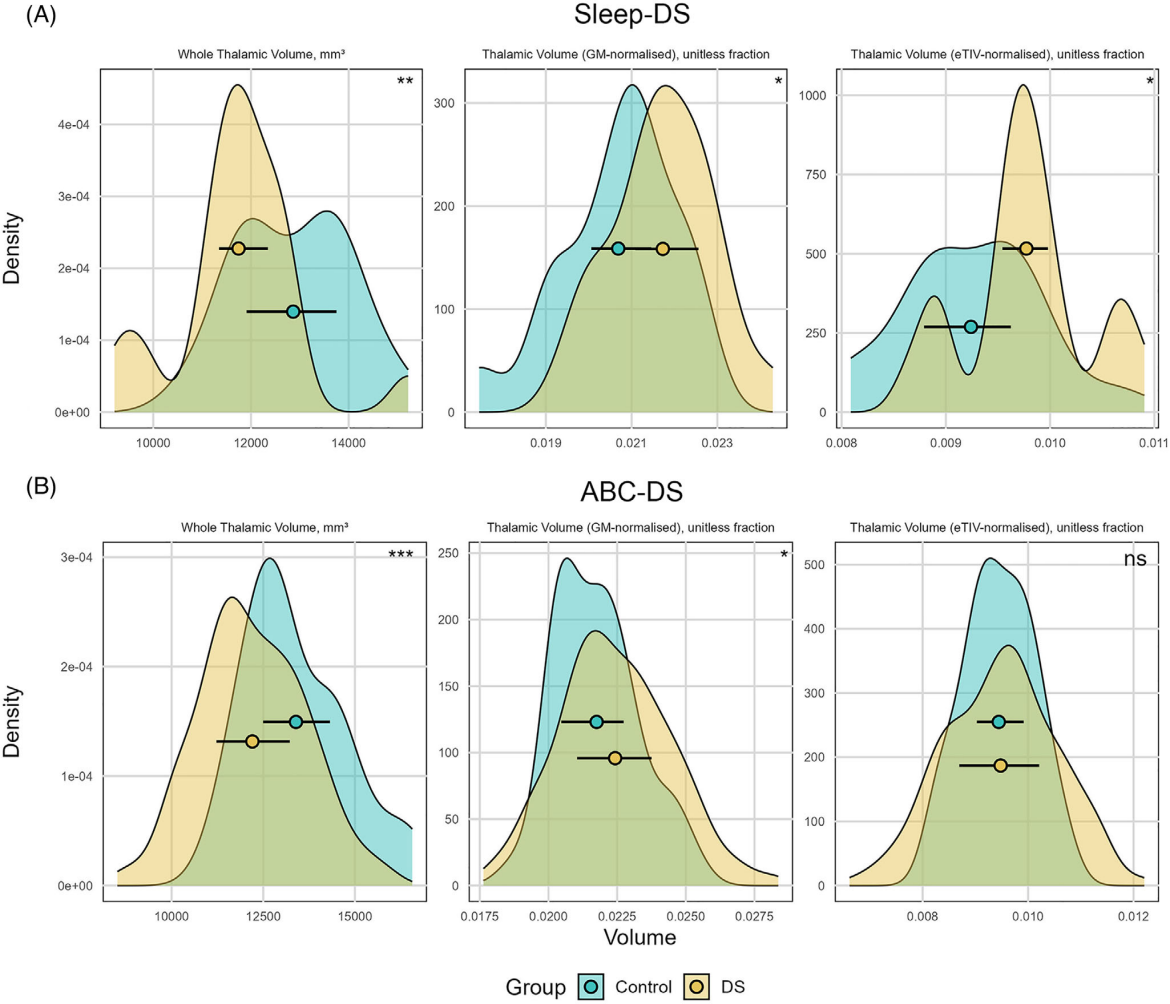
Absolute thalamic volume decrements in Down syndrome are largely abolished by normalizing for intracranial volume. Density plots for volumetrics of interest across groups in Sleep‐DS (A) and ABC‐DS (B) with overlying points and brackets indicating the distribution means and interquartile ranges respectively. Results of FDR‐corrected t‐tests are summarized in the top corners of the facets. Note the differing volume units provided in the facet titles. For this table and hereafter, ns = *p* ≥ 0.05, *  =  *p*  <  0.05, **  =  *p*  <  0.01, and ***  =  *p*  <  0.001. ABC‐DS, Alzheimer's Biomarker Consortium‐Down Syndrome; DS, Down syndrome; FDR, false discovery rate.

Absolute thalamic volume was significantly lower in DS compared to controls across cohorts (Figure 1, Table S1). In the Sleep‐DS cohort, DS participants had a mean thalamic volume of 11,745 mm^3^ compared to a mean control volume of 12,865 mm^3^. This volume decrement was significant, *p*_FDR_ = 0.0092. In the ABC‐DS cohort, DS participants had a mean thalamic volume of 12,203 mm^3^ compared to a mean control volume of 13,392 mm^3^. This volume decrement was also significant, *p*_FDR_ = 2.92×10^−^^5^.

Intracranial volume was significantly reduced in DS across cohorts (Figure 1). In the Sleep‐DS cohort, DS participants had a mean intracranial volume of 1,205,231 mm^3^ compared to a mean control volume of 1,396,272 mm^3^. This volume decrement was significant, *p*_FDR_ = 0.00042. In the ABC‐DS cohort, DS participants had a mean intracranial volume of 1,298,120 mm^3^ compared to a mean control volume of 1,420,316 mm^3^. This volume decrement was likewise significant, *p*_FDR_ = 2.92×10^−^^5^.

However, after normalization for intracranial volume, thalamic volume was marginally lower, or equal, in DS compared to controls (Figure 1). In the Sleep‐DS cohort, DS participants had a mean thalamus‐to‐intracranial volume ratio of 0.00980 compared to a mean control ratio of 0.00920. This decrease in ratio was significant, *p*_FDR_ = 0.015. However, in the ABC‐DS cohort, there was no significant ratio difference. These results are summarized in Figure 1. Refer to Table S1 for detailed statistics.

#### 3.1.2 Relative volume increments in specific nuclei robustly characterize Down syndrome

**FIGURE 2:**
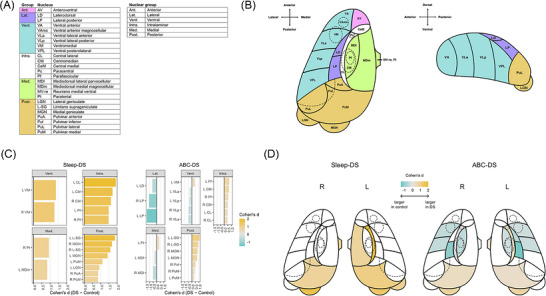
Relative volume increments in specific nuclei robustly characterize DS. (A) Thalamic nuclei groups and abbreviations, with (B) corresponding anatomical location and group (indicated by shading) shown on a schematized diagram of the right thalamus, adapted from ref.^9^ Dashed lines indicate positions of deeper nuclei. (C) Bar plots showing magnitudes of differences in means of eTIV‐normalized thalamic nuclei volumes for Sleep‐DS (left) and ABC‐DS (right). Only significant comparisons after FDR‐corrected t‐tests are shown. Positive values (yellow) indicate nuclei relatively larger in DS; negative values (teal) indicate nuclei relatively larger in controls. (D) Schematized anatomical visualization (adapted from ref.^9^) of significant mean differences in (C), sharing the same shading convention. N.b. some significantly different nuclei lie deep in the thalamus and hence are not shown on this diagram. ABC‐DS, Alzheimer's Biomarker Consortium‐Down Syndrome; DS, Down syndrome; FDR, false discovery rate; eTIV, estimated total intracranial volume.

Following normalization for intracranial volume, a broad set of segmented thalamic nuclei exhibited volumetric differences in DS (Figure 2; values expressed as a unitless fraction of intracranial volume; FDR‐corrected Welch t‐tests). In the Sleep‐DS cohort, significant differences were observed across ventral, intralaminar, medial, and posterior nuclear groups. In the ventral group, bilateral ventromedial nuclei showed larger relative volumes in DS (right *p*_FDR_ = 0.0015, left *p*_FDR_ = 0.0019).

Within the intralaminar nuclei, robust DS‐related bilateral volume increases were seen in the centromedian (right *p*_FDR_ = 7.0×10^−^^5^, left *p*_FDR_ = 4.8×10^−^^5^) and parafascicular nuclei (right *p*_FDR_ = 1.7×10^−^^4^, left *p*_FDR_ = 1.7×10^−^^4^), and unilaterally in the left central lateral nucleus (*p*_FDR_ = 1.4×10^−^^5^).

Medial nuclei showed limited involvement, with incremental volume increases in DS seen unilaterally in the right paratenial (*p*_FDR_ = 0.018) and the left mediodorsal medial magnocellular nuclei (*p*_FDR_ = 0.037).

Posterior thalamic nuclei also showed extensive DS‐related volumetric differences. These included volume increases in the bilateral limitans suprageniculate nucleus (right *p*_FDR_ = 7.00×10^−^^5^, left *p*_FDR_ = 1.40×10^−^^5^), and the bilateral medial geniculate nucleus (right *p*_FDR_ = 4.80×10^−^^5^, left *p*_FDR_ = 2.40×10^−^^4^).

Additional posterior effects were observed within pulvinar subdivisions. The pulvinar anterior nucleus showed a significant right‐sided enlargement in DS (*p*_FDR_ = 0.039), while the pulvinar medial nucleus was significantly larger in DS bilaterally (right *p*_FDR_ = 0.042, left *p*_FDR_ = 0.0059).

Finally, a left‐lateralized enlargement was observed in the lateral geniculate nucleus (LGN; *p*_FDR_ = 0.022). Together, these findings indicate selective posterior thalamic enlargement in DS following intracranial volume normalization, particularly involving geniculate and pulvinar nuclei.

In the ABC‐DS cohort, structurally similar but more heterogenous differences were observed in DS (Figure 2; values expressed as a unitless fraction of intracranial volume; FDR‐corrected Welch t‐tests).

Ventral nuclei demonstrated significant bilateral differences in ventral lateral subdivisions, including DS‐related volume decreases in the ventral lateral anterior (right *p*_FDR_ = 0.0064, left *p*_FDR_ = 0.049) and ventral lateral posterior nuclei (right *p*_FDR_ = 0.0064, left p_FDR_ = 0.019). Unilaterally, the left ventromedial nucleus was enlarged in DS (*p*_FDR_ = 0.020).

Intralaminar nuclei were consistently enlarged in DS, including the bilateral centromedian (right *p*_FDR_ = 0.0021, left *p*_FDR_ = 8.64×10^−^^6^), parafascicular (right *p*_FDR_ = 5.13×10^−^^4^, left *p*FDR = 4.52×10^−^^6^), and central lateral nuclei (right *p*_FDR_ = 0.0042, left *p*_FDR_ = 0.0012).

Medial nuclei showed mixed effects in DS, with a bilateral mediodorsal lateral parvocellular nuclear volume decrement (right *p*_FDR_ = 0.0021, *p*_FDR_ = 0.030) and a unilateral left paratenial nuclear volume increment (*p*_FDR_ = 0.0042).

Lateral nuclei showed robust bilateral contractions in DS within the lateral posterior nucleus (right *p*_FDR_ = 2.91×10^−^^8^; left *p*_FDR_ = 1.72×10^−^^8^). A unilateral left laterodorsal contraction was also observed (*p*_FDR_ = 7.91×10^−^^4^).

Posterior nuclei were prominently affected, with large bilateral DS‐related enlargements in the limitans suprageniculate nuclei (right *p*_FDR_ = 2.10×10^−^^8^, left *p*_FDR_ = 1.72×10^−^^8^), medial geniculate nuclei (right *p*_FDR_ = 2.18×10^−^^6^; left *p*_FDR_ = 6.00×10^−^^4^), and pulvinar medial nuclei (right *p*_FDR_ = 0.0042, left *p*_FDR_ = 0.044). An additional posterior enlargement was observed in the right pulvinar inferior nucleus (*p*_FDR_ = 6.00×10^−^^4^).

Together, these results indicate widespread but anatomically structured subnuclear volumetric differences in DS, emphasizing prominent involvement of intralaminar and posterior thalamic systems across cohorts. Refer to Table S2 for detailed statistics. Raw nuclear volumes (mm^3^) are provided for reference in Table S3.

#### 3.1.3 Thalamic volume decreases linearly with age in DS, heterogeneously across nuclei

For both the Sleep‐DS and ABC‐DS cohorts, we fitted a Gaussian GLM predicting eTIV‑normalized thalamic volume as a function of age, with DS status, hemisphere, sex, and gray matter volume included as covariates (see the Methods section for further details). Model coefficients for all models (reference levels: group = control, hemisphere = left, sex = female) are reported in Table 2.

**TABLE 2:** Per‐cohort model parameters.

Model	Observations	Term	Estimate	Standard error	Statistic	*p*‐value	95% CI lower bound	95% CI upper bound	Significance
**Sleep‐DS**									
Pooled	80	(Intercept)	0.005902	0.000527	11.20295	2.874827e‐17	0.00487	0.006935	***
		groupDS	0.000452	0.000492	0.918118	0.36171	−0.00051	0.001417	ns
		groupDS:hemisphereR	−0.00027	0.000685	−0.39709	0.692511	−0.00161	0.001071	ns
		hemisphereR	−5.62E‐05	0.000449	−0.12515	0.900762	−0.00094	0.000824	ns
		sexM	−0.00019	7.07392479111534e‐05	−2.62659	0.010587	−0.00032	−4.72E‐05	*
		totalGray	−5.92E‐10	5.66085600134233e‐10	−1.04639	0.298983	−1.70E‐09	5.17163693611164e‐10	ns
		visit_age	−2.20E‐05	8.68717946589669e‐06	−2.53227	0.013579	−3.90E‐05	−4.97E‐06	*
		visit_age:groupDS	−6.01E‐06	1.29122815794006e‐05	−0.46541	0.64308	−3.13E‐05	1.92980460764442e‐05	ns
		visit_age:groupDS:hemisphereR	6.565281766525e‐06	1.80378837509112e‐05	0.363972	0.716976	−2.88E‐05	4.19188842756317e‐05	ns
		visit_age:hemisphereR	1.46198671724616e‐06	1.18148664842509e‐05	0.123741	0.901875	−2.17E‐05	2.46186995085277e‐05	ns
**ABC‐DS**									
Pooled	578	(Intercept)	0.005042	0.000492	10.24515	1.02198e‐22	0.004077	0.006006	***
		groupDS	0.001107	0.000402	2.755419	0.00605	0.00032	0.001894	**
		groupDS:hemisphereR	9.64845823406418e‐05	0.000566	0.170457	0.864712	−0.00101	0.001206	ns
		hemisphereR	−8.24E‐05	0.000511	−0.16109	0.872079	−0.00108	0.00092	ns
		sexM	−0.00022	6.04359244353997e‐05	−3.64636	0.00029	−0.00034	−0.0001	***
		totalGray	−3.57E‐10	5.03626946395486e‐10	−0.70975	0.478148	−1.34E‐09	6.29639149816576e‐10	ns
		visit_age	−2.16E‐06	8.12013307831315e‐06	−0.26588	0.790432	−1.81E‐05	1.37562265349306e‐05	ns
		visit_age:groupDS	−2.33E‐05	9.0082108192398e‐06	−2.58912	0.009869	−4.10E‐05	−5.67E‐06	**
		visit_age:groupDS:hemisphereR	−3.09E‐06	1.26954997120432e‐05	−0.24365	0.807587	−2.80E‐05	2.17894186231397e‐05	ns
		visit_age:hemisphereR	3.31926448113e‐06	1.14366490545867e‐05	0.290231	0.771746	−1.91E‐05	2.57346847319437e‐05	ns
Female only	298	(Intercept)	0.004988	0.000695	7.181579	5.86117e‐12	0.003627	0.00635	***
		groupDS	0.001232	0.000555	2.221927	0.027063	0.000145	0.002319	*
		groupDS:hemisphereR	0.000114	0.000784	0.145903	0.8841	−0.00142	0.001651	ns
		hemisphereR	4.33803464232628e‐06	0.000653	0.006644	0.994704	−0.00128	0.001284	ns
		totalGray	−6.28E‐10	8.34678625357024e‐10	−0.75179	0.45279	−2.26E‐09	1.00843769775537e‐09	ns
		visit_age	9.04698506845923e‐07	1.01006365325917e‐05	0.089568	0.928692	−1.89E‐05	2.07015823316552e‐05	ns
		visit_age:groupDS	−2.41E‐05	1.25082167477476e‐05	−1.92887	0.054726	−4.86E‐05	3.8891652642751e‐07	ns
		visit_age:groupDS:hemisphereR	−4.42E‐06	1.75463065457795e‐05	−0.25198	0.801232	−3.88E‐05	2.99687356363077e‐05	ns
		visit_age:hemisphereR	1.69148293774773e‐06	1.42738752749539e‐05	0.118502	0.905752	−2.63E‐05	2.96677643964741e‐05	ns
Male only	280	(Intercept)	0.004924	0.000854	5.76672	2.199069e‐08	0.00325	0.006598	***
		groupDS	0.000919	0.000715	1.284774	0.199968	−0.00048	0.002321	ns
		groupDS:hemisphereR	0.000283	0.000996	0.283761	0.77681	−0.00167	0.002234	ns
		hemisphereR	−0.00031	0.000957	−0.32373	0.746395	−0.00219	0.001566	ns
		totalGray	−1.90E‐10	6.03923874740964e‐10	−0.31432	0.75352	−1.37E‐09	9.93844093780847e‐10	ns
		visit_age	2.70372161302608e‐06	1.74669026013421e‐05	0.154791	0.877101	−3.15E‐05	3.69382216331262e‐05	ns
		visit_age:groupDS	−2.90E‐05	1.78553167051548e‐05	−1.62419	0.105497	−6.40E‐05	5.99526356478909e‐06	ns
		visit_age:groupDS:hemisphereR	−5.89E‐06	2.51375999213174e‐05	−0.23444	0.814822	−5.52E‐05	4.33755940935934e‐05	ns
		visit_age:hemisphereR	7.43626675650322e‐06	2.43912695068253e‐05	0.304874	0.760696	−4.04E‐05	5.52422765270899e‐05	ns

Abbreviations: CI, confidence interval; DS, Down syndrome.

The Sleep‐DS model showed significant main effects of age (estimate = −2.20×10^−^^5^, standard error [SE] = 8.687×10^−^^6^, *t* = −2.53, *p* = 0.0135) and sex (lower normalized thalamic volume in males, estimate = −1.900×10^−^^4^, SE = 7.074×10^−^^5^, *t* = −2.627, *p* = 0.0106). Interestingly, the main effect of group (DS vs control) was not significant, in contrast to the modest effect detected by our previously employed FDR‐corrected t‐tests, perhaps indicating a loss of statistical power with inclusion of more covariates. Critically, the age × group interaction estimate in the Sleep‐DS model did not reach significance. The fitted Sleep‐DS model had 79 degrees of freedom for the null model and 70 residual degrees of freedom, with a null deviance = 9.254×10^−^^6^, residual deviance = 5.386×10^−^^6^ (a reduction of 3.868×10^−^^6^, ≈41.8% improvement over the null), and Akaike Information Criterion (AIC) = −1072.

The ABC‐DS model showed a significant main effect of group (DS vs control), with DS associated with smaller eTIV‑normalized thalamic volume at the model reference point (estimate = 1.107×10^−^^3^, SE = 4.017×10^−^^4^, t = 2.755, *p* = 0.00605). Sex was also a significant covariate: males had lower normalized thalamic volume than females in the reference cell (estimate = −2.204×10^−^^4^, SE = 6.044×10^−^^5^, *t* = −3.646, *p* = 0.00029). The main effect of age in controls was negligible and not significant (age estimate = −2.159×10^−^^6^, SE = 8.120×10^−^^6^, *t* = −0.266, *p* = 0.79). Hemisphere (right vs. left) did not differ significantly in baseline volume (estimate = −8.240×10^−^^5^, SE = 5.115×10^−^^4^, *t* = −0.161, *p* = 0.872).

**FIGURE 3 alz71671-fig-0003:**
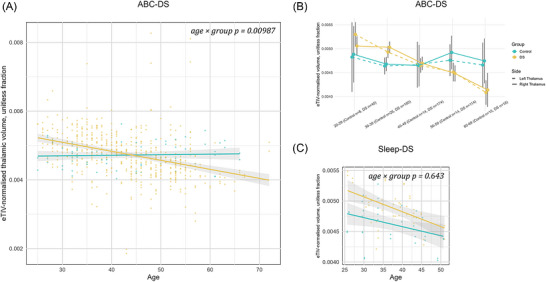
Normalized thalamic volume decreases linearly with age in DS. (A) GLM fits to eTIV‐normalized thalamic volume in the ABC‐DS cohort revealed a highly significant covariation with age bilaterally, with a DS group coefficient corresponding to an average decrement in thalamic volume of approximately 2.5% per decade. The control group coefficient was non‐significant, indicating no relationship of thalamic volume with age. Shading delineates the 95% confidence interval. (B) Plotting thalamic volume means in ABC‐DS within age bins of 10 years suggested that thalamic volume loss in DS occurs in a grossly linear fashion (brackets delineate the 95% confidence interval). (C) Summary of Sleep‐DS GLM findings, with a significant main effect of age across groups, but a non‐significant age × group interaction. ABC‐DS, Alzheimer's Biomarker Consortium‐Down Syndrome; DS, Down syndrome; eTIV, estimated total intracranial volume; GLM, generalized linear model.

Notably, the age × group interaction was significant and negative (estimate = −2.332×10^−^^5^, SE = 9.008×10^−^^6^, *t* = −2.589, *p* = 0.00987), indicating that age‑related decline in eTIV‑normalized thalamic volume is steeper in the DS group than in controls (Figure 3). The age × hemisphere and group × hemisphere two‑way interactions were not significant, and the three‑way interaction (age × group × hemisphere) was also non‑significant (estimate = −3.093×10^−^^6^, SE = 1.270×10^−^^5^, *t* = −0.244, *p* = 0.808), indicating that the age × group slope does not differ meaningfully between left and right thalami. The fitted ABC‐DS model had 577 degrees of freedom for the null model and 568 residual degrees of freedom, with a null deviance = 0.0002318, residual deviance = 0.0001927 (a reduction of 0.0000391, ≈16.9% improvement over the null), and AIC = −6958.

The significance of the age × group interaction despite the inclusion of gray matter volume in the GLM as a covariate indicates that this effect is specific to the thalamus and independent of generalized gray matter involution. Restating the gradient values more intuitively, this equates to a loss of volume of 2.33 × 10^−^^5^ fractions of intracranial volume per year, for the left and right thalami, respectively, that is, 0.00233% volume loss per year, or 0.0233% volume loss per decade, as a proportion of intracranial volume. As a proportion of thalamic volume (which equates to 0.00949 fractions of intracranial volume across both groups and cohorts, on average; see Table S1), this represents a rate of volume loss of ∼2.5% per decade.

Noting the significant sex imbalance between the DS and control groups in the ABC‐DS cohort, we ran sex‑stratified sensitivity analyses (male‑ and female‑only models) to assess whether the age × group interaction was driven by one sex (see Table 2). Neither stratified analysis produced a statistically significant age × group interaction. However, a formal test for sex moderation (age × group × sex) in the pooled model (above) did not provide evidence that the age × group slope differs by sex. Given that the pooled age × group interaction is statistically significant while the stratified tests are not, the most parsimonious interpretation is that the stratified null results reflect reduced sample size and loss of statistical power rather than a qualitatively different effect in males versus females. We therefore report the pooled, sex‑adjusted interaction as the primary finding, and note the limited precision of sex‑specific estimates as a study limitation.

**FIGURE 4 alz71671-fig-0004:**
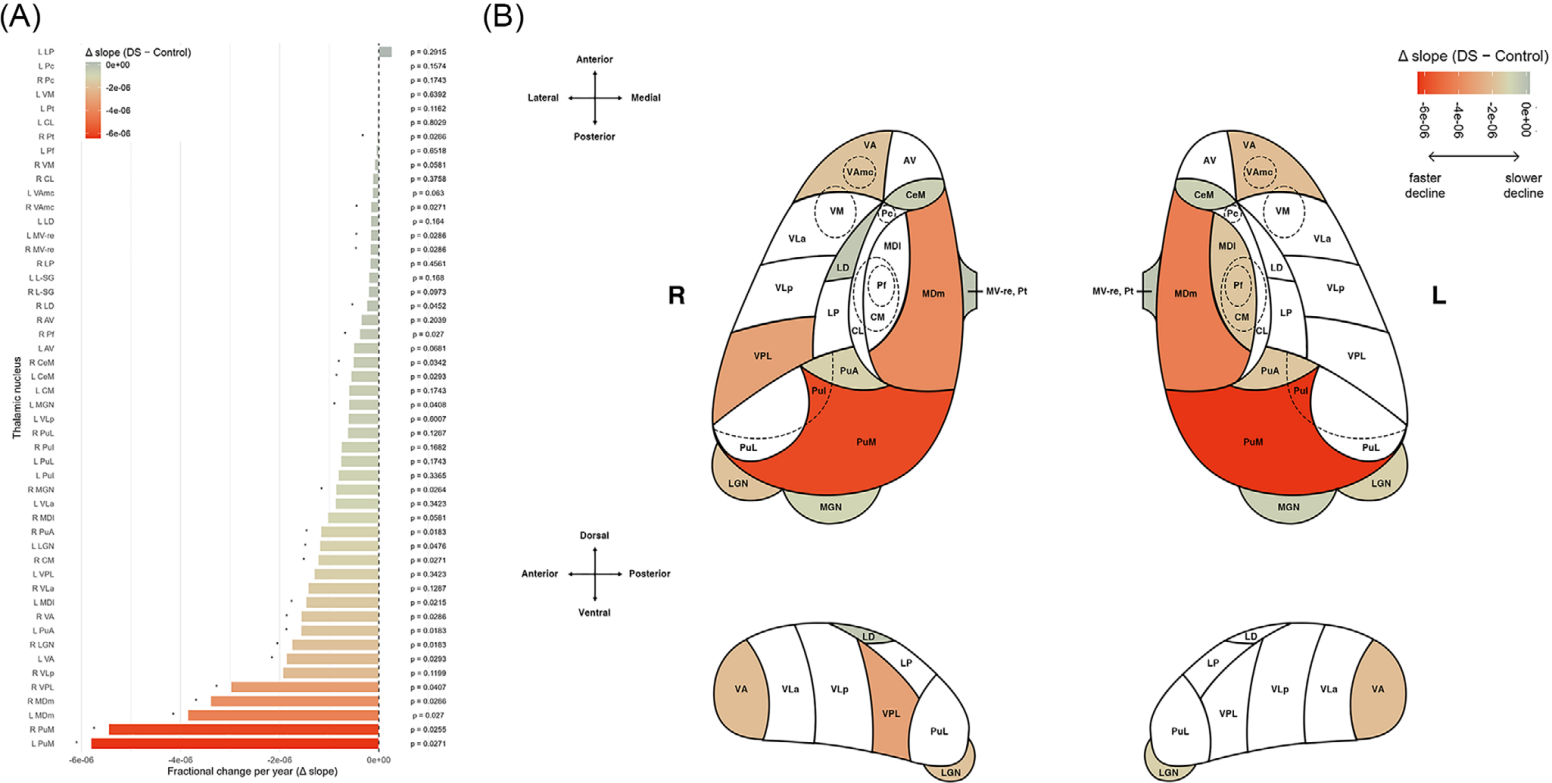
Age‐related thalamic volume decreases in Down syndrome are heterogeneous across nuclei. (A) GLM model coefficient (or slope) differences between the DS and control groups for eTIV‐normalized volumes of each thalamic nucleus. Higher values indicate larger volumetric decreases with age in DS relative to the control group. All *p*‐values and respective annotations are FDR‐corrected. (B) Schematized diagram (adapted from ref.^9^) showing slope differences for each thalamic nucleus, with color convention as in (A), where greater color saturation corresponds to a greater slope difference. Non‐significant nuclei are left unshaded. Dashed lines indicate positions of deeper nuclei. N.b. some significant nuclei lie deep in the thalamus and, hence, are not shown on this diagram. DS, Down syndrome; eTIV, estimated total intracranial volume; FDR, false discovery rate; GLM, generalized linear model.

We then repeated the model fitting process for each thalamic nucleus, setting the model outcome variable to eTIV‐normalized nuclear volume (Figure 4, summary Table S4). Sex and total gray matter volume were retained as covariates, whereas hemisphere was removed to maximize statistical power, given the previously ascertained lack of interaction. This revealed a grossly symmetrical age‐related decline, predominantly driven by the medial pulvinar and mediodorsal medial magnocellular nuclei.

### 3.2 Biomarker associations and cognitive tests

**FIGURE 5 alz71671-fig-0005:**
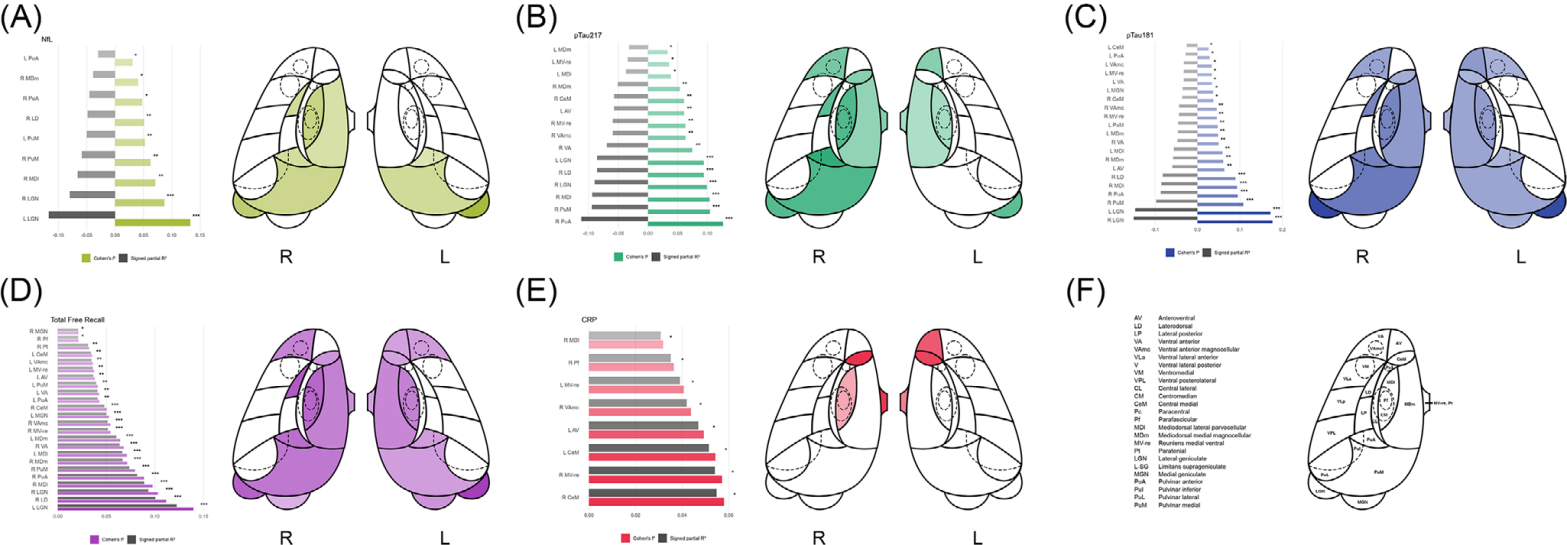
AD biomarker relationships with “shrinking” nuclei in DS. Bar plots (left, panels A–E) and schematized thalamic heatmaps (right, panels A–E, adapted from ref.^9^) showing associations between AD biomarkers and “shrinking” nuclei identified in the previous analysis, in terms of effect size (Cohen's f^2^, used for color convention, and signed partial R^2^); only significant covariations are shown after FDR‐correction. NfL (A), pTau217 (B), and pTau181 (C) all negatively covary with thalamic nuclear volumes of interest (hence negative partial R^2^ values). Total free recall (D) and C‐reactive protein (E) exhibit a positive covariance with the nuclei shown (hence positive partial R^2^ values). N.b. some significant nuclei lie deep in the thalamus and hence are not shown on these diagrams. (F) Anatomical diagram with nuclei names and abbreviations for reference. Refer to supplementary Figure 1 and supplementary Table S4 for further information. AD, Alzheimer's disease; DS, Down syndrome; FDR, false discovery rate; NfL, neurofilament light chain; pTau, phosphorylated Tau.

Restricting our analysis to the DS group only, we then explored whether the volumes of significantly “shrinking” nuclei covaried with key dementia‐related blood biomarkers, as well as a clinical biomarker (free recall). Key model parameters for these respective GLMs are presented in Table S5, and summarized in Figure 5. Our results within the Sleep‐DS cohort did not reach significance after FDR‐correction, and are hence not presented.

In the ABC‐DS cohort, across biomarkers, thalamic atrophy showed systematic and spatially organized associations with AD‐related plasma markers and cognition (Figure 5, Figure S1). pTau181 demonstrated the widest distribution of significant negative associations, involving nuclei across anterior, medial, ventral, and posterior groups (Figure 5c). Significant effects (typical *p*_FDR_ ≈ 0.001‐0.04) were present bilaterally in the lateral and medial geniculate nuclei, mediodorsal (lateral and medial) nuclei, pulvinar subdivisions (anterior, medial), and ventral anterior subdivisions.

pTau217 also exhibited robust negative associations, with significant nuclei overlapping those of pTau181 but with a slightly narrower distribution (Figure 5b). Significant *p*_FDR_ values (generally 0.002–0.04) were observed bilaterally in posterior (LGN) and mediodorsal (medial and lateral) nuclei, consistent with selective vulnerability of anteromedial and posterior thalamic circuits.

NfL was associated with a more restricted pattern of thalamic atrophy (Figure 5a), predominantly in posterior sensory nuclei and mediodorsal subdivisions, with significant *p*_FDR_ ≈ 0.001–0.045. Affected regions included the bilateral LGN and pulvinar subdivisions (anterior and medial nuclei).

CRP showed smaller and fewer significant associations (Figure 5e), with bilateral effects in central medial and medial ventral nuclei (*p*_FDR_ values mostly 0.03–0.049), suggesting only modest inflammatory contributions to thalamic degeneration.

In contrast, total free recall displayed widespread positive associations with thalamic volume, spanning nearly all nuclear groups (Figure 5d). Significant *p*_FDR_ values (typically < 0.01) were distributed across anteroventral, ventral, mediodorsal, pulvinar, and posterior geniculate nuclei, indicating that better memory performance strongly correlates with preserved thalamic integrity.

Finally, within ABC‐DS Down's syndrome cohort, we examined whether APOE ε4 genotype was associated with differential thalamic age trajectories by fitting a Gaussian GLM. No significant interaction was found between age‐related volume trajectories and APOE4 copy number. Refer to Figure S2 for further information, and to Table S6 for detailed model statistics.

## 4 DISCUSSION

We report absolute thalamic volume reductions in DS across two independent cohorts. We also novelly report that this deficit is predominantly a function of a smaller brain volume, as it is effectively entirely abolished by normalization for intracranial volume (and reversed when normalized by grey matter). Nucleus‐level analyses revealed specific and reproducible volumetric differences that persisted after stringent correction, with consistent involvement of medial, intralaminar, and posterior thalamic nuclei. Age‐related modelling demonstrated a steeper decline in thalamic volume in DS compared to controls, driven bilaterally by nuclei implicated in AD, particularly the mediodorsal and pulvinar subdivisions. DS‐related thalamic atrophy showed systematic associations with AD‐related plasma biomarkers and cognition. pTau181 exhibited the broadest pattern of negative associations, followed by pTau217 and NfL. In contrast, free recall performance correlated positively and widely with thalamic volume, underscoring the relevance of thalamic integrity to attention and memory.

To contextualize our finding of altered thalamic mesostructure in DS, we now review the literature relating to thalamic abnormalities in this syndrome. There is histological evidence of impairment in higher order thalamic nuclei in gestational weeks 17–22, at which point the mediodorsal, centromedian, and parafascicular nuclei exhibit reduced cell densities. These associate with reduced density of proliferating cells in the third ventricle, suggesting causative impairments in neurogenesis^39^. Previous neuroimaging evidence confirms absolute volumetric reductions in the thalamus in children^40^, ^41^ and young adults.^42^ In particular, the largest childhood decrements are seen bilaterally in the lateral[Fig alz71671-fig-0003], [Fig alz71671-fig-0004], [Fig alz71671-fig-0005] posterior (most significantly), laterodorsal, ventral anterior, ventral lateral anterior, and ventral lateral posterior nuclei, and unilaterally in the right mediodorsal lateral parvocellular, mediodorsal medial magnocellular, paracentral, and pulvinar anterior nuclei.^41^ In adulthood and old age, there are no neuroimaging descriptions prior to our study. Histological evidence in aged DS extends to the anterior nuclei as aforementioned,^14^ as well as the mediodorsal nuclei.^43^ Across age, all histological studies report a preferential loss of large (or projecting) neurons compared to small (or locally inhibitory) neurons, and of neurons relative to glia.

We report robust (i.e. across cohorts) intracranial volume‐adjusted increments bilaterally in the limitans suprageniculate (most significantly), medial geniculate, parafascicular, centromedian, and pulvinar medial nuclei, and unilaterally in the left central lateral nucleus. These changes may reflect relative preservation in the setting of global atrophy, compensation, or interactions between subsistent neurogenesis impairments and early neurodegeneration. Previous neuroimaging descriptions in early life do not adjust for intracranial volume, which may explain why reported volumetric differences skew toward larger nuclei in the setting of a known absolute reduction in thalamic volume.

The intralaminar centromedian and parafascicular nuclei are higher‐order nuclei, that is, they form reciprocal connections almost exclusively with association cortex. Notably, these nuclei are among those shown to be hypocellular in foetal development in Down syndrome.^39^ Given their involvement in executive control, this perhaps reflects an early neurodevelopmental divergence in the thalamocortical systems that support executive and attentional control. During normal foetal development, following rostrocaudal patterning of the neural tube, thalamocortical axons migrate into the cortical plate, where extrinsic thalamic signals interact with intrinsic genetic programmes to guide the formation of functionally specialized cortical territories. In infancy, the thalamus provides a scaffold for hierarchical cortical differentiation; once large‐scale networks are established, it shifts toward supporting functional segregation across distributed networks.^44^ Thus, early abnormalities within intralaminar nuclei may disrupt this developmental trajectory, contributing to lifelong alterations in executive and attentional function in DS.^45^

There is notable diversity in the type and severity of cognitive deficits in DS. Relative preservation of certain types of learning (simple conditioning, spatial memory) exist alongside highly specific impairments in spatial cognition and memory consolidation, suggesting differential impairment in specific medial temporal systems.^46^ Understanding the role of the thalamus in this network is complicated by its heterogeneity. Across the thalamus, integration of inputs with different origins, transmitters, and complexities is the rule. Unlike in modular networks where the input space is uniform across the structure (e.g. the striatum, cerebellum, or cortex), no canonical thalamic module exists.^47^ Therefore, rodent model research with high neuroanatomical and neurogenetic precision may be best positioned to characterize this cognitive phenotype, leveraging translatable behavioral tasks.^48^

Our imputed longitudinal thalamic effects are consistent with AD‐driven neurodegeneration. This aligns with our observed negative association of differentially affected nuclei with both pTau biomarkers. Plasma pTau217 is one of the earliest detectable signals in incipient AD, preceding even tau–positron emission tomography (PET) and amyloid‐PET signals and corresponding to intraparenchymal soluble tau hyperphosphorylation, an early pathophysiological process.^49^ Early reductions in Aβ42 in fluids are thought to reflect increased uptake of Aβ42 into amyloid plaques, and are seen together with increasing amyloid‐PET signals in the brain.^49^ These trends may explain why we see negative volume correlations with tau, but not amyloid, biomarkers.

The overarching radionuclide imaging story appears to be that amyloid deposits earlier^50^ and accumulates faster^51^, ^52^ in DS‐related AD. However, in contrast to post‐mortem findings,^53^ and other radiotracers,^52^, ^54^ studies utilizing [^11^C]Pittsburgh Compound‐B (PiB) find a striatal^55^ and thalamic,^56^ rather than cortical, predominant amyloid burden in DS. This may reflect morphological and dynamical (overproduction versus failed clearance) plaque differences, and is an intriguing avenue for further study.

We report a DS‐specific pattern of atrophy that is reproduced with few differences in multiple neuroimaging studies of AD in the general population, both longitudinal^57^ and cross‐sectional^58^, ^59^, ^60^, ^61^ (although with variable inclusion of the posterior nuclei, including the pulvinar and geniculate). Longitudinally, thalamic atrophy begins in the ventromedial region (corresponding to both mediodorsal nuclei), and then spreads to the anterior nuclei.^57^ This is independent of coincident internal medullary lamina atrophy, which segregates both nuclear groups.^59^ We also confirm involvement of both geniculate nuclei, which has twice been found in the ADNI dataset using our chosen segmentation method,^34^ as well as another.^61^

However, this neuroimaging pattern is not fully consistent with the neuropathological pattern,^34^ which implicates the anterior nuclei as the earliest sites of neurofibrillary change.^4^, ^62^ In late AD (Braak stages V–VI), the motor precerebellar (ventral lateral nucleus) and sensory nuclei (lateral and medial geniculate bodies, ventral posterior medial and lateral nuclei, and parvocellular segment of the ventral posterior medial nucleus; in our segmentation, this is part of the ventral posterolateral nucleus) are spared of tau cytoskeletal pathology.^5^ We and others^34^, ^61^ mostly recapitulate this sparing with respect to neurodegeneration, with the prominent exception of the two geniculate nuclei. Interestingly, these display some of the most significant negative associations with both pTau measures. This neuroimaging and neuropathological divergence merits further investigation.

The pulvinar and mediodorsal nuclei have been found in another analysis of sporadic AD to predominantly underlie a progressive decrease in thalamic volume with disease severity, and to most strongly correlate with neurocognitive scores among thalamic nuclei.^61^ The pulvinar is a higher‐order nucleus thought to support executive function by mediating directed attention.^63^ Together with the mediodorsal nucleus, another higher‐order hub, it contributes to context‐dependent cognition and behavioral flexibility.^64^, ^65^

Selective vulnerability of these nuclei in DS‐related AD may reflect early disruption of large‐scale associative networks that depend on thalamic coordination. Their strong functional coupling to prefrontal, temporal, and parietal association cortices positions them as central network nodes. However, this may predispose them to trans‐neuronally propagated circuit‐confined disease (as suggested by thalamic mirroring of tau pathoanatomy in the cortex^5^) leading to disproportionate cognitive impairments. The overlap between the nuclei affected in DS and in sporadic AD further suggests a shared systems‐level mechanism of thalamocortical breakdown, with early involvement of higher‐order thalamic hubs marking a convergent neurodegenerative pathway. This may align with early PiB findings in the thalamus.^56^

We did not replicate age‐related decline in normalized thalamic volume in our control cohort. This may be due to both a minimal effect size and a small cohort relative to the original study.^66^

We acknowledge several limitations to our study. First, a significant sex‐imbalance between experimental groups in the ABC‐DS cohort may confound our imputed longitudinal effect. However, the age × group × sex interaction in the pooled analysis, as well as the age × group interactions in our sex‐stratified sensitivity analyses (Table 2) were all non‐significant, suggesting that the latter sex‐stratified null results reflect reduced power rather than a true sex difference in thalamic trajectory.

Second, the Bayesian segmentation relies on predefined nuclear boundaries, particularly between the mediodorsal and pulvinar, to warp a probabilistic atlas. It may therefore fail to fully capture underlying anatomic variability or disease‐related boundary shifts. Third, this is a cross‐sectional study, and our analyses assume linear rates of atrophy, which may oversimplify true longitudinal trajectories.^13^ Finally, while our findings could partially reflect ventricular expansion associated with Alzheimer's pathology, this does not account for changes in non‐adjacent nuclei, including the geniculate and anterior nuclei.

The anteromedial and posterior pattern of thalamic atrophy observed in DS‐related AD mirrors that reported in AD within the general population. The linear decline beginning in midlife and covarying with AD blood biomarkers supports further validation of thalamic volume as a sensitive biomarker of neurodegenerative change. An increased AD classification accuracy when incorporating thalamic nuclei volumetrics^34^ motivates this further. Our findings suggest a wider‐scale, mesoscopically sensitive description of volumetric changes throughout the medial temporal lobe as an important future research avenue.

## AUTHOR CONTRIBUTIONS

**Muhammad Shaikh** (first author) handled the data, designed the methods, carried out the research, performed the analyses, helped manage the project, provided resources, wrote and maintained the software, checked the accuracy of the work, created the figures, wrote the first draft, and revised and edited the manuscript. **Paul Fletcher** provided resources, checked the accuracy of the work, contributed to reviewing and editing the manuscript, and offered supervision. **Shahid Zaman** worked on data collection, secured funding, carried out the research, contributed to the study design, helped manage the project, provided resources, checked the accuracy of the work, and contributed to reviewing and editing the manuscript. **Stephanie Brown** (senior author) led the overall concept of the study, worked on data collection, secured funding, carried out the research, managed the project, contributed to the study design, provided resources, supervised the work, checked the accuracy of the work, and contributed to reviewing and editing the manuscript.

## CONSENT STATEMENT

All human participants or their legal guardian(s) provided informed consent for the collection, analysis, and publication of their data in the manner presented.

## CONFLICT OF INTEREST STATEMENT

The authors would like to declare no conflicts of interest. Author disclosures are available in the Supporting Information.

## ABC‐DS Investigator Teams by Site

Columbia University Medical Center:

Nicole Schupf, PhD, DrPH

Adam M. Brickman, PhD

Howard F. Andrews, PhD

Joseph Hyungwoo Lee, PhD

Karen Bell, MD

Lawrence Honig, MD

William Charles Kreisl, MD

Lorraine Clark, PhD

Patrick Lao, PhD

Badri Vardarajan, PhD

Batool Rizvi, MS

Aubrey Johnson, BA

## Georgetown University

Amrita Cheema, PhD

## Hackensack University Medical Center

Benjamin Tycko, MD, PhD

## Johns Hopkins University, Bloomberg School of Public Health

Mei‐Cheng Wang, PhD

Yuchen Yang, MS

## Mass General Hospital/Harvard

Herminia Diana Rosas, MD

Florence Lai, MD

Julie C. Price, PhD

Margaret Pulsifer, PhD

Courtney Jordan, BSN, RN, CPN

Nusrat Jahan, BA

## New York State Institute of Basic Research

Sharon J. Krinsky‐McHale, PhD

Deborah Pang, MPH

## University of California Irvine

Ira T. Lott, MD

Wayne P. Silverman, PhD

Elizabeth Head, PhD

Mark Mapstone, PhD

David Keator, PhD

Michael A. Yassa, PhD

Christy Hom, PhD

Minodora Totoiu, MD, PhD

Eric W. Doran, MS

Dana D. Nguyen, PhD

Mithra Sathishkumar, MS

Alicia Hernandez, AA

## University of Cambridge, UK

Shahid Zaman, MD, PhD

Isabel Clare, PhD

Guy Williams, PhD

Timothy Fryer, PhD

Young Hong, PhD

Franklin Aigbirhio, PhD

Victoria Lupson, PhD

Monika Grigorova, MSc

## University of North Texas Health Sciences Center

Sid O'Bryant, PhD

Fan Zhang, PhD

James Richard Hall, PhD

Melissa Petersen, PhD

## University of Pittsburgh

Benjamin L. Handen, PhD

William E. Klunk, MD, PhD

Ann D. Cohen, PhD

Charles Laymon, PhD

Dana L. Tudorascu, PhD

Peter Bulova, MD

Milos D. Ikonomovic, MD

Eleanor Feingold, PhD

Julia Kofler, MD

Neelesh Nadkarni, MD

Davneet Singh Minhas, PhD

Howard J. Aizenstein, MD, PhD

Eleanor Feingold, PhD

Chester A. Mathis, PhD

Rameshwari Tumuluru, MD

Leslie Dunn, MPH

Joni Vander Bilt, MPH

Cathleen Wolfe, MEd

## University of Wisconsin

Bradley T. Christian, PhD

Sigan L. Hartley, PhD

Sterling C. Johnson, PhD

Rasmus M. Birn, PhD

Andrew Alexander, PhD

Matthew Zammit, MS

Brianna Gambetti, BS

Aleshia Cole, NP

Renee Makuch, BA

## Washington University, St Louis

Beau M. Ances, MD, PhD

John N. Constantino, MD

Desiree A. White, PhD

Olga Del Rosario MD

Elizabeth Westerhaus, MA

Anne Fagan, PhD

Rachel Henson, PhD

## Barrow Neurological Institute

Marwan N. Sabbagh, MD

Sandy Quintanilla, AA

## ABC‐DS Infrastructure

**Alzheimer's Therapeutic Research Institute (ATRI)**

5

Paul S. Aisen, MD

Michael Rafii, MD, PhD

Gustavo Jimenez, MBS

Sarah Walter, MSc

Devon Gessert, BS

Renarda Jones, MS

## Laboratory of Neuro Imaging (LONI)

Arthur W. Toga, PhD

Karen Crawford, MLIS

## National Centralized Repository for Alzheimer's Disease and Related Dementias (NCRAD)

Tatiana M. Foroud, PhD

Kelley Faber, MS, CCRC

Kristi Wilmes, MS, CCRP

Ariel Quickery, MS

Madison Donoho, BS

## Aging and Dementia Imaging Research (ADIR), Mayo Clinic

Gregory M. Preboske, MS

Bret J. Borowski, RT

Kejal Kantarci, MD

Clifford R. Jack Jr, MD

## PET QC Lab University of Michigan

Robert A. Koeppe, PhD

## Supporting information




Supporting Information

Supporting Information

Supporting Information

Supporting Information

Supporting Information

Supporting Information

Supporting Information

Supporting Information

Supporting Information
