# Supplementary material for: Structural MRI across lifespan reveals differential thalamic trajectories in Down syndrome
Source: Alzheimers Dement. 2026 Jul 14;22(7):e71671. doi: 10.1002/alz.71671 (PMC13369009; doi:10.1002/alz.71671)

Supplementary Figure 2

*Associations between AD biomarkers and ‘shrinking’ nuclei identified in the main analysis; only significant covariations are shown after FDR-correction. pTau181 (a), pTau217 (b), and NfL (c) all negatively covary with thalamic nuclear volumes of interest. CRP (d) and total free recall (e) exhibit a positive covariance with the nuclei shown. Notably, there is no covariance between the volumes of any nuclei of interest and between amyloid-beta 40 (f), 42 (g), and their ratio (h); refer to supplementary table S4 for detailed statistics.*


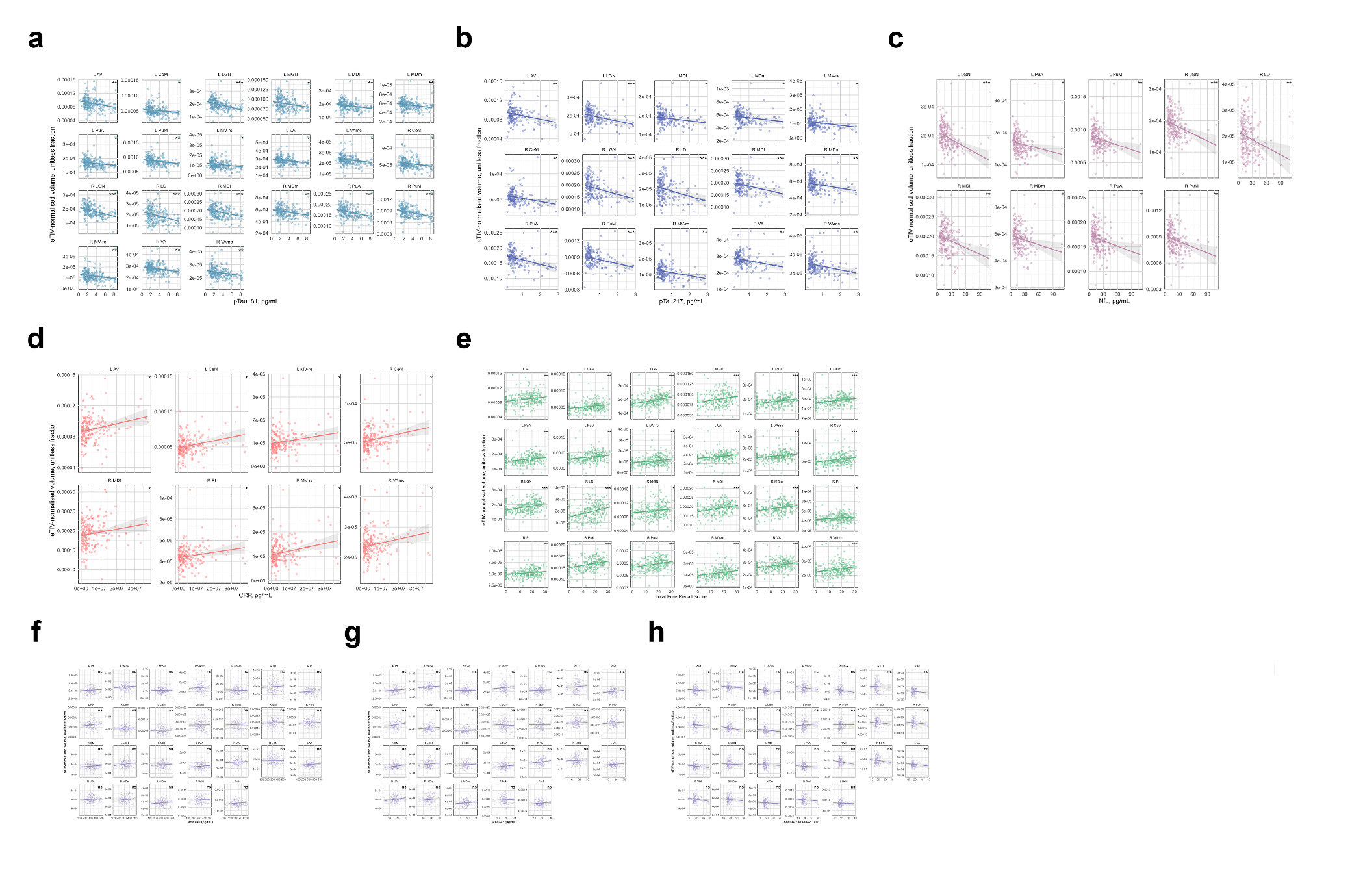

Supplement: Supplementary file 1 — Supporting Information [file ALZ-22-e71671-s003.docx]
