# Supplementary material for: Structural MRI across lifespan reveals differential thalamic trajectories in Down syndrome
Source: Alzheimers Dement. 2026 Jul 14;22(7):e71671. doi: 10.1002/alz.71671 (PMC13369009; doi:10.1002/alz.71671)
Supplement: Supplementary file 2 — Supporting Information [file ALZ-22-e71671-s007.docx]

Supplementary Figure 2


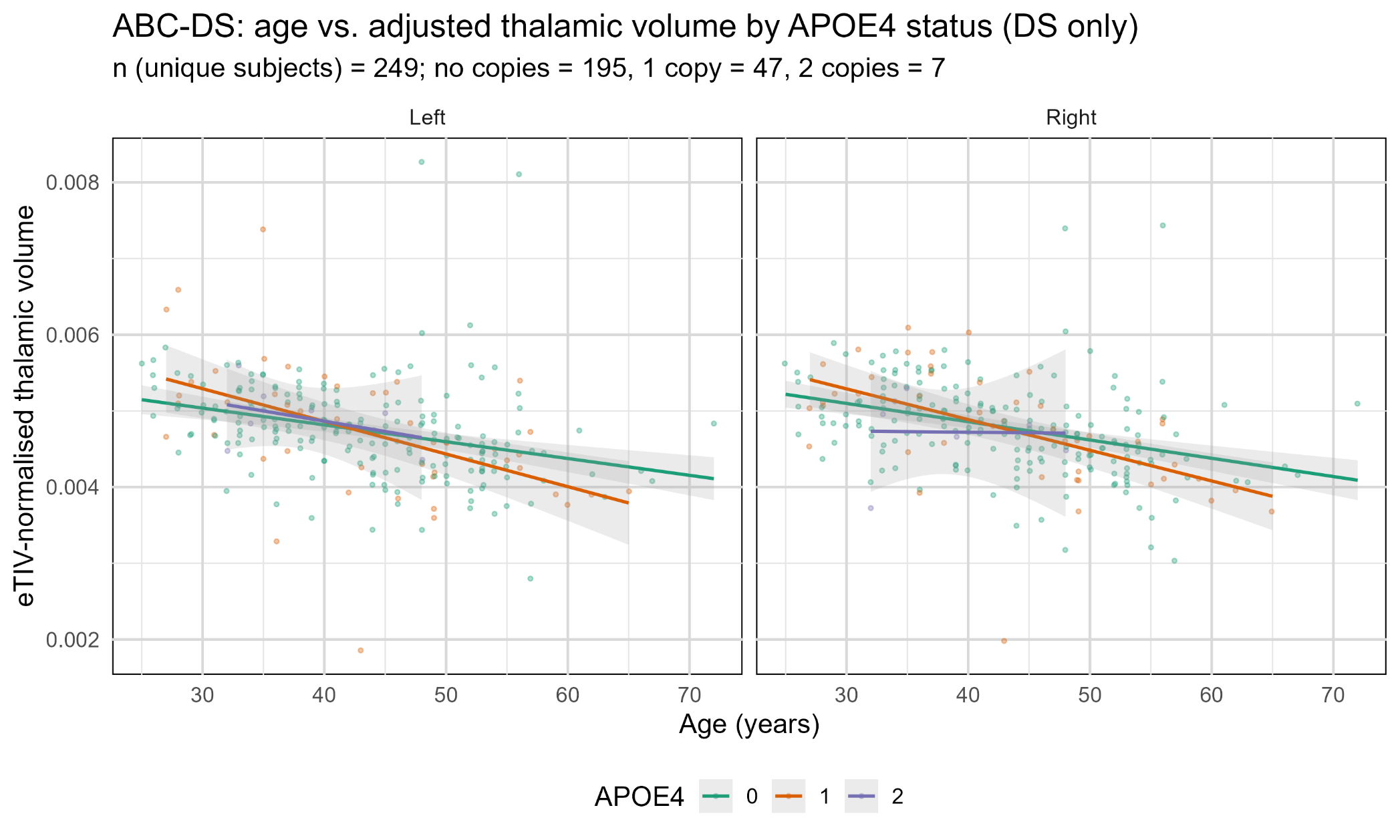
Scatterplots show eTIV‑normalized whole thalamic volume versus age, with jittered points and separate OLS regression lines (95% CI) for each APOE4 group, faceted by hemisphere. APOE4 copy number and thalamic trajectory interactions were non-significant. Refer to supplementary table S5 for further model statistics.
