# Supplementary material for: Structural MRI across lifespan reveals differential thalamic trajectories in Down syndrome
Source: Alzheimers Dement. 2026 Jul 14;22(7):e71671. doi: 10.1002/alz.71671 (PMC13369009; doi:10.1002/alz.71671)
Supplement: Supplementary file 3 — Supporting Information [file ALZ-22-e71671-s001.docx]

Table S1: Volumetric between-group results for both study cohorts.

|  | Sleep-DS | | | | | | |
| --- | --- | --- | --- | --- | --- | --- | --- |
| Measure | Control mean | DS mean | Control SD | DS SD | t-statistic | p-value | FDR-corrected p-value |
| Estimated total intracranial volume (eTIV), mm^3^ | 1396272.041 | 1205231.158 | 133339.004 | 139437.793 | 4.416 | 8.41×10⁻⁵ | 0.000420 |
| Gray matter (GM), mm^3^ | 624209.238 | 542616.007 | 67551.356 | 69014.750 | 3.763 | 0.000585 | 0.00146 |
| Whole thalamus, mm^3^ | 12864.522 | 11746.060 | 1148.473 | 1250.484 | 2.944 | 0.00554 | 0.00923 |
| Whole thalamus, eTIV-normalised, unitless fraction | 0.00920 | 0.00980 | 7.00×10⁻⁴ | 6.00×10⁻⁴ | -2.660 | 0.0117 | 0.0146 |
| Whole thalamus, GM-normalised | 0.0207 | 0.0217 | 0.00140 | 0.00120 | -2.557 | 0.0152 | 0.0152 |
|  | ABC-DS | | | | | | |
| Estimated total intracranial volume (eTIV), mm^3^ | 1420316.219 | 1298119.703 | 136039.989 | 182171.373 | 4.809 | 1.23×10⁻⁵ | 2.92×10⁻⁵ |
| Gray matter (GM), mm^3^ | 610674.628 | 544733.361 | 45805.295 | 59166.880 | 7.674 | 4.59×10⁻^10^ | 2.30×10⁻^9^ |
| Whole thalamus, mm^3^ | 13392.097 | 12202.752 | 1386.349 | 1448.749 | 4.786 | 1.75×10⁻⁵ | 2.92×10⁻⁵ |
| Whole thalamus, eTIV-normalised, unitless fraction | 0.00940 | 0.00950 | 7.00×10⁻⁴ | 0.00110 | -0.282 | 0.779 | 0.779 |
| Whole thalamus, GM-normalised | 0.0218 | 0.0224 | 0.00150 | 0.00200 | -2.421 | 0.0188 | 0.0235 |
