# Supplementary material for: Structural MRI across lifespan reveals differential thalamic trajectories in Down syndrome
Source: Alzheimers Dement. 2026 Jul 14;22(7):e71671. doi: 10.1002/alz.71671 (PMC13369009; doi:10.1002/alz.71671)
Supplement: Supplementary file 4 — Supporting Information [file ALZ-22-e71671-s006.docx]

Table S2: Group averages of eTIV-normalised thalamic nuclei volumes for both study cohorts with associated t-tests, effect sizes, and FDR-corrected p-values.

|  | | | Sleep-DS | | | | | | | | | | ABC-DS | | | | | | | | | |
| --- | --- | --- | --- | --- | --- | --- | --- | --- | --- | --- | --- | --- | --- | --- | --- | --- | --- | --- | --- | --- | --- | --- |
| Hemisphere | Nuclear Group | Nucleus | Control Mean | Control SD | DS Mean | DS SD | t-statistic | Pooled SD | Cohen’s d | FDR-corrected p-value | Significance | Control Mean | | Control SD | DS Mean | DS SD | t-statistic | Pooled SD | Cohen’s d | FDR-corrected p-value | Significance |  |
|  |  |  | Volume, unitless fraction of eTIV | | | |  |  |  |  |  | Volume, unitless fraction of eTIV | | | | |  |  |  |  |  |  |
| R | Ant. | AV | 9.9989e-05 | 9.6683e-06 | 1.01110e-04 | 1.5027e-05 | -0.28469 | 1.2908e-05 | 0.086715 | 0.86444 | ns | 1.00650e-04 | | 1.29080e-05 | 9.81230e-05 | 1.75920e-05 | 1.04550 | 1.709e-05 | -0.14799 | 0.40541 | ns |  |
| R | Lat. | LD | 2.5476e-05 | 6.9454e-06 | 2.5527e-05 | 7.1792e-06 | -0.02288 | 7.0755e-06 | 0.007246 | 0.98300 | ns | 2.16300e-05 | | 6.75830e-06 | 1.90310e-05 | 6.93720e-06 | 2.15160 | 6.9156e-06 | -0.37579 | 0.07058 | ns |  |
| R | Lat. | LP | 8.7219e-05 | 1.4916e-05 | 7.8848e-05 | 1.3787e-05 | 1.82670 | 1.4303e-05 | -0.58527 | 0.14654 | ns | 8.06380e-05 | | 1.17610e-05 | 6.50210e-05 | 1.46630e-05 | 7.20990 | 1.4341e-05 | -1.089 | 0.00000 | **** |  |
| R | Vent. | VA | 2.82440e-04 | 2.4696e-05 | 2.91750e-04 | 2.6708e-05 | -1.14280 | 2.5827e-05 | 0.3603 | 0.34211 | ns | 2.87560e-04 | | 3.18280e-05 | 2.77540e-04 | 4.22790e-05 | 1.68850 | 4.1146e-05 | -0.24348 | 0.16741 | ns |  |
| R | Vent. | VAmc | 2.1126e-05 | 2.0781e-06 | 2.2561e-05 | 2.0679e-06 | -2.17800 | 2.0724e-06 | 0.69257 | 0.08571 | ns | 2.39920e-05 | | 2.97860e-06 | 2.41600e-05 | 4.29240e-06 | -0.29662 | 4.1545e-06 | 0.040343 | 0.80000 | ns |  |
| R | Vent. | VLa | 4.14300e-04 | 2.7245e-05 | 4.16070e-04 | 2.7117e-05 | -0.20498 | 2.7175e-05 | 0.065178 | 0.91196 | ns | 4.26890e-04 | | 4.21090e-05 | 4.01720e-04 | 5.60260e-05 | 3.20510 | 5.4519e-05 | -0.46164 | 0.00636 | ** |  |
| R | Vent. | VLp | 5.34790e-04 | 3.4317e-05 | 5.34240e-04 | 3.5197e-05 | 0.04941 | 3.4806e-05 | -0.01566 | 0.98300 | ns | 5.50950e-04 | | 5.25470e-05 | 5.19320e-04 | 7.34460e-05 | 3.19460 | 7.1226e-05 | -0.44405 | 0.00636 | ** |  |
| R | Vent. | VM | 1.2859e-05 | 9.9664e-07 | 1.4165e-05 | 1.0672e-06 | -3.99330 | 1.0362e-06 | 1.2603 | 0.00148 | ** | 1.53290e-05 | | 2.76460e-06 | 1.59840e-05 | 2.49320e-06 | -1.34450 | 2.5279e-06 | 0.25885 | 0.26571 | ns |  |
| R | Vent. | VPL | 5.67080e-04 | 4.0671e-05 | 5.92930e-04 | 4.8053e-05 | -1.84280 | 4.49e-05 | 0.57583 | 0.14640 | ns | 6.02150e-04 | | 6.94450e-05 | 6.03180e-04 | 8.24720e-05 | -0.08115 | 8.0995e-05 | 0.012707 | 0.93600 | ns |  |
| R | Intra. | CL | 2.4531e-05 | 4.6204e-06 | 2.779e-05 | 4.4097e-06 | -2.26540 | 4.5052e-06 | 0.72345 | 0.07425 | ns | 2.33930e-05 | | 5.78800e-06 | 2.71200e-05 | 8.67300e-06 | -3.36320 | 8.3745e-06 | 0.44502 | 0.00422 | ** |  |
| R | Intra. | CM | 1.55830e-04 | 1.3765e-05 | 1.81220e-04 | 1.7146e-05 | -5.19520 | 1.5724e-05 | 1.6149 | 6.9833e-05 | **** | 1.67630e-04 | | 2.07320e-05 | 1.82090e-04 | 3.03440e-05 | -3.66140 | 2.9341e-05 | 0.49253 | 0.00207 | ** |  |
| R | Intra. | CeM | 4.3468e-05 | 6.5905e-06 | 4.7821e-05 | 1.019e-05 | -1.62970 | 8.7644e-06 | 0.4966 | 0.18667 | ns | 5.34860e-05 | | 9.90570e-06 | 5.42230e-05 | 1.52190e-05 | -0.38637 | 1.4674e-05 | 0.050242 | 0.76196 | ns |  |
| R | Intra. | Pc | 3.0123e-06 | 3.9068e-07 | 2.8387e-06 | 4.0087e-07 | 1.38180 | 3.9634e-07 | -0.43802 | 0.24444 | ns | 3.09170e-06 | | 4.59330e-07 | 2.95730e-06 | 6.33210e-07 | 1.55730 | 6.1465e-07 | -0.21862 | 0.18939 | ns |  |
| R | Intra. | Pf | 3.6429e-05 | 3.9315e-06 | 4.3307e-05 | 5.1842e-06 | -4.76820 | 4.6655e-06 | 1.4741 | 0.00017 | *** | 4.00370e-05 | | 6.19400e-06 | 4.50480e-05 | 9.27770e-06 | -4.22650 | 8.9586e-06 | 0.5594 | 0.00051 | *** |  |
| R | Med. | MDl | 1.92820e-04 | 1.4063e-05 | 2.01070e-04 | 2.3338e-05 | -1.37960 | 1.9735e-05 | 0.41794 | 0.24444 | ns | 2.06450e-04 | | 2.34360e-05 | 1.90310e-04 | 3.32650e-05 | 3.64130 | 3.2227e-05 | -0.50061 | 0.00209 | ** |  |
| R | Med. | MDm | 5.41740e-04 | 4.2865e-05 | 5.72720e-04 | 6.9255e-05 | -1.73190 | 5.8928e-05 | 0.52583 | 0.16411 | ns | 5.79020e-04 | | 6.10250e-05 | 5.59810e-04 | 9.11820e-05 | 1.64500 | 8.8059e-05 | -0.2181 | 0.16935 | ns |  |
| R | Med. | MV-re | 8.3759e-06 | 2.0627e-06 | 9.6249e-06 | 3.2365e-06 | -1.47960 | 2.7735e-06 | 0.45032 | 0.23125 | ns | 1.15710e-05 | | 2.64340e-06 | 1.16280e-05 | 4.84150e-06 | -0.10638 | 4.6296e-06 | 0.012305 | 0.93469 | ns |  |
| R | Med. | Pt | 4.676e-06 | 4.3359e-07 | 5.1592e-06 | 5.7945e-07 | -3.01360 | 5.1929e-07 | 0.93045 | 0.01765 | * | 5.00810e-06 | | 6.30490e-07 | 5.17760e-06 | 9.81320e-07 | -1.39090 | 9.4553e-07 | 0.17925 | 0.24853 | ns |  |
| R | Post. | L-SG | 1.421e-05 | 3.6941e-06 | 2.2499e-05 | 6.2351e-06 | -5.21610 | 5.2526e-06 | 1.5781 | 6.9833e-05 | **** | 1.55290e-05 | | 4.44490e-06 | 2.16860e-05 | 7.28190e-06 | -7.06980 | 6.9978e-06 | 0.87982 | 0.00000 | **** |  |
| R | Post. | LGN | 1.87740e-04 | 2.0229e-05 | 1.98570e-04 | 2.7924e-05 | -1.41940 | 2.4779e-05 | 0.43691 | 0.24118 | ns | 1.83290e-04 | | 2.68230e-05 | 1.84850e-04 | 3.85840e-05 | -0.30606 | 3.7348e-05 | 0.04168 | 0.80000 | ns |  |
| R | Post. | MGN | 6.7584e-05 | 6.698e-06 | 8.8087e-05 | 1.5248e-05 | -5.67320 | 1.2188e-05 | 1.6821 | 4.775e-05 | **** | 8.08880e-05 | | 1.42280e-05 | 9.67330e-05 | 2.03420e-05 | -5.88110 | 1.9699e-05 | 0.80437 | 0.00000 | **** |  |
| R | Post. | PuA | 1.55030e-04 | 1.5326e-05 | 1.67470e-04 | 1.4271e-05 | -2.63360 | 1.4752e-05 | 0.84315 | 0.03906 | * | 1.61840e-04 | | 1.66280e-05 | 1.64880e-04 | 2.63120e-05 | -0.94215 | 2.533e-05 | 0.12005 | 0.44756 | ns |  |
| R | Post. | PuI | 1.90490e-04 | 2.6691e-05 | 2.06150e-04 | 2.8671e-05 | -1.78500 | 2.7803e-05 | 0.56317 | 0.15259 | ns | 1.75650e-04 | | 2.47570e-05 | 1.95180e-04 | 3.74710e-05 | -4.11090 | 3.616e-05 | 0.54016 | 0.00060 | *** |  |
| R | Post. | PuL | 1.33590e-04 | 1.9806e-05 | 1.33720e-04 | 1.6539e-05 | -0.02103 | 1.8074e-05 | 0.006806 | 0.98300 | ns | 1.27710e-04 | | 1.88100e-05 | 1.33530e-04 | 2.55250e-05 | -1.65240 | 2.4804e-05 | 0.23463 | 0.16935 | ns |  |
| R | Post. | PuM | 8.14740e-04 | 9.4328e-05 | 8.87150e-04 | 8.0078e-05 | -2.58340 | 8.6743e-05 | 0.83487 | 0.04206 | * | 8.15360e-04 | | 7.73240e-05 | 8.67450e-04 | 1.37110e-04 | -3.35950 | 0.000131 | 0.39675 | 0.00420 | ** |  |
| L | Ant. | AV | 8.8283e-05 | 1.1467e-05 | 8.7762e-05 | 9.4378e-06 | 0.15453 | 1.0395e-05 | -0.05009 | 0.93404 | ns | 9.31570e-05 | | 1.04890e-05 | 8.91540e-05 | 1.70650e-05 | 1.95190 | 1.6405e-05 | -0.24405 | 0.09929 | ns |  |
| L | Lat. | LD | 2.0784e-05 | 4.6725e-06 | 2.3555e-05 | 5.9576e-06 | -1.64830 | 5.4205e-06 | 0.5112 | 0.18621 | ns | 2.29240e-05 | | 5.70420e-06 | 1.86750e-05 | 7.09310e-06 | 4.04580 | 6.9386e-06 | -0.6123 | 0.00079 | *** |  |
| L | Lat. | LP | 8.783e-05 | 1.3246e-05 | 7.9584e-05 | 1.208e-05 | 2.03720 | 1.2615e-05 | -0.65361 | 0.10271 | ns | 8.90150e-05 | | 1.48100e-05 | 6.85130e-05 | 1.47620e-05 | 7.77460 | 1.4768e-05 | -1.3883 | 0.00000 | **** |  |
| L | Vent. | VA | 2.74590e-04 | 2.416e-05 | 2.78320e-04 | 2.5343e-05 | -0.47449 | 2.4821e-05 | 0.15007 | 0.73750 | ns | 2.81620e-04 | | 3.82660e-05 | 2.78480e-04 | 5.04670e-05 | 0.44168 | 4.9142e-05 | -0.06402 | 0.73333 | ns |  |
| L | Vent. | VAmc | 1.9574e-05 | 1.6817e-06 | 2.0446e-05 | 1.9439e-06 | -1.52100 | 1.8312e-06 | 0.47632 | 0.22097 | ns | 2.24360e-05 | | 2.64400e-06 | 2.29070e-05 | 4.35820e-06 | -0.90855 | 4.1869e-06 | 0.1126 | 0.44756 | ns |  |
| L | Vent. | VLa | 4.02860e-04 | 2.9692e-05 | 3.95950e-04 | 2.1742e-05 | 0.82356 | 2.5606e-05 | -0.26999 | 0.52073 | ns | 4.16910e-04 | | 3.79270e-05 | 3.99870e-04 | 5.89560e-05 | 2.32510 | 5.6809e-05 | -0.29991 | 0.04854 | * |  |
| L | Vent. | VLp | 5.23120e-04 | 3.8525e-05 | 5.18320e-04 | 2.4114e-05 | 0.46008 | 3.139e-05 | -0.15294 | 0.73750 | ns | 5.46490e-04 | | 4.68990e-05 | 5.21330e-04 | 7.42250e-05 | 2.76380 | 7.1454e-05 | -0.35212 | 0.01868 | * |  |
| L | Vent. | VM | 1.2419e-05 | 1.3739e-06 | 1.3905e-05 | 8.1761e-07 | -4.04100 | 1.1017e-06 | 1.3489 | 0.00186 | ** | 1.47300e-05 | | 2.10170e-06 | 1.57920e-05 | 2.59140e-06 | -2.74800 | 2.5367e-06 | 0.41849 | 0.01964 | * |  |
| L | Vent. | VPL | 5.53410e-04 | 4.4684e-05 | 5.84420e-04 | 3.4038e-05 | -2.42440 | 3.916e-05 | 0.79183 | 0.05605 | ns | 5.96060e-04 | | 5.53530e-05 | 6.03490e-04 | 8.58320e-05 | -0.69528 | 8.2719e-05 | 0.089835 | 0.58214 | ns |  |
| L | Intra. | CL | 1.9573e-05 | 3.2471e-06 | 2.6325e-05 | 3.7591e-06 | -6.09250 | 3.5392e-06 | 1.9077 | 1.405e-05 | **** | 2.38130e-05 | | 4.64490e-06 | 2.73370e-05 | 8.03410e-06 | -3.81170 | 7.7011e-06 | 0.45751 | 0.00123 | ** |  |
| L | Intra. | CM | 1.51590e-04 | 1.4056e-05 | 1.76180e-04 | 1.4508e-05 | -5.42370 | 1.4308e-05 | 1.7182 | 4.775e-05 | **** | 1.64380e-04 | | 1.78470e-05 | 1.82970e-04 | 2.81890e-05 | -5.36820 | 2.7139e-05 | 0.68488 | 0.00001 | **** |  |
| L | Intra. | CeM | 4.1024e-05 | 5.5375e-06 | 4.3434e-05 | 8.2059e-06 | -1.10420 | 7.1366e-06 | 0.33772 | 0.35513 | ns | 5.03390e-05 | | 8.31550e-06 | 5.20530e-05 | 1.58300e-05 | -1.00460 | 1.5115e-05 | 0.11341 | 0.41842 | ns |  |
| L | Intra. | Pc | 2.5725e-06 | 3.0574e-07 | 2.4181e-06 | 3.7616e-07 | 1.43260 | 3.4643e-07 | -0.44585 | 0.24118 | ns | 2.72480e-06 | | 3.47360e-07 | 2.64110e-06 | 5.84110e-07 | 1.22070 | 5.6062e-07 | -0.14928 | 0.31389 | ns |  |
| L | Intra. | Pf | 3.3339e-05 | 3.491e-06 | 3.9832e-05 | 4.9962e-06 | -4.82380 | 4.3871e-06 | 1.48 | 0.00017 | *** | 3.76010e-05 | | 4.94700e-06 | 4.29550e-05 | 7.81550e-06 | -5.57830 | 7.5245e-06 | 0.71156 | 0.00000 | **** |  |
| L | Med. | MDl | 1.92500e-04 | 1.6915e-05 | 1.97440e-04 | 2.1975e-05 | -0.80364 | 1.9871e-05 | 0.2488 | 0.52073 | ns | 1.96350e-04 | | 1.93140e-05 | 1.86510e-04 | 3.39990e-05 | 2.54890 | 3.2564e-05 | -0.30245 | 0.02955 | * |  |
| L | Med. | MDm | 5.35710e-04 | 4.5978e-05 | 5.80390e-04 | 5.9972e-05 | -2.66560 | 5.416e-05 | 0.82493 | 0.03733 | * | 5.74050e-04 | | 6.15690e-05 | 5.63070e-04 | 9.74160e-05 | 0.91934 | 9.3781e-05 | -0.11715 | 0.44756 | ns |  |
| L | Med. | MV-re | 7.269e-06 | 1.6736e-06 | 8.1523e-06 | 2.6193e-06 | -1.29190 | 2.246e-06 | 0.39326 | 0.27703 | ns | 1.03050e-05 | | 2.38950e-06 | 1.05960e-05 | 4.52410e-06 | -0.59493 | 4.3207e-06 | 0.067386 | 0.64419 | ns |  |
| L | Med. | Pt | 4.2232e-06 | 4.3271e-07 | 4.5971e-06 | 5.3201e-07 | -2.45110 | 4.9008e-07 | 0.7629 | 0.05278 | ns | 4.60550e-06 | | 5.55560e-07 | 4.90580e-06 | 8.57150e-07 | -2.80270 | 8.2629e-07 | 0.36339 | 0.01779 | * |  |
| L | Post. | L-SG | 1.5801e-05 | 5.1543e-06 | 2.7015e-05 | 6.6492e-06 | -6.00670 | 6.0264e-06 | 1.8608 | 1.405e-05 | **** | 1.57470e-05 | | 6.38230e-06 | 2.47880e-05 | 8.63510e-06 | -7.57080 | 8.3928e-06 | 1.0773 | 0.00000 | **** |  |
| L | Post. | LGN | 1.89330e-04 | 2.213e-05 | 2.10760e-04 | 2.4605e-05 | -2.89700 | 2.353e-05 | 0.91079 | 0.02232 | * | 1.85410e-04 | | 2.20000e-05 | 1.92290e-04 | 3.89390e-05 | -1.55950 | 3.7288e-05 | 0.1844 | 0.18939 | ns |  |
| L | Post. | MGN | 5.9202e-05 | 8.4909e-06 | 7.7073e-05 | 1.5217e-05 | -4.68820 | 1.2658e-05 | 1.4118 | 0.00024 | *** | 7.53540e-05 | | 1.69190e-05 | 8.81200e-05 | 1.70260e-05 | -4.23250 | 1.7013e-05 | 0.75037 | 0.00060 | *** |  |
| L | Post. | PuA | 1.63490e-04 | 1.6389e-05 | 1.73610e-04 | 1.3732e-05 | -2.08740 | 1.4979e-05 | 0.67547 | 0.09696 | ns | 1.62440e-04 | | 2.12410e-05 | 1.64480e-04 | 3.23570e-05 | -0.49939 | 3.1214e-05 | 0.065325 | 0.70341 | ns |  |
| L | Post. | PuI | 2.11860e-04 | 2.6558e-05 | 2.33130e-04 | 3.677e-05 | -2.12090 | 3.26e-05 | 0.65267 | 0.09227 | ns | 1.76700e-04 | | 3.86470e-05 | 1.93420e-04 | 5.40540e-05 | -2.29650 | 5.2418e-05 | 0.31906 | 0.05080 | ns |  |
| L | Post. | PuL | 1.55640e-04 | 1.9727e-05 | 1.52460e-04 | 2.3037e-05 | 0.46962 | 2.1619e-05 | -0.14692 | 0.73750 | ns | 1.22460e-04 | | 2.77440e-05 | 1.32440e-04 | 3.42140e-05 | -1.95840 | 3.3492e-05 | 0.2982 | 0.09929 | ns |  |
| L | Post. | PuM | 8.55370e-04 | 8.3034e-05 | 9.44980e-04 | 7.9628e-05 | -3.45870 | 8.1169e-05 | 1.104 | 0.00592 | ** | 8.09800e-04 | | 9.92330e-05 | 8.55460e-04 | 1.52820e-04 | -2.38710 | 0.000147 | 0.30989 | 0.04370 | * |  |
