# Supplementary material for: Structural MRI across lifespan reveals differential thalamic trajectories in Down syndrome
Source: Alzheimers Dement. 2026 Jul 14;22(7):e71671. doi: 10.1002/alz.71671 (PMC13369009; doi:10.1002/alz.71671)
Supplement: Supplementary file 5 — Supporting Information [file ALZ-22-e71671-s009.docx]

Table S3: Group averages of raw thalamic nuclei volumes (without adjustment for intracranial volume) for both study cohorts with associated t-tests.

|  | | | Sleep-DS | | | | | | | ABC-DS | | | | | | |
| --- | --- | --- | --- | --- | --- | --- | --- | --- | --- | --- | --- | --- | --- | --- | --- | --- |
| Hemisphere | Nuclear Group | Nucleus | Volume, mm^3^ | | | | t-statistic | FDR-corrected p-value | Significance | Volume, mm^3^ | | | | t-statistic | FDR-corrected p-value | Significance |
|  |  |  | Control Mean | Control SD | DS Mean | DS SD |  |  |  | Control Mean | Control SD | DS Mean | DS SD |  |  |  |
| R | Ant. | AV | 139.350 | 16.594 | 121.350 | 19.709 | 3.136 | 0.012 | * | 142.470 | 19.200 | 126.430 | 21.884 | 4.606 | 8.68e-05 | **** |
| R | Lat. | LD | 35.360 | 8.995 | 30.721 | 9.131 | 1.612 | 0.176 | ns | 30.840 | 10.314 | 24.437 | 8.618 | 3.553 | 0.002166 | ** |
| R | Lat. | LP | 121.890 | 22.710 | 94.916 | 18.891 | 4.027 | 0.002 | ** | 114.490 | 19.471 | 83.721 | 18.417 | 8.930 | 5.03E-10 | **** |
| R | Vent. | VA | 393.870 | 46.612 | 349.830 | 36.523 | 3.270 | 0.011 | * | 407.580 | 53.360 | 357.310 | 49.502 | 5.336 | 1.05e-05 | **** |
| R | Vent. | VAmc | 29.400 | 3.175 | 27.017 | 2.429 | 2.618 | 0.030 | * | 33.925 | 4.185 | 31.081 | 4.803 | 3.741 | 0.001143 | ** |
| R | Vent. | VLa | 578.030 | 62.054 | 499.660 | 49.939 | 4.332 | 0.001 | ** | 605.290 | 74.624 | 517.590 | 67.710 | 6.672 | 2.59e-07 | **** |
| R | Vent. | VLp | 745.930 | 77.724 | 641.800 | 66.359 | 4.499 | 0.001 | ** | 781.070 | 93.365 | 669.370 | 91.502 | 6.732 | 2.29e-07 | **** |
| R | Vent. | VM | 17.942 | 2.081 | 17.015 | 1.868 | 1.466 | 0.211 | ns | 21.725 | 4.119 | 20.647 | 3.381 | 1.500 | 0.19054 | ns |
| R | Vent. | VPL | 790.250 | 79.081 | 712.520 | 83.832 | 3.010 | 0.013 | * | 852.800 | 108.180 | 779.100 | 113.960 | 3.799 | 0.001048 | ** |
| R | Intra. | CL | 34.021 | 5.894 | 33.390 | 5.936 | 0.336 | 0.803 | ns | 33.206 | 8.727 | 34.984 | 10.885 | -1.106 | 0.3186 | ns |
| R | Intra. | CM | 216.860 | 21.605 | 217.500 | 25.170 | -0.086 | 0.932 | ns | 237.460 | 32.111 | 234.940 | 38.867 | 0.429 | 0.69792 | ns |
| R | Intra. | CeM | 60.223 | 7.535 | 56.922 | 10.306 | 1.168 | 0.321 | ns | 75.523 | 13.233 | 69.411 | 16.836 | 2.499 | 0.026897 | * |
| R | Intra. | Pc | 4.188 | 0.547 | 3.400 | 0.501 | 4.707 | 0.001 | ** | 4.384 | 0.719 | 3.804 | 0.745 | 4.505 | 0.000124 | *** |
| R | Intra. | Pf | 50.564 | 4.544 | 51.792 | 5.543 | -0.770 | 0.507 | ns | 56.833 | 10.045 | 58.162 | 11.952 | -0.724 | 0.52444 | ns |
| R | Med. | MDl | 269.060 | 30.433 | 241.850 | 36.020 | 2.589 | 0.030 | * | 291.710 | 30.266 | 244.850 | 39.640 | 8.330 | 5.03E-10 | **** |
| R | Med. | MDm | 754.330 | 75.365 | 687.460 | 96.639 | 2.458 | 0.039 | * | 818.460 | 81.219 | 720.300 | 107.360 | 6.489 | 2.29e-07 | **** |
| R | Med. | MV-re | 11.538 | 2.327 | 11.411 | 3.488 | 0.138 | 0.922 | ns | 16.350 | 3.616 | 14.802 | 5.574 | 2.220 | 0.050167 | ns |
| R | Med. | Pt | 6.505 | 0.660 | 6.199 | 0.879 | 1.258 | 0.292 | ns | 7.110 | 1.098 | 6.684 | 1.268 | 2.133 | 0.059219 | ns |
| R | Post. | L-SG | 19.891 | 5.730 | 27.162 | 7.906 | -3.366 | 0.009 | ** | 21.885 | 5.907 | 27.990 | 9.212 | -5.344 | 5.23e-06 | **** |
| R | Post. | LGN | 261.510 | 31.170 | 238.720 | 39.270 | 2.046 | 0.085 | ns | 259.980 | 43.892 | 238.250 | 48.305 | 2.744 | 0.015196 | * |
| R | Post. | MGN | 94.287 | 12.407 | 105.810 | 21.046 | -2.152 | 0.071 | ns | 114.120 | 18.130 | 124.330 | 23.438 | -3.038 | 0.00738 | ** |
| R | Post. | PuA | 215.490 | 21.272 | 201.220 | 23.350 | 2.020 | 0.087 | ns | 229.160 | 26.472 | 212.270 | 31.251 | 3.496 | 0.002174 | ** |
| R | Post. | PuI | 264.180 | 31.914 | 247.480 | 39.337 | 1.482 | 0.210 | ns | 249.630 | 44.011 | 250.870 | 43.145 | -0.158 | 0.875 | ns |
| R | Post. | PuL | 185.370 | 26.210 | 160.880 | 24.229 | 3.041 | 0.013 | * | 181.070 | 29.570 | 171.820 | 30.113 | 1.753 | 0.12343 | ns |
| R | Post. | PuM | 1130.100 | 105.530 | 1066.300 | 132.510 | 1.696 | 0.153 | ns | 1157.400 | 150.800 | 1117.100 | 164.900 | 1.483 | 0.19079 | ns |
| L | Ant. | AV | 122.870 | 17.458 | 105.690 | 16.612 | 3.165 | 0.012 | * | 132.050 | 17.802 | 114.610 | 19.548 | 5.430 | 6.61e-06 | **** |
| L | Lat. | LD | 28.835 | 6.377 | 28.425 | 8.102 | 0.179 | 0.914 | ns | 32.558 | 8.646 | 23.931 | 8.732 | 5.594 | 4.96e-06 | **** |
| L | Lat. | LP | 122.720 | 21.487 | 96.118 | 18.898 | 4.110 | 0.001 | ** | 126.440 | 24.151 | 88.246 | 18.247 | 9.124 | 5.03E-10 | **** |
| L | Vent. | VA | 383.170 | 47.737 | 334.710 | 43.666 | 3.319 | 0.010 | ** | 400.250 | 68.410 | 358.830 | 62.225 | 3.437 | 0.002708 | ** |
| L | Vent. | VAmc | 27.292 | 3.193 | 24.521 | 2.674 | 2.935 | 0.015 | * | 31.803 | 4.387 | 29.479 | 5.026 | 2.918 | 0.010192 | * |
| L | Vent. | VLa | 562.630 | 68.186 | 476.820 | 57.679 | 4.240 | 0.001 | ** | 591.820 | 76.372 | 515.410 | 71.876 | 5.658 | 4.77e-06 | **** |
| L | Vent. | VLp | 730.080 | 84.256 | 623.870 | 69.748 | 4.281 | 0.001 | ** | 775.220 | 91.808 | 671.770 | 88.603 | 6.353 | 5.29e-07 | **** |
| L | Vent. | VM | 17.260 | 1.818 | 16.734 | 1.944 | 0.883 | 0.451 | ns | 20.926 | 3.658 | 20.349 | 3.207 | 0.899 | 0.425 | ns |
| L | Vent. | VPL | 769.780 | 68.105 | 702.660 | 75.796 | 2.947 | 0.014 | * | 844.170 | 90.345 | 777.970 | 106.380 | 4.017 | 0.000521 | *** |
| L | Intra. | CL | 27.085 | 3.648 | 31.710 | 5.782 | -3.078 | 0.012 | * | 33.711 | 6.690 | 35.114 | 9.425 | -1.111 | 0.3186 | ns |
| L | Intra. | CM | 210.750 | 19.798 | 211.580 | 23.350 | -0.122 | 0.922 | ns | 233.050 | 30.441 | 235.900 | 35.385 | -0.513 | 0.66304 | ns |
| L | Intra. | CeM | 56.961 | 7.402 | 51.850 | 9.219 | 1.945 | 0.096 | ns | 71.255 | 11.978 | 66.768 | 18.407 | 1.944 | 0.085455 | ns |
| L | Intra. | Pc | 3.577 | 0.439 | 2.901 | 0.492 | 4.584 | 0.001 | ** | 3.858 | 0.528 | 3.399 | 0.689 | 4.679 | 6.25e-05 | **** |
| L | Intra. | Pf | 46.293 | 4.286 | 47.640 | 5.465 | -0.873 | 0.451 | ns | 53.375 | 8.476 | 55.458 | 10.113 | -1.345 | 0.23125 | ns |
| L | Med. | MDl | 268.650 | 32.928 | 237.660 | 35.954 | 2.840 | 0.017 | * | 277.520 | 25.891 | 239.390 | 36.244 | 7.813 | 1.83e-09 | **** |
| L | Med. | MDm | 745.730 | 75.751 | 696.440 | 84.525 | 1.943 | 0.096 | ns | 811.070 | 81.139 | 723.190 | 105.120 | 5.838 | 1.61e-06 | **** |
| L | Med. | MV-re | 10.027 | 1.941 | 9.656 | 2.844 | 0.489 | 0.698 | ns | 14.596 | 3.464 | 13.520 | 5.330 | 1.612 | 0.15556 | ns |
| L | Med. | Pt | 5.872 | 0.613 | 5.525 | 0.787 | 1.565 | 0.185 | ns | 6.529 | 0.914 | 6.318 | 1.043 | 1.272 | 0.25488 | ns |
| L | Post. | L-SG | 21.903 | 6.752 | 32.656 | 8.962 | -4.325 | 0.001 | ** | 22.140 | 8.467 | 32.033 | 10.978 | -6.298 | 3.71e-07 | **** |
| L | Post. | LGN | 263.530 | 33.330 | 253.490 | 37.406 | 0.897 | 0.451 | ns | 263.190 | 39.624 | 247.250 | 46.524 | 2.207 | 0.051452 | ns |
| L | Post. | MGN | 82.163 | 10.788 | 92.078 | 16.912 | -2.247 | 0.059 | ns | 106.230 | 21.457 | 113.420 | 20.575 | -1.891 | 0.095882 | ns |
| L | Post. | PuA | 227.210 | 22.245 | 209.040 | 27.430 | 2.314 | 0.052 | ns | 230.430 | 35.540 | 212.030 | 40.219 | 2.858 | 0.011593 | * |
| L | Post. | PuI | 294.920 | 41.461 | 280.090 | 48.852 | 1.038 | 0.383 | ns | 252.290 | 64.877 | 249.160 | 67.638 | 0.270 | 0.8051 | ns |
| L | Post. | PuL | 216.840 | 31.698 | 183.830 | 35.536 | 3.103 | 0.012 | * | 174.260 | 43.175 | 170.930 | 43.293 | 0.434 | 0.69792 | ns |
| L | Post. | PuM | 1188.200 | 106.660 | 1138.100 | 155.950 | 1.204 | 0.311 | ns | 1150.800 | 181.290 | 1103.700 | 195.940 | 1.444 | 0.19872 | ns |
