# Supplementary material for: Structural MRI across lifespan reveals differential thalamic trajectories in Down syndrome
Source: Alzheimers Dement. 2026 Jul 14;22(7):e71671. doi: 10.1002/alz.71671 (PMC13369009; doi:10.1002/alz.71671)
Supplement: Supplementary file 6 — Supporting Information [file ALZ-22-e71671-s008.docx]

Table S4: GLM model parameters for volumetrics within both cohorts.

| Region | GLM Term | Estimate (the visit_age:groupDS term estimate is the slope difference depicted in the corresponding figure 4) | Standard Error | Statistic | FDR-corrected p-value | Significance |
| --- | --- | --- | --- | --- | --- | --- |
| L AV | (Intercept) | 0.000116 | 1.6926e-05 | 6.8285 | 1.7442e-10 | *** |
| L AV | visit_age | 1.5709e-08 | 2.1801e-07 | 0.072057 | 0.9662 |  |
| L AV | groupDS | 1.6127e-05 | 1.078e-05 | 1.496 | 0.18857 |  |
| L AV | sexF | 3.6846e-06 | 2.2854e-06 | 1.6122 | 0.20775 |  |
| L AV | totalGray | -4.25E-11 | 1.9045e-11 | -2.23 | 0.1433 |  |
| L AV | visit_age:groupDS | -5.07E-07 | 2.4171e-07 | -2.0983 | 0.068079 |  |
| L CL | (Intercept) | 3.4257e-05 | 8.1788e-06 | 4.1885 | 4.8146e-05 | *** |
| L CL | visit_age | -5.50E-08 | 1.0535e-07 | -0.52232 | 0.9662 |  |
| L CL | groupDS | 4.4273e-06 | 5.2091e-06 | 0.84993 | 0.42596 |  |
| L CL | sexF | 2.1774e-06 | 1.1043e-06 | 1.9717 | 0.11425 |  |
| L CL | totalGray | -1.60E-11 | 9.2026e-12 | -1.7435 | 0.17925 |  |
| L CL | visit_age:groupDS | -2.92E-08 | 1.1679e-07 | -0.24984 | 0.80289 |  |
| L CM | (Intercept) | 0.000205 | 2.836e-05 | 7.2169 | 2.044e-11 | *** |
| L CM | visit_age | -9.04E-08 | 3.6529e-07 | -0.24751 | 0.9662 |  |
| L CM | groupDS | 4.1953e-05 | 1.8063e-05 | 2.3226 | 0.040986 | * |
| L CM | sexF | 5.7114e-06 | 3.8293e-06 | 1.4915 | 0.2326 |  |
| L CM | totalGray | -6.68E-11 | 3.191e-11 | -2.0928 | 0.1433 |  |
| L CM | visit_age:groupDS | -6.06E-07 | 4.0499e-07 | -1.4954 | 0.17427 |  |
| L CeM | (Intercept) | 6.4828e-05 | 1.5325e-05 | 4.2302 | 4.1532e-05 | *** |
| L CeM | visit_age | 3.6465e-08 | 1.9739e-07 | 0.18474 | 0.9662 |  |
| L CeM | groupDS | 2.526e-05 | 9.7605e-06 | 2.588 | 0.028928 | * |
| L CeM | sexF | 4.4006e-06 | 2.0692e-06 | 2.1267 | 0.11425 |  |
| L CeM | totalGray | -3.20E-11 | 1.7243e-11 | -1.8565 | 0.17925 |  |
| L CeM | visit_age:groupDS | -5.63E-07 | 2.1884e-07 | -2.5743 | 0.029318 | * |
| L L-SG | (Intercept) | 1.9458e-05 | 9.0096e-06 | 2.1597 | 0.032278 | * |
| L L-SG | visit_age | 2.6486e-08 | 1.1605e-07 | 0.22824 | 0.9662 |  |
| L L-SG | groupDS | 1.7329e-05 | 5.7382e-06 | 3.0199 | 0.012733 | * |
| L L-SG | sexF | 6.0843e-07 | 1.2165e-06 | 0.50015 | 0.67104 |  |
| L L-SG | totalGray | -8.70E-12 | 1.0137e-11 | -0.85798 | 0.50269 |  |
| L L-SG | visit_age:groupDS | -2.02E-07 | 1.2866e-07 | -1.5698 | 0.16798 |  |
| L LD | (Intercept) | 1.8682e-05 | 7.1972e-06 | 2.5957 | 0.010796 | * |
| L LD | visit_age | -5.63E-08 | 9.2702e-08 | -0.60774 | 0.9662 |  |
| L LD | groupDS | 3.8605e-06 | 4.5839e-06 | 0.84218 | 0.42596 |  |
| L LD | sexF | 1.1234e-06 | 9.7178e-07 | 1.156 | 0.336 |  |
| L LD | totalGray | 9.3036e-12 | 8.0981e-12 | 1.1489 | 0.41931 |  |
| L LD | visit_age:groupDS | -1.64E-07 | 1.0278e-07 | -1.5965 | 0.16397 |  |
| L LGN | (Intercept) | 0.000144 | 3.6242e-05 | 3.9704 | 0.000111 | *** |
| L LGN | visit_age | -4.59E-07 | 4.668e-07 | -0.98412 | 0.9662 |  |
| L LGN | groupDS | 6.7817e-05 | 2.3082e-05 | 2.9381 | 0.014894 | * |
| L LGN | sexF | 1.0149e-05 | 4.8934e-06 | 2.0739 | 0.11425 |  |
| L LGN | totalGray | 8.5766e-11 | 4.0778e-11 | 2.1032 | 0.1433 |  |
| L LGN | visit_age:groupDS | -1.19E-06 | 5.1753e-07 | -2.3047 | 0.047628 | * |
| L LP | (Intercept) | 8.9137e-05 | 1.5813e-05 | 5.6369 | 8.3724e-08 | *** |
| L LP | visit_age | -3.79E-07 | 2.0368e-07 | -1.8627 | 0.9662 |  |
| L LP | groupDS | -2.88E-05 | 1.0071e-05 | -2.8555 | 0.015238 | * |
| L LP | sexF | 5.1375e-06 | 2.1351e-06 | 2.4062 | 0.08323 |  |
| L LP | totalGray | 1.938e-11 | 1.7792e-11 | 1.0892 | 0.44674 |  |
| L LP | visit_age:groupDS | 2.6642e-07 | 2.2581e-07 | 1.1799 | 0.29153 |  |
| L MDl | (Intercept) | 0.000159 | 3.2519e-05 | 4.8851 | 2.7941e-06 | *** |
| L MDl | visit_age | 3.029e-07 | 4.1885e-07 | 0.72316 | 0.9662 |  |
| L MDl | groupDS | 6.0171e-05 | 2.0711e-05 | 2.9052 | 0.015229 | * |
| L MDl | sexF | 1.5021e-05 | 4.3908e-06 | 3.4211 | 0.023718 | * |
| L MDl | totalGray | 1.9358e-11 | 3.659e-11 | 0.52905 | 0.66181 |  |
| L MDl | visit_age:groupDS | -1.47E-06 | 4.6437e-07 | -3.1656 | 0.021454 | * |
| L MDm | (Intercept) | 0.00052 | 9.384e-05 | 5.5424 | 1.2655e-07 | *** |
| L MDm | visit_age | 4.3283e-07 | 1.2087e-06 | 0.3581 | 0.9662 |  |
| L MDm | groupDS | 0.00017 | 5.9767e-05 | 2.8375 | 0.015238 | * |
| L MDm | sexF | 4.0405e-05 | 1.2671e-05 | 3.1889 | 0.023718 | * |
| L MDm | totalGray | 2.6583e-12 | 1.0559e-10 | 0.025177 | 0.97993 |  |
| L MDm | visit_age:groupDS | -3.86E-06 | 1.34e-06 | -2.8772 | 0.026983 | * |
| L MGN | (Intercept) | 8.7107e-05 | 1.7689e-05 | 4.9245 | 2.4e-06 | *** |
| L MGN | visit_age | 3.4871e-08 | 2.2783e-07 | 0.15306 | 0.9662 |  |
| L MGN | groupDS | 3.7869e-05 | 1.1266e-05 | 3.3614 | 0.00544 | ** |
| L MGN | sexF | 2.224e-06 | 2.3883e-06 | 0.9312 | 0.45198 |  |
| L MGN | totalGray | -2.45E-11 | 1.9903e-11 | -1.2319 | 0.37761 |  |
| L MGN | visit_age:groupDS | -6.06E-07 | 2.5259e-07 | -2.3978 | 0.040818 | * |
| L MV-re | (Intercept) | 1.5604e-05 | 4.3468e-06 | 3.5898 | 0.000443 | *** |
| L MV-re | visit_age | -1.57E-08 | 5.5987e-08 | -0.2799 | 0.9662 |  |
| L MV-re | groupDS | 7.0453e-06 | 2.7685e-06 | 2.5449 | 0.028928 | * |
| L MV-re | sexF | 7.7903e-07 | 5.8691e-07 | 1.3273 | 0.27275 |  |
| L MV-re | totalGray | -8.56E-12 | 4.8909e-12 | -1.7495 | 0.17925 |  |
| L MV-re | visit_age:groupDS | -1.65E-07 | 6.2072e-08 | -2.6537 | 0.028598 | * |
| L Pc | (Intercept) | 3.7553e-06 | 5.6971e-07 | 6.5916 | 5.8982e-10 | *** |
| L Pc | visit_age | -5.57E-09 | 7.338e-09 | -0.75843 | 0.9662 |  |
| L Pc | groupDS | 4.3542e-07 | 3.6285e-07 | 1.2 | 0.28524 |  |
| L Pc | sexF | 1.4975e-07 | 7.6924e-08 | 1.9467 | 0.11425 |  |
| L Pc | totalGray | -1.49E-12 | 6.4103e-13 | -2.3178 | 0.1433 |  |
| L Pc | visit_age:groupDS | -1.33E-08 | 8.1355e-09 | -1.6317 | 0.15737 |  |
| L Pf | (Intercept) | 4.997e-05 | 8.0016e-06 | 6.245 | 3.8694e-09 | *** |
| L Pf | visit_age | -8.57E-08 | 1.0306e-07 | -0.83154 | 0.9662 |  |
| L Pf | groupDS | 7.0198e-06 | 5.0962e-06 | 1.3775 | 0.22297 |  |
| L Pf | sexF | 1.3578e-06 | 1.0804e-06 | 1.2567 | 0.29984 |  |
| L Pf | totalGray | -1.59E-11 | 9.0032e-12 | -1.7679 | 0.17925 |  |
| L Pf | visit_age:groupDS | -5.37E-08 | 1.1426e-07 | -0.46996 | 0.65178 |  |
| L Pt | (Intercept) | 6.1581e-06 | 8.6065e-07 | 7.1551 | 2.567e-11 | *** |
| L Pt | visit_age | -1.86E-09 | 1.1085e-08 | -0.16757 | 0.9662 |  |
| L Pt | groupDS | 1.1243e-06 | 5.4815e-07 | 2.0511 | 0.070998 |  |
| L Pt | sexF | 8.8042e-08 | 1.1621e-07 | 0.75763 | 0.53489 |  |
| L Pt | totalGray | -2.51E-12 | 9.6838e-13 | -2.5905 | 0.126 |  |
| L Pt | visit_age:groupDS | -2.26E-08 | 1.229e-08 | -1.836 | 0.11622 |  |
| L PuA | (Intercept) | 0.00012 | 3.1404e-05 | 3.8139 | 0.000195 | *** |
| L PuA | visit_age | 3.6129e-07 | 4.0449e-07 | 0.8932 | 0.9662 |  |
| L PuA | groupDS | 7.5162e-05 | 2.0001e-05 | 3.7579 | 0.004378 | ** |
| L PuA | sexF | 8.9401e-06 | 4.2402e-06 | 2.1084 | 0.11425 |  |
| L PuA | totalGray | 3.1891e-11 | 3.5335e-11 | 0.90254 | 0.49667 |  |
| L PuA | visit_age:groupDS | -1.57E-06 | 4.4845e-07 | -3.5073 | 0.018293 | * |
| L PuI | (Intercept) | 0.000129 | 5.3256e-05 | 2.4249 | 0.016955 | * |
| L PuI | visit_age | -8.22E-07 | 6.8594e-07 | -1.1982 | 0.9662 |  |
| L PuI | groupDS | 6.698e-05 | 3.3918e-05 | 1.9747 | 0.079467 |  |
| L PuI | sexF | 2.1174e-05 | 7.1907e-06 | 2.9446 | 0.029186 | * |
| L PuI | totalGray | 1.06e-10 | 5.9922e-11 | 1.769 | 0.17925 |  |
| L PuI | visit_age:groupDS | -8.19E-07 | 7.6049e-07 | -1.0764 | 0.33649 |  |
| L PuL | (Intercept) | 0.000173 | 3.5673e-05 | 4.8496 | 3.1944e-06 | *** |
| L PuL | visit_age | -7.65E-08 | 4.5948e-07 | -0.1665 | 0.9662 |  |
| L PuL | groupDS | 3.742e-05 | 2.272e-05 | 1.647 | 0.14803 |  |
| L PuL | sexF | -2.97E-07 | 4.8166e-06 | -0.06167 | 0.97028 |  |
| L PuL | totalGray | -7.63E-11 | 4.0138e-11 | -1.9002 | 0.17925 |  |
| L PuL | visit_age:groupDS | -7.67E-07 | 5.0941e-07 | -1.5049 | 0.17427 |  |
| L PuM | (Intercept) | 0.00067 | 0.000147 | 4.5658 | 1.0814e-05 | *** |
| L PuM | visit_age | -1.44E-07 | 1.891e-06 | -0.07607 | 0.9662 |  |
| L PuM | groupDS | 0.000325 | 9.3507e-05 | 3.4751 | 0.004423 | ** |
| L PuM | sexF | 4.8637e-05 | 1.9823e-05 | 2.4535 | 0.08323 |  |
| L PuM | totalGray | 1.706e-10 | 1.6519e-10 | 1.0327 | 0.45025 |  |
| L PuM | visit_age:groupDS | -5.81E-06 | 2.0965e-06 | -2.7707 | 0.027108 | * |
| L VA | (Intercept) | 0.000289 | 5.0366e-05 | 5.7476 | 5.0855e-08 | *** |
| L VA | visit_age | 8.7136e-08 | 6.4873e-07 | 0.13432 | 0.9662 |  |
| L VA | groupDS | 7.8694e-05 | 3.2078e-05 | 2.4532 | 0.035147 | * |
| L VA | sexF | 1.1225e-05 | 6.8005e-06 | 1.6506 | 0.19986 |  |
| L VA | totalGray | -3.40E-11 | 5.667e-11 | -0.60047 | 0.63799 |  |
| L VA | visit_age:groupDS | -1.87E-06 | 7.1922e-07 | -2.5939 | 0.029318 | * |
| L VAmc | (Intercept) | 3.1884e-05 | 4.1729e-06 | 7.6407 | 2.1017e-12 | *** |
| L VAmc | visit_age | -4.74E-08 | 5.3748e-08 | -0.88118 | 0.9662 |  |
| L VAmc | groupDS | 5.3106e-06 | 2.6577e-06 | 1.9982 | 0.077757 |  |
| L VAmc | sexF | 7.5131e-07 | 5.6344e-07 | 1.3335 | 0.27275 |  |
| L VAmc | totalGray | -1.30E-11 | 4.6952e-12 | -2.7766 | 0.126 |  |
| L VAmc | visit_age:groupDS | -1.28E-07 | 5.9589e-08 | -2.1454 | 0.063027 |  |
| L VLa | (Intercept) | 0.000491 | 5.9375e-05 | 8.273 | 6.4757e-14 | *** |
| L VLa | visit_age | -5.14E-07 | 7.6477e-07 | -0.67223 | 0.9662 |  |
| L VLa | groupDS | 1.8855e-05 | 3.7816e-05 | 0.49861 | 0.63106 |  |
| L VLa | sexF | 1.5696e-05 | 8.017e-06 | 1.9579 | 0.11425 |  |
| L VLa | totalGray | -1.06E-10 | 6.6807e-11 | -1.5844 | 0.21967 |  |
| L VLa | visit_age:groupDS | -8.78E-07 | 8.4788e-07 | -1.0357 | 0.34228 |  |
| L VLp | (Intercept) | 0.000679 | 7.4815e-05 | 9.0793 | 9.6244e-16 | *** |
| L VLp | visit_age | -8.06E-07 | 9.6364e-07 | -0.8364 | 0.9662 |  |
| L VLp | groupDS | -4.93E-06 | 4.765e-05 | -0.10344 | 0.91768 |  |
| L VLp | sexF | 1.9068e-05 | 1.0102e-05 | 1.8876 | 0.1252 |  |
| L VLp | totalGray | -1.85E-10 | 8.418e-11 | -2.1965 | 0.1433 |  |
| L VLp | visit_age:groupDS | -6.16E-07 | 1.0684e-06 | -0.57658 | 0.60072 |  |
| L VM | (Intercept) | 1.8168e-05 | 2.7347e-06 | 6.6433 | 4.613e-10 | *** |
| L VM | visit_age | -1.01E-08 | 3.5224e-08 | -0.28611 | 0.9662 |  |
| L VM | groupDS | 1.634e-06 | 1.7417e-06 | 0.93815 | 0.38774 |  |
| L VM | sexF | 2.6132e-07 | 3.6925e-07 | 0.7077 | 0.54513 |  |
| L VM | totalGray | -5.23E-12 | 3.077e-12 | -1.6988 | 0.18846 |  |
| L VM | visit_age:groupDS | -1.97E-08 | 3.9052e-08 | -0.50542 | 0.63923 |  |
| L VPL | (Intercept) | 0.000736 | 8.7445e-05 | 8.4217 | 3.1437e-14 | *** |
| L VPL | visit_age | -4.49E-07 | 1.1263e-06 | -0.39871 | 0.9662 |  |
| L VPL | groupDS | 5.3114e-05 | 5.5694e-05 | 0.95368 | 0.38757 |  |
| L VPL | sexF | 1.3284e-05 | 1.1807e-05 | 1.1251 | 0.3441 |  |
| L VPL | totalGray | -2.14E-10 | 9.8391e-11 | -2.1788 | 0.1433 |  |
| L VPL | visit_age:groupDS | -1.30E-06 | 1.2487e-06 | -1.0448 | 0.34228 |  |
| R AV | (Intercept) | 0.000101 | 1.7779e-05 | 5.6868 | 6.716e-08 | *** |
| R AV | visit_age | -4.09E-08 | 2.29e-07 | -0.17855 | 0.9662 |  |
| R AV | groupDS | 1.4783e-05 | 1.1323e-05 | 1.3056 | 0.24713 |  |
| R AV | sexF | 7.1578e-06 | 2.4005e-06 | 2.9818 | 0.029186 | * |
| R AV | totalGray | -7.55E-12 | 2.0004e-11 | -0.37766 | 0.72037 |  |
| R AV | visit_age:groupDS | -3.55E-07 | 2.5388e-07 | -1.3982 | 0.20394 |  |
| R CL | (Intercept) | 2.5058e-05 | 9.0309e-06 | 2.7747 | 0.006548 | ** |
| R CL | visit_age | 1.7287e-08 | 1.1632e-07 | 0.14862 | 0.9662 |  |
| R CL | groupDS | 9.2218e-06 | 5.7518e-06 | 1.6033 | 0.15712 |  |
| R CL | sexF | 1.6941e-06 | 1.2194e-06 | 1.3893 | 0.26247 |  |
| R CL | totalGray | -6.20E-12 | 1.0161e-11 | -0.61025 | 0.63799 |  |
| R CL | visit_age:groupDS | -1.24E-07 | 1.2896e-07 | -0.95939 | 0.37575 |  |
| R CM | (Intercept) | 0.000187 | 3.0475e-05 | 6.1247 | 7.2164e-09 | *** |
| R CM | visit_age | 3.1912e-07 | 3.9252e-07 | 0.81299 | 0.9662 |  |
| R CM | groupDS | 6.4658e-05 | 1.9409e-05 | 3.3313 | 0.00544 | ** |
| R CM | sexF | 4.8784e-06 | 4.1147e-06 | 1.1856 | 0.32886 |  |
| R CM | totalGray | -5.98E-11 | 3.4289e-11 | -1.7428 | 0.17925 |  |
| R CM | visit_age:groupDS | -1.22E-06 | 4.3518e-07 | -2.8142 | 0.027108 | * |
| R CeM | (Intercept) | 7.7133e-05 | 1.446e-05 | 5.3342 | 3.5123e-07 | *** |
| R CeM | visit_age | -7.51E-08 | 1.8625e-07 | -0.40345 | 0.9662 |  |
| R CeM | groupDS | 2.1844e-05 | 9.2095e-06 | 2.3719 | 0.039921 | * |
| R CeM | sexF | 4.766e-06 | 1.9524e-06 | 2.4411 | 0.08323 |  |
| R CeM | totalGray | -3.96E-11 | 1.627e-11 | -2.4318 | 0.1433 |  |
| R CeM | visit_age:groupDS | -5.16E-07 | 2.0649e-07 | -2.5001 | 0.034167 | * |
| R L-SG | (Intercept) | 1.722e-05 | 7.5512e-06 | 2.2805 | 0.024296 | * |
| R L-SG | visit_age | 8.5177e-08 | 9.7262e-08 | 0.87576 | 0.9662 |  |
| R L-SG | groupDS | 1.4501e-05 | 4.8094e-06 | 3.0152 | 0.012733 | * |
| R L-SG | sexF | 7.661e-09 | 1.0196e-06 | 0.007514 | 0.99401 |  |
| R L-SG | totalGray | -8.71E-12 | 8.4965e-12 | -1.0251 | 0.45025 |  |
| R L-SG | visit_age:groupDS | -2.08E-07 | 1.0783e-07 | -1.931 | 0.09728 |  |
| R LD | (Intercept) | 1.4806e-05 | 7.2842e-06 | 2.0326 | 0.043026 | * |
| R LD | visit_age | 5.0189e-08 | 9.3822e-08 | 0.53494 | 0.9662 |  |
| R LD | groupDS | 8.6022e-06 | 4.6393e-06 | 1.8542 | 0.10117 |  |
| R LD | sexF | 7.272e-07 | 9.8353e-07 | 0.73937 | 0.53522 |  |
| R LD | totalGray | 6.5814e-12 | 8.196e-12 | 0.80301 | 0.5283 |  |
| R LD | visit_age:groupDS | -2.44E-07 | 1.0402e-07 | -2.3417 | 0.045204 | * |
| R LGN | (Intercept) | 0.000159 | 3.6494e-05 | 4.3577 | 2.487e-05 | *** |
| R LGN | visit_age | -5.30E-08 | 4.7005e-07 | -0.11267 | 0.9662 |  |
| R LGN | groupDS | 8.1132e-05 | 2.3243e-05 | 3.4906 | 0.004423 | ** |
| R LGN | sexF | 4.2017e-06 | 4.9275e-06 | 0.85271 | 0.49318 |  |
| R LGN | totalGray | 3.7387e-11 | 4.1062e-11 | 0.91051 | 0.49667 |  |
| R LGN | visit_age:groupDS | -1.76E-06 | 5.2113e-07 | -3.3679 | 0.018293 | * |
| R LP | (Intercept) | 6.8431e-05 | 1.5289e-05 | 4.4759 | 1.5332e-05 | *** |
| R LP | visit_age | -6.12E-08 | 1.9692e-07 | -0.3106 | 0.9662 |  |
| R LP | groupDS | -5.08E-06 | 9.7373e-06 | -0.52139 | 0.6276 |  |
| R LP | sexF | 4.7216e-06 | 2.0643e-06 | 2.2873 | 0.095496 |  |
| R LP | totalGray | 1.77e-11 | 1.7202e-11 | 1.0289 | 0.45025 |  |
| R LP | visit_age:groupDS | -1.76E-07 | 2.1832e-07 | -0.80828 | 0.45609 |  |
| R MDl | (Intercept) | 0.000162 | 3.284e-05 | 4.925 | 2.4e-06 | *** |
| R MDl | visit_age | 4.9021e-08 | 4.2299e-07 | 0.11589 | 0.9662 |  |
| R MDl | groupDS | 3.6944e-05 | 2.0916e-05 | 1.7663 | 0.11882 |  |
| R MDl | sexF | 1.4443e-05 | 4.4342e-06 | 3.2571 | 0.023718 | * |
| R MDl | totalGray | 4.9589e-11 | 3.6951e-11 | 1.342 | 0.33456 |  |
| R MDl | visit_age:groupDS | -1.03E-06 | 4.6896e-07 | -2.1997 | 0.058053 |  |
| R MDm | (Intercept) | 0.000499 | 8.8991e-05 | 5.6056 | 9.4729e-08 | *** |
| R MDm | visit_age | 3.9837e-07 | 1.1462e-06 | 0.34755 | 0.9662 |  |
| R MDm | groupDS | 0.000144 | 5.6678e-05 | 2.5415 | 0.028928 | * |
| R MDm | sexF | 3.7673e-05 | 1.2016e-05 | 3.1353 | 0.023718 | * |
| R MDm | totalGray | 5.1293e-11 | 1.0013e-10 | 0.51227 | 0.66181 |  |
| R MDm | visit_age:groupDS | -3.40E-06 | 1.2708e-06 | -2.6721 | 0.028598 | * |
| R MGN | (Intercept) | 9.3183e-05 | 2.0428e-05 | 4.5616 | 1.0814e-05 | *** |
| R MGN | visit_age | 2.0333e-07 | 2.6311e-07 | 0.77278 | 0.9662 |  |
| R MGN | groupDS | 5.1345e-05 | 1.301e-05 | 3.9465 | 0.004378 | ** |
| R MGN | sexF | 2.1909e-06 | 2.7582e-06 | 0.79432 | 0.52155 |  |
| R MGN | totalGray | -3.71E-11 | 2.2985e-11 | -1.6154 | 0.21466 |  |
| R MGN | visit_age:groupDS | -8.68E-07 | 2.9171e-07 | -2.9763 | 0.026412 | * |
| R MV-re | (Intercept) | 1.8805e-05 | 4.6075e-06 | 4.0815 | 7.2834e-05 | *** |
| R MV-re | visit_age | -2.68E-08 | 5.9346e-08 | -0.4518 | 0.9662 |  |
| R MV-re | groupDS | 7.031e-06 | 2.9345e-06 | 2.396 | 0.03915 | * |
| R MV-re | sexF | 9.527e-07 | 6.2212e-07 | 1.5314 | 0.2326 |  |
| R MV-re | totalGray | -1.12E-11 | 5.1843e-12 | -2.1515 | 0.1433 |  |
| R MV-re | visit_age:groupDS | -1.73E-07 | 6.5795e-08 | -2.6244 | 0.028598 | * |
| R Pc | (Intercept) | 3.6509e-06 | 6.2398e-07 | 5.8511 | 3.0635e-08 | *** |
| R Pc | visit_age | -8.24E-09 | 8.037e-09 | -1.0254 | 0.9662 |  |
| R Pc | groupDS | 4.7408e-07 | 3.9741e-07 | 1.1929 | 0.28524 |  |
| R Pc | sexF | 1.9993e-07 | 8.4251e-08 | 2.373 | 0.08323 |  |
| R Pc | totalGray | -6.02E-13 | 7.0208e-13 | -0.85713 | 0.50269 |  |
| R Pc | visit_age:groupDS | -1.35E-08 | 8.9104e-09 | -1.5132 | 0.17427 |  |
| R Pf | (Intercept) | 3.642e-05 | 9.3933e-06 | 3.8772 | 0.000156 | *** |
| R Pf | visit_age | 1.3738e-07 | 1.2099e-07 | 1.1355 | 0.9662 |  |
| R Pf | groupDS | 2.1919e-05 | 5.9826e-06 | 3.6639 | 0.004378 | ** |
| R Pf | sexF | 1.7531e-06 | 1.2683e-06 | 1.3823 | 0.26247 |  |
| R Pf | totalGray | -6.11E-12 | 1.0569e-11 | -0.57778 | 0.64076 |  |
| R Pf | visit_age:groupDS | -3.88E-07 | 1.3414e-07 | -2.8953 | 0.026983 | * |
| R Pt | (Intercept) | 4.693e-06 | 9.9627e-07 | 4.7106 | 5.8733e-06 | *** |
| R Pt | visit_age | 1.3612e-08 | 1.2832e-08 | 1.0607 | 0.9662 |  |
| R Pt | groupDS | 1.8089e-06 | 6.3452e-07 | 2.8508 | 0.015238 | * |
| R Pt | sexF | 1.993e-07 | 1.3452e-07 | 1.4816 | 0.2326 |  |
| R Pt | totalGray | -7.10E-13 | 1.121e-12 | -0.6332 | 0.63799 |  |
| R Pt | visit_age:groupDS | -3.76E-08 | 1.4227e-08 | -2.643 | 0.028598 | * |
| R PuA | (Intercept) | 0.000165 | 2.4817e-05 | 6.66 | 4.4439e-10 | *** |
| R PuA | visit_age | -2.13E-08 | 3.1965e-07 | -0.06669 | 0.9662 |  |
| R PuA | groupDS | 5.4719e-05 | 1.5806e-05 | 3.4619 | 0.004423 | ** |
| R PuA | sexF | 6.5369e-06 | 3.3509e-06 | 1.9508 | 0.11425 |  |
| R PuA | totalGray | -1.29E-11 | 2.7923e-11 | -0.46349 | 0.67273 |  |
| R PuA | visit_age:groupDS | -1.17E-06 | 3.5439e-07 | -3.2982 | 0.018293 | * |
| R PuI | (Intercept) | 0.00026 | 3.4144e-05 | 7.605 | 2.3501e-12 | *** |
| R PuI | visit_age | -1.13E-06 | 4.3978e-07 | -2.578 | 0.5222 |  |
| R PuI | groupDS | 5.0349e-05 | 2.1746e-05 | 2.3153 | 0.040986 | * |
| R PuI | sexF | 8.9867e-06 | 4.6102e-06 | 1.9493 | 0.11425 |  |
| R PuI | totalGray | -6.92E-11 | 3.8418e-11 | -1.8018 | 0.17925 |  |
| R PuI | visit_age:groupDS | -7.58E-07 | 4.8757e-07 | -1.5549 | 0.16817 |  |
| R PuL | (Intercept) | 0.000217 | 2.5315e-05 | 8.5871 | 1.5157e-14 | *** |
| R PuL | visit_age | -2.43E-07 | 3.2606e-07 | -0.74547 | 0.9662 |  |
| R PuL | groupDS | 2.3236e-05 | 1.6123e-05 | 1.4412 | 0.20356 |  |
| R PuL | sexF | -2.05E-06 | 3.4181e-06 | -0.6008 | 0.6094 |  |
| R PuL | totalGray | -1.26E-10 | 2.8484e-11 | -4.4148 | 0.00072 | *** |
| R PuL | visit_age:groupDS | -6.30E-07 | 3.615e-07 | -1.7431 | 0.12875 |  |
| R PuM | (Intercept) | 0.000928 | 0.000125 | 7.3991 | 7.1275e-12 | *** |
| R PuM | visit_age | -1.33E-06 | 1.6161e-06 | -0.82061 | 0.9662 |  |
| R PuM | groupDS | 0.000289 | 7.991e-05 | 3.619 | 0.004378 | ** |
| R PuM | sexF | 3.4852e-05 | 1.6941e-05 | 2.0573 | 0.11425 |  |
| R PuM | totalGray | -1.38E-10 | 1.4117e-10 | -0.97584 | 0.47139 |  |
| R PuM | visit_age:groupDS | -5.45E-06 | 1.7917e-06 | -3.044 | 0.02554 | * |
| R VA | (Intercept) | 0.000293 | 4.0898e-05 | 7.161 | 2.567e-11 | *** |
| R VA | visit_age | -1.06E-07 | 5.2678e-07 | -0.20208 | 0.9662 |  |
| R VA | groupDS | 6.1211e-05 | 2.6048e-05 | 2.3499 | 0.040552 | * |
| R VA | sexF | 1.4778e-05 | 5.5222e-06 | 2.6761 | 0.056302 |  |
| R VA | totalGray | -2.12E-11 | 4.6018e-11 | -0.46007 | 0.67273 |  |
| R VA | visit_age:groupDS | -1.57E-06 | 5.8403e-07 | -2.6848 | 0.028598 | * |
| R VAmc | (Intercept) | 3.1821e-05 | 4.0439e-06 | 7.8688 | 5.4535e-13 | *** |
| R VAmc | visit_age | -2.80E-08 | 5.2086e-08 | -0.53692 | 0.9662 |  |
| R VAmc | groupDS | 6.6117e-06 | 2.5756e-06 | 2.5671 | 0.028928 | * |
| R VAmc | sexF | 1.0766e-06 | 5.4602e-07 | 1.9717 | 0.11425 |  |
| R VAmc | totalGray | -1.22E-11 | 4.5501e-12 | -2.6823 | 0.126 |  |
| R VAmc | visit_age:groupDS | -1.61E-07 | 5.7747e-08 | -2.7884 | 0.027108 | * |
| R VLa | (Intercept) | 0.000463 | 5.6841e-05 | 8.1497 | 1.0314e-13 | *** |
| R VLa | visit_age | 1.9407e-08 | 7.3212e-07 | 0.026508 | 0.97887 |  |
| R VLa | groupDS | 3.6203e-05 | 3.6202e-05 | 1 | 0.37875 |  |
| R VLa | sexF | 1.6215e-05 | 7.6748e-06 | 2.1127 | 0.11425 |  |
| R VLa | totalGray | -8.23E-11 | 6.3956e-11 | -1.2863 | 0.35604 |  |
| R VLa | visit_age:groupDS | -1.43E-06 | 8.1169e-07 | -1.7575 | 0.12875 |  |
| R VLp | (Intercept) | 0.000612 | 7.5151e-05 | 8.1434 | 1.0314e-13 | *** |
| R VLp | visit_age | 4.171e-07 | 9.6796e-07 | 0.43091 | 0.9662 |  |
| R VLp | groupDS | 4.6716e-05 | 4.7863e-05 | 0.97604 | 0.38358 |  |
| R VLp | sexF | 1.5227e-05 | 1.0147e-05 | 1.5006 | 0.2326 |  |
| R VLp | totalGray | -1.49E-10 | 8.4558e-11 | -1.7598 | 0.17925 |  |
| R VLp | visit_age:groupDS | -1.94E-06 | 1.0732e-06 | -1.8061 | 0.11993 |  |
| R VM | (Intercept) | 1.7581e-05 | 2.7097e-06 | 6.4881 | 1.0193e-09 | *** |
| R VM | visit_age | 4.8196e-08 | 3.4901e-08 | 1.3809 | 0.9662 |  |
| R VM | groupDS | 3.7924e-06 | 1.7258e-06 | 2.1975 | 0.053329 |  |
| R VM | sexF | -7.31E-08 | 3.6587e-07 | -0.19979 | 0.88886 |  |
| R VM | totalGray | -6.92E-12 | 3.0489e-12 | -2.2712 | 0.1433 |  |
| R VM | visit_age:groupDS | -8.49E-08 | 3.8694e-08 | -2.1943 | 0.058053 |  |
| R VPL | (Intercept) | 0.000655 | 8.6468e-05 | 7.5783 | 2.5092e-12 | *** |
| R VPL | visit_age | 1.3879e-06 | 1.1137e-06 | 1.2462 | 0.9662 |  |
| R VPL | groupDS | 0.000117 | 5.5071e-05 | 2.1257 | 0.061412 |  |
| R VPL | sexF | 2.1608e-06 | 1.1675e-05 | 0.18508 | 0.88886 |  |
| R VPL | totalGray | -1.86E-10 | 9.7292e-11 | -1.9135 | 0.17925 |  |
| R VPL | visit_age:groupDS | -2.98E-06 | 1.2348e-06 | -2.4173 | 0.040675 | * |
