# Supplementary material for: Structural MRI across lifespan reveals differential thalamic trajectories in Down syndrome
Source: Alzheimers Dement. 2026 Jul 14;22(7):e71671. doi: 10.1002/alz.71671 (PMC13369009; doi:10.1002/alz.71671)
Supplement: Supplementary file 7 — Supporting Information [file ALZ-22-e71671-s002.docx]

Table S5: GLM statistics and coefficients for biomarker analyses with respect to nuclei.

| Nucleus | Term | Estimate | Standard Error | T statistic | P-value | Residual degrees of freedom | T squared | Partial R squared | Cohen's f squared | Partial R squared (signed) | Significance | FDR-corrected p-value | FDR-corrected significance |
| --- | --- | --- | --- | --- | --- | --- | --- | --- | --- | --- | --- | --- | --- |
| R Pt | (Intercept) | 5.46E-06 | 3.62E-07 | 15.10197 | 2.26E-34 | 190 | 228.0696 | 0.54553 | 1.200366 | 0.54553 | *** | 4.11E-34 | *** |
| R Pt | Abeta40_Abeta42_ratio | -1.39E-08 | 1.88E-08 | -0.74363 | 0.458016164 | 190 | 0.552992 | 0.002902 | 0.00291 | -0.0029 | ns | 0.661578903 | ns |
| L VAmc | (Intercept) | 2.44E-05 | 1.61E-06 | 15.09505 | 2.37E-34 | 190 | 227.8606 | 0.545303 | 1.199266 | 0.545303 | *** | 4.11E-34 | *** |
| L VAmc | Abeta40_Abeta42_ratio | -7.52E-08 | 8.36E-08 | -0.89864 | 0.369981045 | 190 | 0.807557 | 0.004232 | 0.00425 | -0.00423 | ns | 0.613986184 | ns |
| L MV-re | (Intercept) | 1.24E-05 | 1.63E-06 | 7.614072 | 1.21E-12 | 190 | 57.97409 | 0.233791 | 0.305127 | 0.233791 | *** | 1.21E-12 | *** |
| L MV-re | Abeta40_Abeta42_ratio | -9.78E-08 | 8.46E-08 | -1.1552 | 0.249460119 | 190 | 1.334482 | 0.006975 | 0.007024 | -0.00697 | ns | 0.613986184 | ns |
| R VAmc | (Intercept) | 2.61E-05 | 1.58E-06 | 16.49257 | 1.64E-38 | 190 | 272.005 | 0.588749 | 1.431605 | 0.588749 | *** | 4.75E-38 | *** |
| R VAmc | Abeta40_Abeta42_ratio | -1.01E-07 | 8.21E-08 | -1.23409 | 0.218693388 | 190 | 1.52298 | 0.007952 | 0.008016 | -0.00795 | ns | 0.613986184 | ns |
| R MV-re | (Intercept) | 1.43E-05 | 1.72E-06 | 8.290155 | 2.02E-14 | 190 | 68.72668 | 0.265634 | 0.361719 | 0.265634 | *** | 2.10E-14 | *** |
| R MV-re | Abeta40_Abeta42_ratio | -1.42E-07 | 8.93E-08 | -1.59309 | 0.112801236 | 190 | 2.53795 | 0.013182 | 0.013358 | -0.01318 | ns | 0.613986184 | ns |
| R LD | (Intercept) | 2.02E-05 | 2.39E-06 | 8.446315 | 7.68E-15 | 190 | 71.34024 | 0.272978 | 0.375475 | 0.272978 | *** | 8.31E-15 | *** |
| R LD | Abeta40_Abeta42_ratio | -4.54E-08 | 1.24E-07 | -0.36665 | 0.714288403 | 190 | 0.134432 | 0.000707 | 0.000708 | -0.00071 | ns | 0.742859939 | ns |
| R Pf | (Intercept) | 4.90E-05 | 3.38E-06 | 14.49732 | 1.47E-32 | 190 | 210.1723 | 0.525204 | 1.10617 | 0.525204 | *** | 2.39E-32 | *** |
| R Pf | Abeta40_Abeta42_ratio | -2.12E-07 | 1.75E-07 | -1.21057 | 0.227562946 | 190 | 1.465486 | 0.007654 | 0.007713 | -0.00765 | ns | 0.613986184 | ns |
| L AV | (Intercept) | 9.61E-05 | 6.15E-06 | 15.6118 | 6.78E-36 | 190 | 243.7285 | 0.561938 | 1.282781 | 0.561938 | *** | 1.47E-35 | *** |
| L AV | Abeta40_Abeta42_ratio | -3.36E-07 | 3.19E-07 | -1.05198 | 0.294146616 | 190 | 1.106654 | 0.005791 | 0.005824 | -0.00579 | ns | 0.613986184 | ns |
| R CeM | (Intercept) | 6.27E-05 | 5.43E-06 | 11.5298 | 1.17E-23 | 190 | 132.9364 | 0.411649 | 0.699665 | 0.411649 | *** | 1.38E-23 | *** |
| R CeM | Abeta40_Abeta42_ratio | -4.48E-07 | 2.82E-07 | -1.59086 | 0.11330421 | 190 | 2.530826 | 0.013145 | 0.01332 | -0.01315 | ns | 0.613986184 | ns |
| L CeM | (Intercept) | 5.80E-05 | 5.77E-06 | 10.05236 | 2.47E-19 | 190 | 101.05 | 0.347191 | 0.531842 | 0.347191 | *** | 2.79E-19 | *** |
| L CeM | Abeta40_Abeta42_ratio | -3.15E-07 | 2.99E-07 | -1.0515 | 0.294365127 | 190 | 1.105649 | 0.005786 | 0.005819 | -0.00579 | ns | 0.613986184 | ns |
| L MGN | (Intercept) | 8.57E-05 | 6.22E-06 | 13.78491 | 2.04E-30 | 190 | 190.0239 | 0.500031 | 1.000126 | 0.500031 | *** | 2.65E-30 | *** |
| L MGN | Abeta40_Abeta42_ratio | 1.54E-07 | 3.22E-07 | 0.47678 | 0.634066856 | 190 | 0.227319 | 0.001195 | 0.001196 | 0.001195 | ns | 0.733382981 | ns |
| R MGN | (Intercept) | 9.69E-05 | 7.37E-06 | 13.13684 | 1.82E-28 | 190 | 172.5765 | 0.475973 | 0.908297 | 0.475973 | *** | 2.25E-28 | *** |
| R MGN | Abeta40_Abeta42_ratio | -7.70E-09 | 3.82E-07 | -0.02015 | 0.983946208 | 190 | 0.000406 | 2.14E-06 | 2.14E-06 | -2.14E-06 | ns | 0.983946208 | ns |
| R MDl | (Intercept) | 0.000201 | 1.18E-05 | 17.04187 | 3.95E-40 | 190 | 290.4254 | 0.604517 | 1.528555 | 0.604517 | *** | 1.47E-39 | *** |
| R MDl | Abeta40_Abeta42_ratio | -5.15E-07 | 6.13E-07 | -0.84092 | 0.401452505 | 190 | 0.707138 | 0.003708 | 0.003722 | -0.00371 | ns | 0.613986184 | ns |
| R PuA | (Intercept) | 0.000174 | 9.28E-06 | 18.73721 | 4.74E-45 | 190 | 351.0831 | 0.648852 | 1.847806 | 0.648852 | *** | 4.11E-44 | *** |
| R PuA | Abeta40_Abeta42_ratio | -4.77E-07 | 4.81E-07 | -0.99143 | 0.322735516 | 190 | 0.982938 | 0.005147 | 0.005173 | -0.00515 | ns | 0.613986184 | ns |
| R CM | (Intercept) | 0.000196 | 1.12E-05 | 17.45263 | 2.48E-41 | 190 | 304.5945 | 0.615847 | 1.603129 | 0.615847 | *** | 1.07E-40 | *** |
| R CM | Abeta40_Abeta42_ratio | -6.95E-07 | 5.82E-07 | -1.19378 | 0.234053744 | 190 | 1.425102 | 0.007445 | 0.007501 | -0.00744 | ns | 0.613986184 | ns |
| L LGN | (Intercept) | 0.0002 | 1.42E-05 | 14.08518 | 2.55E-31 | 190 | 198.3924 | 0.510804 | 1.04417 | 0.510804 | *** | 3.49E-31 | *** |
| L LGN | Abeta40_Abeta42_ratio | -3.36E-07 | 7.37E-07 | -0.45621 | 0.648761868 | 190 | 0.208125 | 0.001094 | 0.001095 | -0.00109 | ns | 0.733382981 | ns |
| L MDl | (Intercept) | 0.000194 | 1.22E-05 | 15.91908 | 8.25E-37 | 190 | 253.417 | 0.571509 | 1.333774 | 0.571509 | *** | 1.95E-36 | *** |
| L MDl | Abeta40_Abeta42_ratio | -3.46E-07 | 6.31E-07 | -0.54735 | 0.584777726 | 190 | 0.299597 | 0.001574 | 0.001577 | -0.00157 | ns | 0.733382981 | ns |
| L PuA | (Intercept) | 0.000171 | 1.19E-05 | 14.39669 | 2.95E-32 | 190 | 207.2645 | 0.521729 | 1.090866 | 0.521729 | *** | 4.52E-32 | *** |
| L PuA | Abeta40_Abeta42_ratio | -3.30E-07 | 6.16E-07 | -0.53558 | 0.592874962 | 190 | 0.286846 | 0.001507 | 0.00151 | -0.00151 | ns | 0.733382981 | ns |
| R VA | (Intercept) | 0.000305 | 1.53E-05 | 19.91523 | 2.16E-48 | 190 | 396.6165 | 0.676109 | 2.087455 | 0.676109 | *** | 2.80E-47 | *** |
| R VA | Abeta40_Abeta42_ratio | -1.48E-06 | 7.95E-07 | -1.86254 | 0.064071175 | 190 | 3.469045 | 0.017931 | 0.018258 | -0.01793 | ns | 0.613986184 | ns |
| R LGN | (Intercept) | 0.000203 | 1.42E-05 | 14.36202 | 3.75E-32 | 190 | 206.2677 | 0.520526 | 1.085619 | 0.520526 | *** | 5.42E-32 | *** |
| R LGN | Abeta40_Abeta42_ratio | -1.01E-06 | 7.34E-07 | -1.37158 | 0.171811019 | 190 | 1.881238 | 0.009804 | 0.009901 | -0.0098 | ns | 0.613986184 | ns |
| L VA | (Intercept) | 0.000303 | 1.87E-05 | 16.19074 | 1.29E-37 | 190 | 262.1402 | 0.579776 | 1.379685 | 0.579776 | *** | 3.34E-37 | *** |
| L VA | Abeta40_Abeta42_ratio | -1.29E-06 | 9.69E-07 | -1.33344 | 0.183983129 | 190 | 1.778068 | 0.009271 | 0.009358 | -0.00927 | ns | 0.613986184 | ns |
| R VPL | (Intercept) | 0.000653 | 3.01E-05 | 21.71391 | 2.32E-53 | 190 | 471.4937 | 0.712771 | 2.481546 | 0.712771 | *** | 6.04E-52 | *** |
| R VPL | Abeta40_Abeta42_ratio | -2.47E-06 | 1.56E-06 | -1.58428 | 0.114791944 | 190 | 2.509958 | 0.013038 | 0.01321 | -0.01304 | ns | 0.613986184 | ns |
| R MDm | (Intercept) | 0.000592 | 3.22E-05 | 18.405 | 4.27E-44 | 190 | 338.7441 | 0.640658 | 1.782864 | 0.640658 | *** | 2.22E-43 | *** |
| R MDm | Abeta40_Abeta42_ratio | -1.58E-06 | 1.67E-06 | -0.94471 | 0.346008205 | 190 | 0.892472 | 0.004675 | 0.004697 | -0.00468 | ns | 0.613986184 | ns |
| L MDm | (Intercept) | 0.000587 | 3.51E-05 | 16.73226 | 3.22E-39 | 190 | 279.9686 | 0.595718 | 1.473519 | 0.595718 | *** | 1.05E-38 | *** |
| L MDm | Abeta40_Abeta42_ratio | -1.21E-06 | 1.82E-06 | -0.66665 | 0.505806941 | 190 | 0.444417 | 0.002334 | 0.002339 | -0.00233 | ns | 0.692156867 | ns |
| R PuM | (Intercept) | 0.000907 | 4.88E-05 | 18.56984 | 1.43E-44 | 190 | 344.839 | 0.644753 | 1.814942 | 0.644753 | *** | 9.32E-44 | *** |
| R PuM | Abeta40_Abeta42_ratio | -2.19E-06 | 2.53E-06 | -0.86387 | 0.388749066 | 190 | 0.74627 | 0.003912 | 0.003928 | -0.00391 | ns | 0.613986184 | ns |
| L PuM | (Intercept) | 0.00088 | 5.72E-05 | 15.3868 | 3.18E-35 | 190 | 236.7535 | 0.554778 | 1.246071 | 0.554778 | *** | 6.37E-35 | *** |
| L PuM | Abeta40_Abeta42_ratio | -1.12E-06 | 2.96E-06 | -0.37675 | 0.706780475 | 190 | 0.14194 | 0.000746 | 0.000747 | -0.00075 | ns | 0.742859939 | ns |
| R Pt | (Intercept) | 4.89E-06 | 3.35E-07 | 14.60442 | 5.61E-33 | 192 | 213.2892 | 0.526264 | 1.110881 | 0.526264 | *** | 1.04E-32 | *** |
| R Pt | Abeta40 | 9.37E-10 | 1.02E-09 | 0.921755 | 0.357813398 | 192 | 0.849631 | 0.004406 | 0.004425 | 0.004406 | ns | 0.715626797 | ns |
| L VAmc | (Intercept) | 2.13E-05 | 1.50E-06 | 14.17588 | 1.10E-31 | 192 | 200.9557 | 0.511395 | 1.046644 | 0.511395 | *** | 1.79E-31 | *** |
| L VAmc | Abeta40 | 5.21E-09 | 4.56E-09 | 1.143885 | 0.25409526 | 192 | 1.308474 | 0.006769 | 0.006815 | 0.006769 | ns | 0.633752428 | ns |
| L MV-re | (Intercept) | 1.10E-05 | 1.53E-06 | 7.177113 | 1.51E-11 | 192 | 51.51095 | 0.211534 | 0.268286 | 0.211534 | *** | 1.51E-11 | *** |
| L MV-re | Abeta40 | -9.81E-10 | 4.64E-09 | -0.2116 | 0.832642847 | 192 | 0.044775 | 0.000233 | 0.000233 | -0.00023 | ns | 0.902029751 | ns |
| R VAmc | (Intercept) | 2.32E-05 | 1.47E-06 | 15.77747 | 1.66E-36 | 192 | 248.9285 | 0.564555 | 1.296502 | 0.564555 | *** | 4.79E-36 | *** |
| R VAmc | Abeta40 | 3.16E-09 | 4.46E-09 | 0.707836 | 0.479905725 | 192 | 0.501031 | 0.002603 | 0.00261 | 0.002603 | ns | 0.755487123 | ns |
| R MV-re | (Intercept) | 1.21E-05 | 1.61E-06 | 7.525508 | 1.98E-12 | 192 | 56.63326 | 0.227778 | 0.294965 | 0.227778 | *** | 2.06E-12 | *** |
| R MV-re | Abeta40 | -1.42E-09 | 4.88E-09 | -0.2908 | 0.771519655 | 192 | 0.084564 | 0.00044 | 0.00044 | -0.00044 | ns | 0.902029751 | ns |
| R LD | (Intercept) | 1.77E-05 | 2.22E-06 | 7.999313 | 1.15E-13 | 192 | 63.98902 | 0.249968 | 0.333276 | 0.249968 | *** | 1.25E-13 | *** |
| R LD | Abeta40 | 4.76E-09 | 6.73E-09 | 0.70759 | 0.480057738 | 192 | 0.500684 | 0.002601 | 0.002608 | 0.002601 | ns | 0.755487123 | ns |
| R Pf | (Intercept) | 4.13E-05 | 3.14E-06 | 13.16918 | 1.22E-28 | 192 | 173.4274 | 0.474588 | 0.903268 | 0.474588 | *** | 1.59E-28 | *** |
| R Pf | Abeta40 | 1.15E-08 | 9.52E-09 | 1.213299 | 0.226506172 | 192 | 1.472094 | 0.007609 | 0.007667 | 0.007609 | ns | 0.633752428 | ns |
| L AV | (Intercept) | 8.14E-05 | 5.72E-06 | 14.23637 | 7.25E-32 | 192 | 202.6743 | 0.513523 | 1.055595 | 0.513523 | *** | 1.26E-31 | *** |
| L AV | Abeta40 | 2.55E-08 | 1.74E-08 | 1.47033 | 0.143109148 | 192 | 2.161871 | 0.011134 | 0.01126 | 0.011134 | ns | 0.633752428 | ns |
| R CeM | (Intercept) | 5.26E-05 | 5.07E-06 | 10.37088 | 2.71E-20 | 192 | 107.5551 | 0.35905 | 0.560183 | 0.35905 | *** | 3.21E-20 | *** |
| R CeM | Abeta40 | 5.17E-09 | 1.54E-08 | 0.335908 | 0.737307139 | 192 | 0.112834 | 0.000587 | 0.000588 | 0.000587 | ns | 0.902029751 | ns |
| L CeM | (Intercept) | 4.90E-05 | 5.38E-06 | 9.112762 | 1.07E-16 | 192 | 83.04242 | 0.301926 | 0.432513 | 0.301926 | *** | 1.21E-16 | *** |
| L CeM | Abeta40 | 9.81E-09 | 1.63E-08 | 0.601021 | 0.548534745 | 192 | 0.361226 | 0.001878 | 0.001881 | 0.001878 | ns | 0.755487123 | ns |
| L MGN | (Intercept) | 8.81E-05 | 5.78E-06 | 15.23935 | 6.84E-35 | 192 | 232.2378 | 0.547424 | 1.209572 | 0.547424 | *** | 1.62E-34 | *** |
| L MGN | Abeta40 | 1.90E-09 | 1.75E-08 | 0.10849 | 0.91372056 | 192 | 0.01177 | 6.13E-05 | 6.13E-05 | 6.13E-05 | ns | 0.942277671 | ns |
| R MGN | (Intercept) | 9.36E-05 | 6.85E-06 | 13.66888 | 3.77E-30 | 192 | 186.8384 | 0.493188 | 0.973117 | 0.493188 | *** | 5.16E-30 | *** |
| R MGN | Abeta40 | 1.02E-08 | 2.08E-08 | 0.491746 | 0.62346089 | 192 | 0.241814 | 0.001258 | 0.001259 | 0.001258 | ns | 0.810499157 | ns |
| R MDl | (Intercept) | 0.000186 | 1.09E-05 | 16.9549 | 5.17E-40 | 192 | 287.4686 | 0.599557 | 1.497232 | 0.599557 | *** | 2.24E-39 | *** |
| R MDl | Abeta40 | 1.98E-08 | 3.32E-08 | 0.595686 | 0.552086744 | 192 | 0.354841 | 0.001845 | 0.001848 | 0.001845 | ns | 0.755487123 | ns |
| R PuA | (Intercept) | 0.000159 | 8.60E-06 | 18.52126 | 1.34E-44 | 192 | 343.0372 | 0.641146 | 1.786652 | 0.641146 | *** | 8.71E-44 | *** |
| R PuA | Abeta40 | 1.76E-08 | 2.61E-08 | 0.676245 | 0.499698646 | 192 | 0.457308 | 0.002376 | 0.002382 | 0.002376 | ns | 0.755487123 | ns |
| R CM | (Intercept) | 0.000169 | 1.04E-05 | 16.26416 | 5.82E-38 | 192 | 264.5229 | 0.57943 | 1.377724 | 0.57943 | *** | 2.16E-37 | *** |
| R CM | Abeta40 | 4.27E-08 | 3.15E-08 | 1.354173 | 0.177272775 | 192 | 1.833785 | 0.009461 | 0.009551 | 0.009461 | ns | 0.633752428 | ns |
| L LGN | (Intercept) | 0.000169 | 1.31E-05 | 12.91154 | 7.33E-28 | 192 | 166.7078 | 0.464745 | 0.86827 | 0.464745 | *** | 9.08E-28 | *** |
| L LGN | Abeta40 | 7.92E-08 | 3.96E-08 | 1.999657 | 0.046947052 | 192 | 3.998629 | 0.020401 | 0.020826 | 0.020401 | * | 0.633752428 | ns |
| L MDl | (Intercept) | 0.000176 | 1.13E-05 | 15.63538 | 4.42E-36 | 192 | 244.4652 | 0.560102 | 1.273256 | 0.560102 | *** | 1.15E-35 | *** |
| L MDl | Abeta40 | 3.65E-08 | 3.42E-08 | 1.06817 | 0.286784963 | 192 | 1.140987 | 0.005908 | 0.005943 | 0.005908 | ns | 0.633752428 | ns |
| L PuA | (Intercept) | 0.000153 | 1.10E-05 | 13.97031 | 4.62E-31 | 192 | 195.1695 | 0.504093 | 1.016508 | 0.504093 | *** | 6.67E-31 | *** |
| L PuA | Abeta40 | 3.55E-08 | 3.33E-08 | 1.065254 | 0.288098679 | 192 | 1.134767 | 0.005876 | 0.00591 | 0.005876 | ns | 0.633752428 | ns |
| R VA | (Intercept) | 0.000266 | 1.43E-05 | 18.58616 | 8.70E-45 | 192 | 345.4455 | 0.642754 | 1.799195 | 0.642754 | *** | 7.54E-44 | *** |
| R VA | Abeta40 | 3.48E-08 | 4.34E-08 | 0.80181 | 0.423654303 | 192 | 0.642899 | 0.003337 | 0.003348 | 0.003337 | ns | 0.755487123 | ns |
| R LGN | (Intercept) | 0.000186 | 1.32E-05 | 14.04692 | 2.71E-31 | 192 | 197.316 | 0.506827 | 1.027688 | 0.506827 | *** | 4.15E-31 | *** |
| R LGN | Abeta40 | -2.91E-09 | 4.01E-08 | -0.0725 | 0.942277671 | 192 | 0.005257 | 2.74E-05 | 2.74E-05 | -2.74E-05 | ns | 0.942277671 | ns |
| L VA | (Intercept) | 0.000261 | 1.75E-05 | 14.90515 | 6.94E-34 | 192 | 222.1635 | 0.536415 | 1.157102 | 0.536415 | *** | 1.50E-33 | *** |
| L VA | Abeta40 | 5.61E-08 | 5.31E-08 | 1.055548 | 0.292501121 | 192 | 1.114182 | 0.00577 | 0.005803 | 0.00577 | ns | 0.633752428 | ns |
| R VPL | (Intercept) | 0.000575 | 2.80E-05 | 20.52816 | 2.59E-50 | 192 | 421.4053 | 0.686993 | 2.194819 | 0.686993 | *** | 6.74E-49 | *** |
| R VPL | Abeta40 | 9.45E-08 | 8.50E-08 | 1.111654 | 0.267677147 | 192 | 1.235774 | 0.006395 | 0.006436 | 0.006395 | ns | 0.633752428 | ns |
| R MDm | (Intercept) | 0.000532 | 2.98E-05 | 17.83558 | 1.33E-42 | 192 | 318.108 | 0.623609 | 1.656812 | 0.623609 | *** | 6.89E-42 | *** |
| R MDm | Abeta40 | 9.64E-08 | 9.06E-08 | 1.065013 | 0.28820774 | 192 | 1.134252 | 0.005873 | 0.005908 | 0.005873 | ns | 0.633752428 | ns |
| L MDm | (Intercept) | 0.000523 | 3.24E-05 | 16.14864 | 1.29E-37 | 192 | 260.7786 | 0.575952 | 1.358222 | 0.575952 | *** | 4.18E-37 | *** |
| L MDm | Abeta40 | 1.32E-07 | 9.83E-08 | 1.337549 | 0.18262566 | 192 | 1.789038 | 0.009232 | 0.009318 | 0.009232 | ns | 0.633752428 | ns |
| R PuM | (Intercept) | 0.000854 | 4.53E-05 | 18.84562 | 1.55E-45 | 192 | 355.1573 | 0.649095 | 1.849778 | 0.649095 | *** | 2.02E-44 | *** |
| R PuM | Abeta40 | 3.52E-08 | 1.38E-07 | 0.256256 | 0.798027219 | 192 | 0.065667 | 0.000342 | 0.000342 | 0.000342 | ns | 0.902029751 | ns |
| L PuM | (Intercept) | 0.000789 | 5.31E-05 | 14.87069 | 8.82E-34 | 192 | 221.1373 | 0.535263 | 1.151757 | 0.535263 | *** | 1.76E-33 | *** |
| L PuM | Abeta40 | 2.15E-07 | 1.61E-07 | 1.333651 | 0.183898093 | 192 | 1.778626 | 0.009179 | 0.009264 | 0.009179 | ns | 0.633752428 | ns |
| R Pt | (Intercept) | 4.89E-06 | 2.85E-07 | 17.18233 | 7.93E-41 | 194 | 295.2323 | 0.60346 | 1.521816 | 0.60346 | *** | 1.47E-40 | *** |
| R Pt | Abeta42 | 1.82E-08 | 1.60E-08 | 1.136301 | 0.257232129 | 194 | 1.291181 | 0.006612 | 0.006656 | 0.006612 | ns | 0.483172697 | ns |
| L VAmc | (Intercept) | 2.14E-05 | 1.27E-06 | 16.8655 | 6.95E-40 | 194 | 284.4451 | 0.59452 | 1.466212 | 0.59452 | *** | 1.20E-39 | *** |
| L VAmc | Abeta42 | 8.91E-08 | 7.15E-08 | 1.246548 | 0.214066178 | 194 | 1.553883 | 0.007946 | 0.00801 | 0.007946 | ns | 0.483172697 | ns |
| L MV-re | (Intercept) | 1.04E-05 | 1.29E-06 | 8.008576 | 1.05E-13 | 194 | 64.13729 | 0.248462 | 0.330605 | 0.248462 | *** | 1.05E-13 | *** |
| L MV-re | Abeta42 | 1.58E-08 | 7.28E-08 | 0.216642 | 0.828714845 | 194 | 0.046934 | 0.000242 | 0.000242 | 0.000242 | ns | 0.861863438 | ns |
| R VAmc | (Intercept) | 2.28E-05 | 1.24E-06 | 18.31145 | 3.73E-44 | 194 | 335.3093 | 0.633485 | 1.728398 | 0.633485 | *** | 8.83E-44 | *** |
| R VAmc | Abeta42 | 8.42E-08 | 6.99E-08 | 1.20542 | 0.229509402 | 194 | 1.453038 | 0.007434 | 0.00749 | 0.007434 | ns | 0.483172697 | ns |
| R MV-re | (Intercept) | 1.11E-05 | 1.36E-06 | 8.105573 | 5.79E-14 | 194 | 65.70032 | 0.252985 | 0.338661 | 0.252985 | *** | 6.02E-14 | *** |
| R MV-re | Abeta42 | 3.29E-08 | 7.66E-08 | 0.429772 | 0.667838498 | 194 | 0.184704 | 0.000951 | 0.000952 | 0.000951 | ns | 0.723491706 | ns |
| R LD | (Intercept) | 1.79E-05 | 1.89E-06 | 9.503288 | 7.95E-18 | 194 | 90.31249 | 0.317652 | 0.465528 | 0.317652 | *** | 8.61E-18 | *** |
| R LD | Abeta42 | 8.21E-08 | 1.06E-07 | 0.773986 | 0.43988131 | 194 | 0.599054 | 0.003078 | 0.003088 | 0.003078 | ns | 0.635384115 | ns |
| R Pf | (Intercept) | 4.05E-05 | 2.65E-06 | 15.2908 | 3.74E-35 | 194 | 233.8084 | 0.546526 | 1.205198 | 0.546526 | *** | 4.63E-35 | *** |
| R Pf | Abeta42 | 2.63E-07 | 1.49E-07 | 1.766896 | 0.078818069 | 194 | 3.121921 | 0.015838 | 0.016092 | 0.015838 | ns | 0.381162728 | ns |
| L AV | (Intercept) | 8.07E-05 | 4.83E-06 | 16.69879 | 2.18E-39 | 194 | 278.8495 | 0.589721 | 1.437368 | 0.589721 | *** | 3.55E-39 | *** |
| L AV | Abeta42 | 5.27E-07 | 2.72E-07 | 1.942215 | 0.053558902 | 194 | 3.7722 | 0.019073 | 0.019444 | 0.019073 | ns | 0.381162728 | ns |
| R CeM | (Intercept) | 5.01E-05 | 4.29E-06 | 11.68222 | 3.21E-24 | 194 | 136.4742 | 0.412965 | 0.703475 | 0.412965 | *** | 3.80E-24 | *** |
| R CeM | Abeta42 | 2.37E-07 | 2.41E-07 | 0.983929 | 0.326375718 | 194 | 0.968117 | 0.004966 | 0.00499 | 0.004966 | ns | 0.518406923 | ns |
| L CeM | (Intercept) | 4.93E-05 | 4.55E-06 | 10.8265 | 1.14E-21 | 194 | 117.2132 | 0.376633 | 0.604191 | 0.376633 | *** | 1.29E-21 | *** |
| L CeM | Abeta42 | 1.68E-07 | 2.56E-07 | 0.65585 | 0.512697696 | 194 | 0.430139 | 0.002212 | 0.002217 | 0.002212 | ns | 0.642539708 | ns |
| L MGN | (Intercept) | 9.14E-05 | 4.89E-06 | 18.67753 | 3.20E-45 | 194 | 348.8502 | 0.642627 | 1.798197 | 0.642627 | *** | 9.24E-45 | *** |
| L MGN | Abeta42 | -1.56E-07 | 2.75E-07 | -0.56633 | 0.571824065 | 194 | 0.32073 | 0.001651 | 0.001653 | -0.00165 | ns | 0.675792077 | ns |
| R MGN | (Intercept) | 9.67E-05 | 5.81E-06 | 16.6519 | 3.02E-39 | 194 | 277.2858 | 0.58836 | 1.429308 | 0.58836 | *** | 4.61E-39 | *** |
| R MGN | Abeta42 | 9.22E-09 | 3.26E-07 | 0.028251 | 0.977491021 | 194 | 0.000798 | 4.11E-06 | 4.11E-06 | 4.11E-06 | ns | 0.977491021 | ns |
| R MDl | (Intercept) | 0.000186 | 9.29E-06 | 19.99105 | 5.34E-49 | 194 | 399.6419 | 0.673204 | 2.06001 | 0.673204 | *** | 2.31E-48 | *** |
| R MDl | Abeta42 | 3.55E-07 | 5.22E-07 | 0.678636 | 0.498177176 | 194 | 0.460547 | 0.002368 | 0.002374 | 0.002368 | ns | 0.642539708 | ns |
| R PuA | (Intercept) | 0.000157 | 7.32E-06 | 21.49063 | 3.31E-53 | 194 | 461.8471 | 0.704199 | 2.380655 | 0.704199 | *** | 2.87E-52 | *** |
| R PuA | Abeta42 | 4.52E-07 | 4.11E-07 | 1.098412 | 0.273385948 | 194 | 1.206509 | 0.006181 | 0.006219 | 0.006181 | ns | 0.483172697 | ns |
| R CM | (Intercept) | 0.000167 | 8.79E-06 | 19.04534 | 2.75E-46 | 194 | 362.725 | 0.651534 | 1.869716 | 0.651534 | *** | 8.93E-46 | *** |
| R CM | Abeta42 | 9.08E-07 | 4.94E-07 | 1.83666 | 0.067790233 | 194 | 3.37332 | 0.017091 | 0.017388 | 0.017091 | ns | 0.381162728 | ns |
| L LGN | (Intercept) | 0.000175 | 1.11E-05 | 15.7828 | 1.22E-36 | 194 | 249.0967 | 0.562172 | 1.284004 | 0.562172 | *** | 1.59E-36 | *** |
| L LGN | Abeta42 | 1.08E-06 | 6.24E-07 | 1.728651 | 0.085462599 | 194 | 2.988233 | 0.01517 | 0.015403 | 0.01517 | ns | 0.381162728 | ns |
| L MDl | (Intercept) | 0.000179 | 9.61E-06 | 18.63587 | 4.23E-45 | 194 | 347.2957 | 0.641601 | 1.790184 | 0.641601 | *** | 1.10E-44 | *** |
| L MDl | Abeta42 | 5.18E-07 | 5.40E-07 | 0.95859 | 0.338958373 | 194 | 0.918895 | 0.004714 | 0.004737 | 0.004714 | ns | 0.518406923 | ns |
| L PuA | (Intercept) | 0.000153 | 9.31E-06 | 16.44929 | 1.22E-38 | 194 | 270.5793 | 0.582418 | 1.394739 | 0.582418 | *** | 1.76E-38 | *** |
| L PuA | Abeta42 | 6.85E-07 | 5.23E-07 | 1.308272 | 0.192329047 | 194 | 1.711575 | 0.008745 | 0.008823 | 0.008745 | ns | 0.483172697 | ns |
| R VA | (Intercept) | 0.000257 | 1.21E-05 | 21.32593 | 9.46E-53 | 194 | 454.7953 | 0.700984 | 2.344306 | 0.700984 | *** | 6.15E-52 | *** |
| R VA | Abeta42 | 1.16E-06 | 6.78E-07 | 1.714899 | 0.08796063 | 194 | 2.940877 | 0.014933 | 0.015159 | 0.014933 | ns | 0.381162728 | ns |
| R LGN | (Intercept) | 0.000179 | 1.12E-05 | 16.00814 | 2.56E-37 | 194 | 256.2606 | 0.569138 | 1.320931 | 0.569138 | *** | 3.51E-37 | *** |
| R LGN | Abeta42 | 3.30E-07 | 6.28E-07 | 0.524355 | 0.600630036 | 194 | 0.274948 | 0.001415 | 0.001417 | 0.001415 | ns | 0.678973084 | ns |
| L VA | (Intercept) | 0.000263 | 1.49E-05 | 17.65491 | 3.17E-42 | 194 | 311.6959 | 0.61637 | 1.60668 | 0.61637 | *** | 6.33E-42 | *** |
| L VA | Abeta42 | 9.77E-07 | 8.36E-07 | 1.169219 | 0.243750103 | 194 | 1.367072 | 0.006997 | 0.007047 | 0.006997 | ns | 0.483172697 | ns |
| R VPL | (Intercept) | 0.000555 | 2.36E-05 | 23.52412 | 1.01E-58 | 194 | 553.3842 | 0.740428 | 2.852496 | 0.740428 | *** | 2.63E-57 | *** |
| R VPL | Abeta42 | 2.96E-06 | 1.33E-06 | 2.233469 | 0.026660257 | 194 | 4.988385 | 0.025069 | 0.025713 | 0.025069 | * | 0.381162728 | ns |
| R MDm | (Intercept) | 0.000537 | 2.53E-05 | 21.20936 | 1.99E-52 | 194 | 449.8369 | 0.698681 | 2.318747 | 0.698681 | *** | 1.04E-51 | *** |
| R MDm | Abeta42 | 1.55E-06 | 1.42E-06 | 1.086166 | 0.278753479 | 194 | 1.179756 | 0.006044 | 0.006081 | 0.006044 | ns | 0.483172697 | ns |
| L MDm | (Intercept) | 0.000531 | 2.75E-05 | 19.3081 | 4.80E-47 | 194 | 372.8029 | 0.657729 | 1.921664 | 0.657729 | *** | 1.78E-46 | *** |
| L MDm | Abeta42 | 1.96E-06 | 1.55E-06 | 1.2691 | 0.205925739 | 194 | 1.610616 | 0.008234 | 0.008302 | 0.008234 | ns | 0.483172697 | ns |
| R PuM | (Intercept) | 0.000842 | 3.85E-05 | 21.86761 | 3.02E-54 | 194 | 478.1924 | 0.711392 | 2.464909 | 0.711392 | *** | 3.93E-53 | *** |
| R PuM | Abeta42 | 1.40E-06 | 2.16E-06 | 0.646106 | 0.518974379 | 194 | 0.417453 | 0.002147 | 0.002152 | 0.002147 | ns | 0.642539708 | ns |
| L PuM | (Intercept) | 0.000802 | 4.50E-05 | 17.83973 | 9.04E-43 | 194 | 318.2558 | 0.621283 | 1.640494 | 0.621283 | *** | 1.96E-42 | *** |
| L PuM | Abeta42 | 3.26E-06 | 2.53E-06 | 1.290559 | 0.198392728 | 194 | 1.665542 | 0.008512 | 0.008585 | 0.008512 | ns | 0.483172697 | ns |
| R Pt | (Intercept) | 5.16E-06 | 9.88E-08 | 52.26754 | 2.58E-114 | 189 | 2731.895 | 0.935294 | 14.45447 | 0.935294 | *** | 4.79E-114 | *** |
| R Pt | CRP | 8.32E-15 | 1.06E-14 | 0.781086 | 0.435729147 | 189 | 0.610095 | 0.003218 | 0.003228 | 0.003218 | ns | 0.539474182 | ns |
| L VAmc | (Intercept) | 2.22E-05 | 4.26E-07 | 52.15645 | 3.76E-114 | 189 | 2720.295 | 0.935036 | 14.3931 | 0.935036 | *** | 6.52E-114 | *** |
| L VAmc | CRP | 1.05E-13 | 4.59E-14 | 2.287288 | 0.0232868 | 189 | 5.231686 | 0.026935 | 0.027681 | 0.026935 | * | 0.067272979 | ns |
| L MV-re | (Intercept) | 9.76E-06 | 4.22E-07 | 23.13605 | 4.93E-57 | 189 | 535.277 | 0.73905 | 2.832153 | 0.73905 | *** | 4.93E-57 | *** |
| L MV-re | CRP | 1.26E-13 | 4.55E-14 | 2.770841 | 0.006149718 | 189 | 7.677558 | 0.039036 | 0.040622 | 0.039036 | ** | 0.026648777 | * |
| R VAmc | (Intercept) | 2.34E-05 | 4.20E-07 | 55.64359 | 3.86E-119 | 189 | 3096.209 | 0.942469 | 16.38206 | 0.942469 | *** | 1.00E-118 | *** |
| R VAmc | CRP | 1.30E-13 | 4.53E-14 | 2.876899 | 0.004477799 | 189 | 8.27655 | 0.041954 | 0.043791 | 0.041954 | ** | 0.023284553 | * |
| R MV-re | (Intercept) | 1.06E-05 | 4.46E-07 | 23.75749 | 1.18E-58 | 189 | 564.4181 | 0.749143 | 2.986339 | 0.749143 | *** | 1.22E-58 | *** |
| R MV-re | CRP | 1.58E-13 | 4.80E-14 | 3.285639 | 0.001212787 | 189 | 10.79542 | 0.054032 | 0.057119 | 0.054032 | ** | 0.013849543 | * |
| R LD | (Intercept) | 1.90E-05 | 6.47E-07 | 29.40133 | 1.97E-72 | 189 | 864.4384 | 0.820588 | 4.573748 | 0.820588 | *** | 2.14E-72 | *** |
| R LD | CRP | 3.64E-14 | 6.98E-14 | 0.521489 | 0.602636637 | 189 | 0.271951 | 0.001437 | 0.001439 | 0.001437 | ns | 0.681241415 | ns |
| R Pf | (Intercept) | 4.35E-05 | 9.05E-07 | 48.0902 | 5.93E-108 | 189 | 2312.667 | 0.92445 | 12.23633 | 0.92445 | *** | 8.11E-108 | *** |
| R Pf | CRP | 2.56E-13 | 9.75E-14 | 2.623021 | 0.009426605 | 189 | 6.880237 | 0.035125 | 0.036403 | 0.035125 | ** | 0.035013104 | * |
| L AV | (Intercept) | 8.63E-05 | 1.60E-06 | 53.99096 | 8.23E-117 | 189 | 2915.023 | 0.939111 | 15.4234 | 0.939111 | *** | 1.65E-116 | *** |
| L AV | CRP | 5.26E-13 | 1.72E-13 | 3.050997 | 0.002608766 | 189 | 9.308583 | 0.04694 | 0.049252 | 0.04694 | ** | 0.016956976 | * |
| R CeM | (Intercept) | 5.09E-05 | 1.40E-06 | 36.30286 | 3.88E-87 | 189 | 1317.898 | 0.874577 | 6.973004 | 0.874577 | *** | 4.58E-87 | *** |
| R CeM | CRP | 5.00E-13 | 1.51E-13 | 3.309007 | 0.001121071 | 189 | 10.94953 | 0.054761 | 0.057934 | 0.054761 | ** | 0.013849543 | * |
| L CeM | (Intercept) | 4.87E-05 | 1.46E-06 | 33.34304 | 4.27E-81 | 189 | 1111.758 | 0.8547 | 5.882318 | 0.8547 | *** | 4.83E-81 | *** |
| L CeM | CRP | 5.05E-13 | 1.58E-13 | 3.202645 | 0.001598024 | 189 | 10.25694 | 0.051476 | 0.05427 | 0.051476 | ** | 0.013849543 | * |
| L MGN | (Intercept) | 8.69E-05 | 1.70E-06 | 51.12408 | 1.28E-112 | 189 | 2613.672 | 0.932564 | 13.82895 | 0.932564 | *** | 2.08E-112 | *** |
| L MGN | CRP | 2.62E-13 | 1.83E-13 | 1.430758 | 0.154150823 | 189 | 2.047069 | 0.010715 | 0.010831 | 0.010715 | ns | 0.235760082 | ns |
| R MGN | (Intercept) | 9.51E-05 | 2.01E-06 | 47.3558 | 8.71E-107 | 189 | 2242.571 | 0.922272 | 11.86546 | 0.922272 | *** | 1.08E-106 | *** |
| R MGN | CRP | 2.47E-13 | 2.16E-13 | 1.141797 | 0.254982857 | 189 | 1.303701 | 0.006851 | 0.006898 | 0.006851 | ns | 0.368308571 | ns |
| R MDl | (Intercept) | 0.000187 | 3.19E-06 | 58.71514 | 2.61E-123 | 189 | 3447.468 | 0.948026 | 18.24057 | 0.948026 | *** | 9.68E-123 | *** |
| R MDl | CRP | 8.41E-13 | 3.43E-13 | 2.450578 | 0.015172533 | 189 | 6.005334 | 0.030796 | 0.031774 | 0.030796 | * | 0.049310732 | * |
| R PuA | (Intercept) | 0.000162 | 2.52E-06 | 64.41922 | 1.47E-130 | 189 | 4149.835 | 0.95644 | 21.9568 | 0.95644 | *** | 9.54E-130 | *** |
| R PuA | CRP | 4.07E-13 | 2.72E-13 | 1.497907 | 0.135825641 | 189 | 2.243726 | 0.011732 | 0.011872 | 0.011732 | ns | 0.220716666 | ns |
| R CM | (Intercept) | 0.000181 | 3.08E-06 | 58.75626 | 2.30E-123 | 189 | 3452.298 | 0.948095 | 18.26613 | 0.948095 | *** | 9.68E-123 | *** |
| R CM | CRP | 3.66E-13 | 3.32E-13 | 1.104687 | 0.270699662 | 189 | 1.220333 | 0.006415 | 0.006457 | 0.006415 | ns | 0.370431116 | ns |
| L LGN | (Intercept) | 0.00019 | 3.87E-06 | 49.03041 | 2.00E-109 | 189 | 2403.981 | 0.927111 | 12.71948 | 0.927111 | *** | 2.89E-109 | *** |
| L LGN | CRP | 6.81E-13 | 4.17E-13 | 1.632004 | 0.104343628 | 189 | 2.663438 | 0.013896 | 0.014092 | 0.013896 | ns | 0.180862289 | ns |
| L MDl | (Intercept) | 0.000183 | 3.29E-06 | 55.82004 | 2.19E-119 | 189 | 3115.877 | 0.942812 | 16.48612 | 0.942812 | *** | 6.34E-119 | *** |
| L MDl | CRP | 7.50E-13 | 3.54E-13 | 2.116104 | 0.035645112 | 189 | 4.477895 | 0.023144 | 0.023693 | 0.023144 | * | 0.092677292 | ns |
| L PuA | (Intercept) | 0.000164 | 3.25E-06 | 50.56564 | 8.88E-112 | 189 | 2556.884 | 0.93117 | 13.52849 | 0.93117 | *** | 1.36E-111 | *** |
| L PuA | CRP | 1.37E-13 | 3.50E-13 | 0.39014 | 0.696872184 | 189 | 0.152209 | 0.000805 | 0.000805 | 0.000805 | ns | 0.754944866 | ns |
| R VA | (Intercept) | 0.000272 | 4.14E-06 | 65.67375 | 4.47E-132 | 189 | 4313.042 | 0.958019 | 22.82033 | 0.958019 | *** | 5.82E-131 | *** |
| R VA | CRP | 8.90E-13 | 4.46E-13 | 1.993495 | 0.047645481 | 189 | 3.974023 | 0.020594 | 0.021027 | 0.020594 | * | 0.11261659 | ns |
| R LGN | (Intercept) | 0.000184 | 3.83E-06 | 47.98815 | 8.59E-108 | 189 | 2302.863 | 0.924153 | 12.18446 | 0.924153 | *** | 1.12E-107 | *** |
| R LGN | CRP | 7.34E-14 | 4.13E-13 | 0.177692 | 0.859155291 | 189 | 0.031574 | 0.000167 | 0.000167 | 0.000167 | ns | 0.893521503 | ns |
| L VA | (Intercept) | 0.000273 | 5.05E-06 | 54.03787 | 7.05E-117 | 189 | 2920.091 | 0.939211 | 15.45022 | 0.939211 | *** | 1.53E-116 | *** |
| L VA | CRP | 9.05E-13 | 5.45E-13 | 1.661205 | 0.098330248 | 189 | 2.759601 | 0.014391 | 0.014601 | 0.014391 | ns | 0.180862289 | ns |
| R VPL | (Intercept) | 0.000606 | 8.27E-06 | 73.22071 | 1.14E-140 | 189 | 5361.272 | 0.965948 | 28.36652 | 0.965948 | *** | 2.98E-139 | *** |
| R VPL | CRP | 6.64E-14 | 8.92E-13 | 0.074458 | 0.940724599 | 189 | 0.005544 | 2.93E-05 | 2.93E-05 | 2.93E-05 | ns | 0.940724599 | ns |
| R MDm | (Intercept) | 0.000554 | 8.78E-06 | 63.02664 | 7.60E-129 | 189 | 3972.357 | 0.954582 | 21.01776 | 0.954582 | *** | 3.95E-128 | *** |
| R MDm | CRP | 1.65E-12 | 9.47E-13 | 1.742944 | 0.082969778 | 189 | 3.037853 | 0.015819 | 0.016073 | 0.015819 | ns | 0.165939557 | ns |
| L MDm | (Intercept) | 0.000554 | 9.49E-06 | 58.3969 | 6.90E-123 | 189 | 3410.198 | 0.947488 | 18.04337 | 0.947488 | *** | 2.24E-122 | *** |
| L MDm | CRP | 1.91E-12 | 1.02E-12 | 1.865143 | 0.063711347 | 189 | 3.478758 | 0.018073 | 0.018406 | 0.018073 | ns | 0.138041253 | ns |
| R PuM | (Intercept) | 0.000856 | 1.32E-05 | 64.91998 | 3.62E-131 | 189 | 4214.603 | 0.957081 | 22.29949 | 0.957081 | *** | 3.13E-130 | *** |
| R PuM | CRP | 1.52E-12 | 1.42E-12 | 1.069463 | 0.286225112 | 189 | 1.143751 | 0.006015 | 0.006052 | 0.006015 | ns | 0.372092646 | ns |
| L PuM | (Intercept) | 0.000855 | 1.57E-05 | 54.32785 | 2.73E-117 | 189 | 2951.515 | 0.939819 | 15.61648 | 0.939819 | *** | 6.44E-117 | *** |
| L PuM | CRP | 9.04E-13 | 1.70E-12 | 0.532983 | 0.594671239 | 189 | 0.284071 | 0.001501 | 0.001503 | 0.001501 | ns | 0.681241415 | ns |
| R Pt | (Intercept) | 5.26E-06 | 1.26E-07 | 41.68433 | 7.62E-98 | 191 | 1737.583 | 0.900964 | 9.097294 | 0.900964 | *** | 1.04E-97 | *** |
| R Pt | NfL | -2.46E-09 | 4.81E-09 | -0.51212 | 0.609155475 | 191 | 0.262272 | 0.001371 | 0.001373 | -0.00137 | ns | 0.633521694 | ns |
| L VAmc | (Intercept) | 2.36E-05 | 5.62E-07 | 41.99186 | 2.15E-98 | 191 | 1763.316 | 0.902268 | 9.232023 | 0.902268 | *** | 3.10E-98 | *** |
| L VAmc | NfL | -2.75E-08 | 2.14E-08 | -1.28444 | 0.200544107 | 191 | 1.649783 | 0.008564 | 0.008638 | -0.00856 | ns | 0.281977366 | ns |
| L MV-re | (Intercept) | 1.15E-05 | 5.67E-07 | 20.26926 | 1.74E-49 | 191 | 410.8427 | 0.682641 | 2.151009 | 0.682641 | *** | 1.74E-49 | *** |
| L MV-re | NfL | -3.62E-08 | 2.16E-08 | -1.67745 | 0.095090561 | 191 | 2.813832 | 0.014518 | 0.014732 | -0.01452 | ns | 0.206029549 | ns |
| R VAmc | (Intercept) | 2.48E-05 | 5.50E-07 | 45.16243 | 6.98E-104 | 191 | 2039.645 | 0.914375 | 10.67877 | 0.914375 | *** | 1.51E-103 | *** |
| R VAmc | NfL | -2.73E-08 | 2.09E-08 | -1.30201 | 0.194479729 | 191 | 1.695239 | 0.008798 | 0.008876 | -0.0088 | ns | 0.281977366 | ns |
| R MV-re | (Intercept) | 1.24E-05 | 5.97E-07 | 20.69277 | 1.15E-50 | 191 | 428.1906 | 0.691533 | 2.241835 | 0.691533 | *** | 1.19E-50 | *** |
| R MV-re | NfL | -3.10E-08 | 2.27E-08 | -1.36147 | 0.174968518 | 191 | 1.853605 | 0.009611 | 0.009705 | -0.00961 | ns | 0.281977366 | ns |
| R LD | (Intercept) | 2.15E-05 | 8.07E-07 | 26.66196 | 2.72E-66 | 191 | 710.8599 | 0.788215 | 3.72178 | 0.788215 | *** | 2.95E-66 | *** |
| R LD | NfL | -9.60E-08 | 3.07E-08 | -3.12385 | 0.002063056 | 191 | 9.758457 | 0.048608 | 0.051091 | -0.04861 | ** | 0.008939911 | ** |
| R Pf | (Intercept) | 4.45E-05 | 1.19E-06 | 37.47158 | 5.76E-90 | 191 | 1404.119 | 0.88026 | 7.35141 | 0.88026 | *** | 7.13E-90 | *** |
| R Pf | NfL | 3.02E-08 | 4.52E-08 | 0.667896 | 0.50500625 | 191 | 0.446085 | 0.00233 | 0.002336 | 0.00233 | ns | 0.570876631 | ns |
| L AV | (Intercept) | 9.22E-05 | 2.14E-06 | 43.11527 | 2.24E-100 | 191 | 1858.927 | 0.906826 | 9.732602 | 0.906826 | *** | 4.15E-100 | *** |
| L AV | NfL | -1.03E-07 | 8.13E-08 | -1.26879 | 0.206060383 | 191 | 1.609826 | 0.008358 | 0.008428 | -0.00836 | ns | 0.281977366 | ns |
| R CeM | (Intercept) | 5.57E-05 | 1.89E-06 | 29.49223 | 4.95E-73 | 191 | 869.7914 | 0.819946 | 4.553882 | 0.819946 | *** | 5.85E-73 | *** |
| R CeM | NfL | -6.23E-08 | 7.19E-08 | -0.86634 | 0.387389931 | 191 | 0.750547 | 0.003914 | 0.00393 | -0.00391 | ns | 0.479625628 | ns |
| L CeM | (Intercept) | 5.36E-05 | 2.01E-06 | 26.71955 | 1.97E-66 | 191 | 713.9342 | 0.788935 | 3.737875 | 0.788935 | *** | 2.22E-66 | *** |
| L CeM | NfL | -5.74E-08 | 7.63E-08 | -0.75201 | 0.452971 | 191 | 0.565518 | 0.002952 | 0.002961 | -0.00295 | ns | 0.535329364 | ns |
| L MGN | (Intercept) | 9.12E-05 | 2.16E-06 | 42.24851 | 7.50E-99 | 191 | 1784.937 | 0.903337 | 9.345218 | 0.903337 | *** | 1.15E-98 | *** |
| L MGN | NfL | -1.14E-07 | 8.22E-08 | -1.38254 | 0.168420002 | 191 | 1.91142 | 0.009908 | 0.010007 | -0.00991 | ns | 0.281977366 | ns |
| R MGN | (Intercept) | 9.93E-05 | 2.57E-06 | 38.67396 | 2.78E-92 | 191 | 1495.675 | 0.886759 | 7.83076 | 0.886759 | *** | 3.61E-92 | *** |
| R MGN | NfL | -1.05E-07 | 9.77E-08 | -1.07486 | 0.283791718 | 191 | 1.155332 | 0.006012 | 0.006049 | -0.00601 | ns | 0.368929234 | ns |
| R MDl | (Intercept) | 0.000204 | 3.99E-06 | 51.19085 | 1.74E-113 | 191 | 2620.503 | 0.932065 | 13.71991 | 0.932065 | *** | 7.55E-113 | *** |
| R MDl | NfL | -5.58E-07 | 1.52E-07 | -3.67842 | 0.000305067 | 191 | 13.53078 | 0.066155 | 0.070842 | -0.06616 | *** | 0.002643916 | ** |
| R PuA | (Intercept) | 0.000173 | 3.17E-06 | 54.5825 | 1.81E-118 | 191 | 2979.25 | 0.939752 | 15.59817 | 0.939752 | *** | 1.57E-117 | *** |
| R PuA | NfL | -3.64E-07 | 1.21E-07 | -3.01325 | 0.002935135 | 191 | 9.079666 | 0.04538 | 0.047538 | -0.04538 | ** | 0.01090193 | * |
| R CM | (Intercept) | 0.000182 | 3.95E-06 | 46.1634 | 1.50E-105 | 191 | 2131.059 | 0.917745 | 11.15738 | 0.917745 | *** | 3.91E-105 | *** |
| R CM | NfL | 3.95E-08 | 1.50E-07 | 0.262639 | 0.793112239 | 191 | 0.068979 | 0.000361 | 0.000361 | 0.000361 | ns | 0.793112239 | ns |
| L LGN | (Intercept) | 0.000213 | 4.65E-06 | 45.88573 | 4.33E-105 | 191 | 2105.5 | 0.91683 | 11.02356 | 0.91683 | *** | 1.02E-104 | *** |
| L LGN | NfL | -8.90E-07 | 1.77E-07 | -5.02935 | 1.13E-06 | 191 | 25.29433 | 0.116944 | 0.132431 | -0.11694 | *** | 2.94E-05 | *** |
| L MDl | (Intercept) | 0.000196 | 4.21E-06 | 46.56402 | 3.30E-106 | 191 | 2168.208 | 0.919041 | 11.35188 | 0.919041 | *** | 1.07E-105 | *** |
| L MDl | NfL | -3.72E-07 | 1.60E-07 | -2.31824 | 0.021495249 | 191 | 5.374229 | 0.027367 | 0.028137 | -0.02737 | * | 0.055887649 | ns |
| L PuA | (Intercept) | 0.000173 | 4.09E-06 | 42.3646 | 4.67E-99 | 191 | 1794.76 | 0.903815 | 9.396647 | 0.903815 | *** | 7.59E-99 | *** |
| L PuA | NfL | -3.77E-07 | 1.56E-07 | -2.42153 | 0.016389997 | 191 | 5.863816 | 0.029786 | 0.030701 | -0.02979 | * | 0.047348879 | * |
| R VA | (Intercept) | 0.000284 | 5.35E-06 | 53.09919 | 2.53E-116 | 191 | 2819.524 | 0.936556 | 14.76191 | 0.936556 | *** | 1.32E-115 | *** |
| R VA | NfL | -3.03E-07 | 2.04E-07 | -1.4874 | 0.138559109 | 191 | 2.212349 | 0.01145 | 0.011583 | -0.01145 | ns | 0.25732406 | ns |
| R LGN | (Intercept) | 0.000201 | 4.73E-06 | 42.5089 | 2.59E-99 | 191 | 1807.006 | 0.904405 | 9.460766 | 0.904405 | *** | 4.50E-99 | *** |
| R LGN | NfL | -7.33E-07 | 1.80E-07 | -4.07172 | 6.83E-05 | 191 | 16.57887 | 0.079868 | 0.0868 | -0.07987 | *** | 0.000887996 | *** |
| L VA | (Intercept) | 0.000288 | 6.54E-06 | 43.99027 | 6.83E-102 | 191 | 1935.144 | 0.910166 | 10.13164 | 0.910166 | *** | 1.37E-101 | *** |
| L VA | NfL | -3.80E-07 | 2.49E-07 | -1.52586 | 0.128699977 | 191 | 2.328236 | 0.012043 | 0.01219 | -0.01204 | ns | 0.25732406 | ns |
| R VPL | (Intercept) | 0.000601 | 1.06E-05 | 56.57146 | 2.90E-121 | 191 | 3200.33 | 0.94368 | 16.75565 | 0.94368 | *** | 7.53E-120 | *** |
| R VPL | NfL | 2.29E-07 | 4.05E-07 | 0.565706 | 0.572257413 | 191 | 0.320024 | 0.001673 | 0.001676 | 0.001673 | ns | 0.61994553 | ns |
| R MDm | (Intercept) | 0.000589 | 1.10E-05 | 53.4383 | 8.10E-117 | 191 | 2855.652 | 0.937308 | 14.95106 | 0.937308 | *** | 5.26E-116 | *** |
| R MDm | NfL | -1.16E-06 | 4.19E-07 | -2.77228 | 0.00611775 | 191 | 7.685522 | 0.038682 | 0.040238 | -0.03868 | ** | 0.019882689 | * |
| L MDm | (Intercept) | 0.000586 | 1.21E-05 | 48.42197 | 3.35E-109 | 191 | 2344.687 | 0.924675 | 12.27585 | 0.924675 | *** | 1.24E-108 | *** |
| L MDm | NfL | -9.76E-07 | 4.61E-07 | -2.11698 | 0.035555556 | 191 | 4.481624 | 0.022926 | 0.023464 | -0.02293 | * | 0.084040405 | ns |
| R PuM | (Intercept) | 0.000914 | 1.66E-05 | 54.93631 | 5.68E-119 | 191 | 3017.999 | 0.94048 | 15.80104 | 0.94048 | *** | 7.39E-118 | *** |
| R PuM | NfL | -2.18E-06 | 6.33E-07 | -3.44901 | 0.000692262 | 191 | 11.89567 | 0.058629 | 0.062281 | -0.05863 | *** | 0.0044997 | ** |
| L PuM | (Intercept) | 0.000909 | 1.96E-05 | 46.40179 | 6.09E-106 | 191 | 2153.126 | 0.91852 | 11.27291 | 0.91852 | *** | 1.76E-105 | *** |
| L PuM | NfL | -2.36E-06 | 7.45E-07 | -3.16888 | 0.001782277 | 191 | 10.04181 | 0.049949 | 0.052575 | -0.04995 | ** | 0.008939911 | ** |
| R Pt | (Intercept) | 5.46E-06 | 1.45E-07 | 37.61495 | 5.67E-90 | 190 | 1414.885 | 0.881611 | 7.446762 | 0.881611 | *** | 8.67E-90 | *** |
| R Pt | pTau181 | -7.79E-08 | 3.86E-08 | -2.01678 | 0.045125685 | 190 | 4.067419 | 0.020959 | 0.021407 | -0.02096 | * | 0.053330355 | ns |
| L VAmc | (Intercept) | 2.44E-05 | 6.50E-07 | 37.49518 | 9.67E-90 | 190 | 1405.888 | 0.880944 | 7.399413 | 0.880944 | *** | 1.40E-89 | *** |
| L VAmc | pTau181 | -4.35E-07 | 1.73E-07 | -2.51251 | 0.012819742 | 190 | 6.312695 | 0.032156 | 0.033225 | -0.03216 | * | 0.017542805 | * |
| L MV-re | (Intercept) | 1.21E-05 | 6.57E-07 | 18.43416 | 3.52E-44 | 190 | 339.8182 | 0.641386 | 1.788517 | 0.641386 | *** | 3.52E-44 | *** |
| L MV-re | pTau181 | -4.42E-07 | 1.75E-07 | -2.52374 | 0.012430279 | 190 | 6.369289 | 0.032435 | 0.033523 | -0.03244 | * | 0.017542805 | * |
| R VAmc | (Intercept) | 2.58E-05 | 6.30E-07 | 40.97321 | 2.94E-96 | 190 | 1678.804 | 0.898331 | 8.835811 | 0.898331 | *** | 6.94E-96 | *** |
| R VAmc | pTau181 | -4.93E-07 | 1.68E-07 | -2.93903 | 0.003700649 | 190 | 8.637868 | 0.043486 | 0.045462 | -0.04349 | ** | 0.006872634 | ** |
| R MV-re | (Intercept) | 1.34E-05 | 6.89E-07 | 19.47079 | 3.86E-47 | 190 | 379.1118 | 0.666146 | 1.995325 | 0.666146 | *** | 4.02E-47 | *** |
| R MV-re | pTau181 | -5.41E-07 | 1.83E-07 | -2.95214 | 0.003553653 | 190 | 8.71516 | 0.043858 | 0.045869 | -0.04386 | ** | 0.006872634 | ** |
| R LD | (Intercept) | 2.26E-05 | 9.26E-07 | 24.46613 | 1.26E-60 | 190 | 598.5917 | 0.759064 | 3.150483 | 0.759064 | *** | 1.43E-60 | *** |
| R LD | pTau181 | -1.01E-06 | 2.46E-07 | -4.11828 | 5.69E-05 | 190 | 16.96026 | 0.081949 | 0.089265 | -0.08195 | *** | 0.000246567 | *** |
| R Pf | (Intercept) | 4.63E-05 | 1.37E-06 | 33.69316 | 4.56E-82 | 190 | 1135.229 | 0.856629 | 5.974888 | 0.856629 | *** | 5.65E-82 | *** |
| R Pf | pTau181 | -4.01E-07 | 3.66E-07 | -1.0951 | 0.274857589 | 190 | 1.199251 | 0.006272 | 0.006312 | -0.00627 | ns | 0.274857589 | ns |
| L AV | (Intercept) | 9.70E-05 | 2.44E-06 | 39.79688 | 4.19E-94 | 190 | 1583.792 | 0.892885 | 8.335747 | 0.892885 | *** | 8.38E-94 | *** |
| L AV | pTau181 | -2.25E-06 | 6.49E-07 | -3.46413 | 0.000657376 | 190 | 12.0002 | 0.059407 | 0.063159 | -0.05941 | *** | 0.002441681 | ** |
| R CeM | (Intercept) | 5.93E-05 | 2.18E-06 | 27.16289 | 2.42E-67 | 190 | 737.8228 | 0.79522 | 3.883278 | 0.79522 | *** | 2.86E-67 | *** |
| R CeM | pTau181 | -1.54E-06 | 5.81E-07 | -2.65993 | 0.008483385 | 190 | 7.075231 | 0.035901 | 0.037238 | -0.0359 | ** | 0.014704533 | * |
| L CeM | (Intercept) | 5.68E-05 | 2.32E-06 | 24.42281 | 1.63E-60 | 190 | 596.4735 | 0.758415 | 3.139334 | 0.758415 | *** | 1.77E-60 | *** |
| L CeM | pTau181 | -1.38E-06 | 6.19E-07 | -2.22448 | 0.027293225 | 190 | 4.948316 | 0.025383 | 0.026044 | -0.02538 | * | 0.033791612 | * |
| L MGN | (Intercept) | 9.43E-05 | 2.49E-06 | 37.92643 | 1.42E-90 | 190 | 1438.414 | 0.883322 | 7.570599 | 0.883322 | *** | 2.31E-90 | *** |
| L MGN | pTau181 | -1.74E-06 | 6.62E-07 | -2.63465 | 0.009117286 | 190 | 6.94136 | 0.035246 | 0.036533 | -0.03525 | ** | 0.01481559 | * |
| R MGN | (Intercept) | 0.000101 | 2.95E-06 | 34.24199 | 3.26E-83 | 190 | 1172.514 | 0.860552 | 6.171127 | 0.860552 | *** | 4.24E-83 | *** |
| R MGN | pTau181 | -1.41E-06 | 7.86E-07 | -1.79425 | 0.074363931 | 190 | 3.219316 | 0.016661 | 0.016944 | -0.01666 | ns | 0.084063574 | ns |
| R MDl | (Intercept) | 0.000209 | 4.61E-06 | 45.25711 | 1.05E-103 | 190 | 2048.206 | 0.915111 | 10.78003 | 0.915111 | *** | 4.56E-103 | *** |
| R MDl | pTau181 | -5.16E-06 | 1.23E-06 | -4.20366 | 4.04E-05 | 190 | 17.67078 | 0.08509 | 0.093004 | -0.08509 | *** | 0.000210067 | *** |
| R PuA | (Intercept) | 0.000178 | 3.61E-06 | 49.31249 | 3.12E-110 | 190 | 2431.721 | 0.927529 | 12.79853 | 0.927529 | *** | 2.70E-109 | *** |
| R PuA | pTau181 | -4.08E-06 | 9.61E-07 | -4.24264 | 3.45E-05 | 190 | 18.00003 | 0.086539 | 0.094737 | -0.08654 | *** | 0.000210067 | *** |
| R CM | (Intercept) | 0.000188 | 4.51E-06 | 41.71445 | 1.37E-97 | 190 | 1740.095 | 0.901559 | 9.158395 | 0.901559 | *** | 3.95E-97 | *** |
| R CM | pTau181 | -1.68E-06 | 1.20E-06 | -1.39495 | 0.164660862 | 190 | 1.945873 | 0.010138 | 0.010241 | -0.01014 | ns | 0.171247297 | ns |
| L LGN | (Intercept) | 0.00022 | 5.31E-06 | 41.42807 | 4.45E-97 | 190 | 1716.285 | 0.90033 | 9.033077 | 0.90033 | *** | 1.16E-96 | *** |
| L LGN | pTau181 | -8.09E-06 | 1.41E-06 | -5.71539 | 4.18E-08 | 190 | 32.66566 | 0.146703 | 0.171925 | -0.1467 | *** | 5.43E-07 | *** |
| L MDl | (Intercept) | 0.000202 | 4.83E-06 | 41.75343 | 1.16E-97 | 190 | 1743.349 | 0.901725 | 9.175523 | 0.901725 | *** | 3.79E-97 | *** |
| L MDl | pTau181 | -4.30E-06 | 1.29E-06 | -3.34401 | 0.000994776 | 190 | 11.18238 | 0.055583 | 0.058855 | -0.05558 | *** | 0.002873798 | ** |
| L PuA | (Intercept) | 0.000174 | 4.74E-06 | 36.74074 | 2.89E-88 | 190 | 1349.882 | 0.876614 | 7.104641 | 0.876614 | *** | 3.95E-88 | *** |
| L PuA | pTau181 | -2.94E-06 | 1.26E-06 | -2.32896 | 0.020912527 | 190 | 5.424069 | 0.027755 | 0.028548 | -0.02776 | * | 0.027186285 | * |
| R VA | (Intercept) | 0.000294 | 6.13E-06 | 47.99427 | 3.67E-108 | 190 | 2303.45 | 0.9238 | 12.12342 | 0.9238 | *** | 2.38E-107 | *** |
| R VA | pTau181 | -5.01E-06 | 1.63E-06 | -3.07121 | 0.002444597 | 190 | 9.432358 | 0.047296 | 0.049644 | -0.0473 | ** | 0.006355952 | ** |
| R LGN | (Intercept) | 0.000211 | 5.31E-06 | 39.77016 | 4.70E-94 | 190 | 1581.665 | 0.892756 | 8.324554 | 0.892756 | *** | 8.72E-94 | *** |
| R LGN | pTau181 | -8.19E-06 | 1.41E-06 | -5.79506 | 2.80E-08 | 190 | 33.58274 | 0.150203 | 0.176751 | -0.1502 | *** | 5.43E-07 | *** |
| L VA | (Intercept) | 0.000296 | 7.57E-06 | 39.0681 | 9.59E-93 | 190 | 1526.316 | 0.889298 | 8.033243 | 0.889298 | *** | 1.66E-92 | *** |
| L VA | pTau181 | -5.09E-06 | 2.02E-06 | -2.52531 | 0.012377014 | 190 | 6.377171 | 0.032474 | 0.033564 | -0.03247 | * | 0.017542805 | * |
| R VPL | (Intercept) | 0.000621 | 1.21E-05 | 51.53077 | 1.30E-113 | 190 | 2655.421 | 0.933226 | 13.9759 | 0.933226 | *** | 3.38E-112 | *** |
| R VPL | pTau181 | -4.93E-06 | 3.21E-06 | -1.53764 | 0.125799735 | 190 | 2.364344 | 0.012291 | 0.012444 | -0.01229 | ns | 0.136283046 | ns |
| R MDm | (Intercept) | 0.0006 | 1.28E-05 | 47.02453 | 1.31E-106 | 190 | 2211.307 | 0.920876 | 11.63846 | 0.920876 | *** | 6.84E-106 | *** |
| R MDm | pTau181 | -1.16E-05 | 3.40E-06 | -3.39832 | 0.000826004 | 190 | 11.54856 | 0.057299 | 0.060782 | -0.0573 | *** | 0.002684514 | ** |
| L MDm | (Intercept) | 0.000601 | 1.39E-05 | 43.13478 | 4.32E-100 | 190 | 1860.609 | 0.907345 | 9.79268 | 0.907345 | *** | 1.60E-99 | *** |
| L MDm | pTau181 | -1.12E-05 | 3.71E-06 | -3.02683 | 0.002814129 | 190 | 9.161679 | 0.046001 | 0.048219 | -0.046 | ** | 0.006440125 | ** |
| R PuM | (Intercept) | 0.000939 | 1.89E-05 | 49.73935 | 6.82E-111 | 190 | 2474.003 | 0.928679 | 13.02107 | 0.928679 | *** | 8.86E-110 | *** |
| R PuM | pTau181 | -2.27E-05 | 5.03E-06 | -4.52161 | 1.08E-05 | 190 | 20.44498 | 0.097151 | 0.107605 | -0.09715 | *** | 9.35E-05 | *** |
| L PuM | (Intercept) | 0.000917 | 2.27E-05 | 40.35637 | 3.90E-95 | 190 | 1628.637 | 0.895526 | 8.571772 | 0.895526 | *** | 8.46E-95 | *** |
| L PuM | pTau181 | -1.82E-05 | 6.05E-06 | -3.00944 | 0.002972365 | 190 | 9.056748 | 0.045498 | 0.047667 | -0.0455 | ** | 0.006440125 | ** |
| R Pt | (Intercept) | 5.42E-06 | 1.32E-07 | 40.908 | 2.65E-87 | 162 | 1673.465 | 0.911739 | 10.33003 | 0.911739 | *** | 4.31E-87 | *** |
| R Pt | pTau217 | -2.89E-07 | 1.37E-07 | -2.10462 | 0.036867322 | 162 | 4.42944 | 0.026615 | 0.027342 | -0.02661 | * | 0.053252798 | ns |
| L VAmc | (Intercept) | 2.40E-05 | 6.10E-07 | 39.27896 | 1.05E-84 | 162 | 1542.837 | 0.904976 | 9.523683 | 0.904976 | *** | 1.52E-84 | *** |
| L VAmc | pTau217 | -1.37E-06 | 6.32E-07 | -2.17372 | 0.031177674 | 162 | 4.725068 | 0.02834 | 0.029167 | -0.02834 | * | 0.05066372 | ns |
| L MV-re | (Intercept) | 1.18E-05 | 5.96E-07 | 19.79488 | 4.24E-45 | 162 | 391.8374 | 0.707495 | 2.418749 | 0.707495 | *** | 4.24E-45 | *** |
| L MV-re | pTau217 | -1.47E-06 | 6.17E-07 | -2.38895 | 0.018046776 | 162 | 5.707063 | 0.03403 | 0.035229 | -0.03403 | * | 0.03351544 | * |
| R VAmc | (Intercept) | 2.56E-05 | 5.72E-07 | 44.77029 | 3.89E-93 | 162 | 2004.379 | 0.925221 | 12.37271 | 0.925221 | *** | 1.26E-92 | *** |
| R VAmc | pTau217 | -1.89E-06 | 5.92E-07 | -3.19545 | 0.001678422 | 162 | 10.21092 | 0.059293 | 0.06303 | -0.05929 | ** | 0.004808519 | ** |
| R MV-re | (Intercept) | 1.32E-05 | 6.07E-07 | 21.79588 | 4.91E-50 | 162 | 475.0604 | 0.745707 | 2.932472 | 0.745707 | *** | 5.11E-50 | *** |
| R MV-re | pTau217 | -2.01E-06 | 6.28E-07 | -3.19218 | 0.001696422 | 162 | 10.19 | 0.059179 | 0.062901 | -0.05918 | ** | 0.004808519 | ** |
| R LD | (Intercept) | 2.20E-05 | 8.31E-07 | 26.50395 | 8.62E-61 | 162 | 702.4596 | 0.8126 | 4.33617 | 0.8126 | *** | 9.74E-61 | *** |
| R LD | pTau217 | -3.36E-06 | 8.61E-07 | -3.89873 | 0.000141338 | 162 | 15.20007 | 0.085779 | 0.093828 | -0.08578 | *** | 0.000613915 | *** |
| R Pf | (Intercept) | 4.62E-05 | 1.29E-06 | 35.87587 | 5.55E-79 | 162 | 1287.078 | 0.888205 | 7.944926 | 0.888205 | *** | 6.87E-79 | *** |
| R Pf | pTau217 | -1.56E-06 | 1.33E-06 | -1.16675 | 0.245026331 | 162 | 1.361304 | 0.008333 | 0.008403 | -0.00833 | ns | 0.245026331 | ns |
| L AV | (Intercept) | 9.57E-05 | 2.27E-06 | 42.22555 | 2.42E-89 | 162 | 1782.997 | 0.916709 | 11.00616 | 0.916709 | *** | 5.71E-89 | *** |
| L AV | pTau217 | -7.37E-06 | 2.35E-06 | -3.14024 | 0.002006855 | 162 | 9.861086 | 0.057378 | 0.060871 | -0.05738 | ** | 0.004808519 | ** |
| R CeM | (Intercept) | 5.90E-05 | 1.92E-06 | 30.76033 | 1.54E-69 | 162 | 946.198 | 0.853817 | 5.840729 | 0.853817 | *** | 1.82E-69 | *** |
| R CeM | pTau217 | -6.23E-06 | 1.99E-06 | -3.136 | 0.002034373 | 162 | 9.834491 | 0.057232 | 0.060707 | -0.05723 | ** | 0.004808519 | ** |
| L CeM | (Intercept) | 5.54E-05 | 2.12E-06 | 26.09587 | 6.62E-60 | 162 | 680.9943 | 0.807828 | 4.203668 | 0.807828 | *** | 7.18E-60 | *** |
| L CeM | pTau217 | -4.02E-06 | 2.20E-06 | -1.82611 | 0.069674499 | 162 | 3.334684 | 0.020169 | 0.020584 | -0.02017 | ns | 0.082342589 | ns |
| L MGN | (Intercept) | 9.16E-05 | 2.24E-06 | 40.79702 | 3.96E-87 | 162 | 1664.397 | 0.911301 | 10.27405 | 0.911301 | *** | 6.06E-87 | *** |
| L MGN | pTau217 | -4.63E-06 | 2.32E-06 | -1.99127 | 0.048132805 | 162 | 3.965139 | 0.023891 | 0.024476 | -0.02389 | * | 0.059592997 | ns |
| R MGN | (Intercept) | 0.0001 | 2.74E-06 | 36.58838 | 3.25E-80 | 162 | 1338.71 | 0.892051 | 8.26364 | 0.892051 | *** | 4.22E-80 | *** |
| R MGN | pTau217 | -4.90E-06 | 2.83E-06 | -1.73087 | 0.085379393 | 162 | 2.995903 | 0.018157 | 0.018493 | -0.01816 | ns | 0.096515835 | ns |
| R MDl | (Intercept) | 0.000203 | 3.98E-06 | 51.07444 | 8.54E-102 | 162 | 2608.598 | 0.941529 | 16.10246 | 0.941529 | *** | 3.70E-101 | *** |
| R MDl | pTau217 | -1.69E-05 | 4.12E-06 | -4.09757 | 6.58E-05 | 162 | 16.79005 | 0.093909 | 0.103642 | -0.09391 | *** | 0.000570567 | *** |
| R PuA | (Intercept) | 0.000177 | 3.22E-06 | 54.81344 | 1.70E-106 | 162 | 3004.514 | 0.94884 | 18.54638 | 0.94884 | *** | 2.21E-105 | *** |
| R PuA | pTau217 | -1.51E-05 | 3.34E-06 | -4.52382 | 1.17E-05 | 162 | 20.46492 | 0.112158 | 0.126327 | -0.11216 | *** | 0.000303981 | *** |
| R CM | (Intercept) | 0.000187 | 4.21E-06 | 44.52536 | 8.84E-93 | 162 | 1982.508 | 0.924458 | 12.2377 | 0.924458 | *** | 2.55E-92 | *** |
| R CM | pTau217 | -6.49E-06 | 4.36E-06 | -1.48956 | 0.138284045 | 162 | 2.218791 | 0.013511 | 0.013696 | -0.01351 | ns | 0.143815406 | ns |
| L LGN | (Intercept) | 0.000211 | 5.09E-06 | 41.36556 | 5.12E-88 | 162 | 1711.11 | 0.913513 | 10.5624 | 0.913513 | *** | 1.11E-87 | *** |
| L LGN | pTau217 | -2.06E-05 | 5.27E-06 | -3.8981 | 0.000141673 | 162 | 15.19518 | 0.085754 | 0.093797 | -0.08575 | *** | 0.000613915 | *** |
| L MDl | (Intercept) | 0.000195 | 4.42E-06 | 44.21186 | 2.55E-92 | 162 | 1954.689 | 0.923465 | 12.06598 | 0.923465 | *** | 6.62E-92 | *** |
| L MDl | pTau217 | -1.14E-05 | 4.57E-06 | -2.49823 | 0.013479028 | 162 | 6.24116 | 0.037097 | 0.038526 | -0.0371 | * | 0.026958057 | * |
| L PuA | (Intercept) | 0.000173 | 4.44E-06 | 38.90912 | 4.21E-84 | 162 | 1513.92 | 0.903337 | 9.345183 | 0.903337 | *** | 5.76E-84 | *** |
| L PuA | pTau217 | -9.84E-06 | 4.59E-06 | -2.14211 | 0.033678578 | 162 | 4.588644 | 0.027545 | 0.028325 | -0.02754 | * | 0.051508414 | ns |
| R VA | (Intercept) | 0.000293 | 5.64E-06 | 51.90852 | 7.20E-103 | 162 | 2694.495 | 0.943287 | 16.63268 | 0.943287 | *** | 4.68E-102 | *** |
| R VA | pTau217 | -2.03E-05 | 5.84E-06 | -3.47456 | 0.000656609 | 162 | 12.07256 | 0.069354 | 0.074522 | -0.06935 | *** | 0.002438833 | ** |
| R LGN | (Intercept) | 0.000202 | 4.89E-06 | 41.30765 | 6.30E-88 | 162 | 1706.322 | 0.913291 | 10.53285 | 0.913291 | *** | 1.26E-87 | *** |
| R LGN | pTau217 | -2.03E-05 | 5.07E-06 | -4.00227 | 9.53E-05 | 162 | 16.01816 | 0.089981 | 0.098878 | -0.08998 | *** | 0.000613915 | *** |
| L VA | (Intercept) | 0.000292 | 7.10E-06 | 41.07508 | 1.45E-87 | 162 | 1687.162 | 0.912393 | 10.41458 | 0.912393 | *** | 2.52E-87 | *** |
| L VA | pTau217 | -1.52E-05 | 7.35E-06 | -2.06623 | 0.040398857 | 162 | 4.269299 | 0.025677 | 0.026354 | -0.02568 | * | 0.055282647 | ns |
| R VPL | (Intercept) | 0.00062 | 1.12E-05 | 55.54199 | 2.23E-107 | 162 | 3084.913 | 0.950106 | 19.04267 | 0.950106 | *** | 5.81E-106 | *** |
| R VPL | pTau217 | -1.89E-05 | 1.16E-05 | -1.63918 | 0.103116537 | 162 | 2.686908 | 0.016315 | 0.016586 | -0.01632 | ns | 0.111709582 | ns |
| R MDm | (Intercept) | 0.000588 | 1.14E-05 | 51.52228 | 2.25E-102 | 162 | 2654.546 | 0.942483 | 16.38608 | 0.942483 | *** | 1.17E-101 | *** |
| R MDm | pTau217 | -3.48E-05 | 1.18E-05 | -2.94494 | 0.003705946 | 162 | 8.672656 | 0.050815 | 0.053535 | -0.05081 | ** | 0.00802955 | ** |
| L MDm | (Intercept) | 0.000586 | 1.30E-05 | 45.27516 | 7.22E-94 | 162 | 2049.84 | 0.926758 | 12.65333 | 0.926758 | *** | 2.68E-93 | *** |
| L MDm | pTau217 | -3.10E-05 | 1.34E-05 | -2.31051 | 0.022121313 | 162 | 5.338434 | 0.031902 | 0.032953 | -0.0319 | * | 0.03834361 | * |
| R PuM | (Intercept) | 0.000927 | 1.74E-05 | 53.40711 | 9.22E-105 | 162 | 2852.319 | 0.946257 | 17.60691 | 0.946257 | *** | 7.99E-104 | *** |
| R PuM | pTau217 | -7.39E-05 | 1.80E-05 | -4.11172 | 6.23E-05 | 162 | 16.90626 | 0.094498 | 0.10436 | -0.0945 | *** | 0.000570567 | *** |
| L PuM | (Intercept) | 0.000897 | 2.18E-05 | 41.24201 | 7.97E-88 | 162 | 1700.903 | 0.913039 | 10.4994 | 0.913039 | *** | 1.48E-87 | *** |
| L PuM | pTau217 | -4.58E-05 | 2.25E-05 | -2.03299 | 0.043686309 | 162 | 4.133037 | 0.024878 | 0.025513 | -0.02488 | * | 0.056792202 | ns |
| R Pt | (Intercept) | 4.86E-06 | 1.30E-07 | 37.48597 | 1.33E-104 | 250 | 1405.198 | 0.848961 | 5.620792 | 0.848961 | *** | 2.66E-104 | *** |
| R Pt | total_free_recall | 2.08E-08 | 7.33E-09 | 2.835167 | 0.004954735 | 250 | 8.038172 | 0.031151 | 0.032153 | 0.031151 | ** | 0.005855596 | ** |
| L VAmc | (Intercept) | 2.14E-05 | 5.75E-07 | 37.23531 | 5.52E-104 | 250 | 1386.468 | 0.847232 | 5.545873 | 0.847232 | *** | 1.03E-103 | *** |
| L VAmc | total_free_recall | 9.75E-08 | 3.25E-08 | 2.997092 | 0.003000156 | 250 | 8.982562 | 0.034684 | 0.03593 | 0.034684 | ** | 0.003900203 | ** |
| L MV-re | (Intercept) | 9.00E-06 | 5.96E-07 | 15.09182 | 5.04E-37 | 250 | 227.763 | 0.476728 | 0.911052 | 0.476728 | *** | 5.04E-37 | *** |
| L MV-re | total_free_recall | 1.03E-07 | 3.38E-08 | 3.057017 | 0.002478171 | 250 | 9.345352 | 0.036034 | 0.037381 | 0.036034 | ** | 0.003391182 | ** |
| R VAmc | (Intercept) | 2.24E-05 | 5.62E-07 | 39.79846 | 3.58E-110 | 250 | 1583.918 | 0.86368 | 6.33567 | 0.86368 | *** | 1.03E-109 | *** |
| R VAmc | total_free_recall | 1.17E-07 | 3.18E-08 | 3.676085 | 0.000289856 | 250 | 13.5136 | 0.051282 | 0.054054 | 0.051282 | *** | 0.000628021 | *** |
| R MV-re | (Intercept) | 9.59E-06 | 6.33E-07 | 15.13645 | 3.54E-37 | 250 | 229.1123 | 0.478202 | 0.916449 | 0.478202 | *** | 3.68E-37 | *** |
| R MV-re | total_free_recall | 1.32E-07 | 3.58E-08 | 3.681559 | 0.000284017 | 250 | 13.55388 | 0.051427 | 0.054216 | 0.051427 | *** | 0.000628021 | *** |
| R LD | (Intercept) | 1.49E-05 | 8.83E-07 | 16.90758 | 2.80E-43 | 250 | 285.8664 | 0.533466 | 1.143465 | 0.533466 | *** | 3.04E-43 | *** |
| R LD | total_free_recall | 2.64E-07 | 5.00E-08 | 5.28216 | 2.78E-07 | 250 | 27.90121 | 0.1004 | 0.111605 | 0.1004 | *** | 3.61E-06 | *** |
| R Pf | (Intercept) | 4.26E-05 | 1.23E-06 | 34.54767 | 3.61E-97 | 250 | 1193.542 | 0.826815 | 4.774167 | 0.826815 | *** | 5.21E-97 | *** |
| R Pf | total_free_recall | 1.61E-07 | 6.97E-08 | 2.303999 | 0.02204331 | 250 | 5.308413 | 0.020792 | 0.021234 | 0.020792 | * | 0.024119553 | * |
| L AV | (Intercept) | 8.30E-05 | 2.25E-06 | 36.86557 | 4.57E-103 | 250 | 1359.07 | 0.844631 | 5.436281 | 0.844631 | *** | 7.91E-103 | *** |
| L AV | total_free_recall | 3.92E-07 | 1.27E-07 | 3.079484 | 0.002305034 | 250 | 9.483222 | 0.036547 | 0.037933 | 0.036547 | ** | 0.003329494 | ** |
| R CeM | (Intercept) | 4.80E-05 | 2.00E-06 | 24.06072 | 5.11E-67 | 250 | 578.9184 | 0.698402 | 2.315674 | 0.698402 | *** | 6.04E-67 | *** |
| R CeM | total_free_recall | 4.00E-07 | 1.13E-07 | 3.543912 | 0.000470364 | 250 | 12.55931 | 0.047834 | 0.050237 | 0.047834 | *** | 0.000873532 | *** |
| L CeM | (Intercept) | 4.66E-05 | 2.09E-06 | 22.319 | 1.93E-61 | 250 | 498.1379 | 0.665837 | 1.992552 | 0.665837 | *** | 2.18E-61 | *** |
| L CeM | total_free_recall | 3.51E-07 | 1.18E-07 | 2.970563 | 0.003262007 | 250 | 8.824242 | 0.034094 | 0.035297 | 0.034094 | ** | 0.004038675 | ** |
| L MGN | (Intercept) | 8.12E-05 | 2.21E-06 | 36.65282 | 1.55E-102 | 250 | 1343.429 | 0.843106 | 5.373718 | 0.843106 | *** | 2.52E-102 | *** |
| L MGN | total_free_recall | 4.55E-07 | 1.25E-07 | 3.628748 | 0.000345285 | 250 | 13.16781 | 0.050036 | 0.052671 | 0.050036 | *** | 0.00069057 | *** |
| R MGN | (Intercept) | 9.15E-05 | 2.68E-06 | 34.09381 | 5.53E-96 | 250 | 1162.388 | 0.822995 | 4.649552 | 0.822995 | *** | 7.57E-96 | *** |
| R MGN | total_free_recall | 3.49E-07 | 1.52E-07 | 2.300145 | 0.022264203 | 250 | 5.290666 | 0.020724 | 0.021163 | 0.020724 | * | 0.024119553 | * |
| R MDl | (Intercept) | 0.000172 | 4.26E-06 | 40.30732 | 2.29E-111 | 250 | 1624.68 | 0.866644 | 6.498719 | 0.866644 | *** | 7.44E-111 | *** |
| R MDl | total_free_recall | 1.19E-06 | 2.41E-07 | 4.940276 | 1.43E-06 | 250 | 24.40632 | 0.088942 | 0.097625 | 0.088942 | *** | 9.29E-06 | *** |
| R PuA | (Intercept) | 0.000151 | 3.38E-06 | 44.57876 | 5.70E-121 | 250 | 1987.266 | 0.888256 | 7.949063 | 0.888256 | *** | 3.70E-120 | *** |
| R PuA | total_free_recall | 9.03E-07 | 1.92E-07 | 4.71325 | 4.05E-06 | 250 | 22.21473 | 0.081607 | 0.088859 | 0.081607 | *** | 2.11E-05 | *** |
| R CM | (Intercept) | 0.000176 | 4.04E-06 | 43.44024 | 1.76E-118 | 250 | 1887.054 | 0.883017 | 7.548218 | 0.883017 | *** | 7.62E-118 | *** |
| R CM | total_free_recall | 4.21E-07 | 2.29E-07 | 1.840485 | 0.066882241 | 250 | 3.387385 | 0.013368 | 0.01355 | 0.013368 | ns | 0.069557531 | ns |
| L LGN | (Intercept) | 0.000167 | 4.90E-06 | 33.99156 | 1.03E-95 | 250 | 1155.426 | 0.822118 | 4.621704 | 0.822118 | *** | 1.33E-95 | *** |
| L LGN | total_free_recall | 1.64E-06 | 2.77E-07 | 5.90476 | 1.15E-08 | 250 | 34.86619 | 0.122395 | 0.139465 | 0.122395 | *** | 2.98E-07 | *** |
| L MDl | (Intercept) | 0.00017 | 4.41E-06 | 38.61258 | 2.40E-107 | 250 | 1490.931 | 0.856399 | 5.963726 | 0.856399 | *** | 5.21E-107 | *** |
| L MDl | total_free_recall | 1.05E-06 | 2.49E-07 | 4.219347 | 3.43E-05 | 250 | 17.80289 | 0.066478 | 0.071212 | 0.066478 | *** | 0.000111468 | *** |
| L PuA | (Intercept) | 0.000152 | 4.26E-06 | 35.78286 | 2.41E-100 | 250 | 1280.413 | 0.836645 | 5.121651 | 0.836645 | *** | 3.69E-100 | *** |
| L PuA | total_free_recall | 7.87E-07 | 2.41E-07 | 3.266679 | 0.001240572 | 250 | 10.67119 | 0.040937 | 0.042685 | 0.040937 | ** | 0.002056356 | ** |
| R VA | (Intercept) | 0.000258 | 5.50E-06 | 46.83429 | 9.29E-126 | 250 | 2193.451 | 0.897686 | 8.773804 | 0.897686 | *** | 1.21E-124 | *** |
| R VA | total_free_recall | 1.28E-06 | 3.11E-07 | 4.121787 | 5.12E-05 | 250 | 16.98913 | 0.063632 | 0.067957 | 0.063632 | *** | 0.000147838 | *** |
| R LGN | (Intercept) | 0.000163 | 4.94E-06 | 32.97145 | 5.23E-93 | 250 | 1087.116 | 0.81303 | 4.348465 | 0.81303 | *** | 6.47E-93 | *** |
| R LGN | total_free_recall | 1.42E-06 | 2.79E-07 | 5.072815 | 7.65E-07 | 250 | 25.73345 | 0.093327 | 0.102934 | 0.093327 | *** | 6.63E-06 | *** |
| L VA | (Intercept) | 0.000259 | 6.64E-06 | 39.07544 | 1.86E-108 | 250 | 1526.89 | 0.859305 | 6.10756 | 0.859305 | *** | 4.40E-108 | *** |
| L VA | total_free_recall | 1.23E-06 | 3.76E-07 | 3.260811 | 0.00126545 | 250 | 10.63289 | 0.040796 | 0.042532 | 0.040796 | ** | 0.002056356 | ** |
| R VPL | (Intercept) | 0.000591 | 1.10E-05 | 53.62381 | 3.96E-139 | 250 | 2875.513 | 0.920013 | 11.50205 | 0.920013 | *** | 1.03E-137 | *** |
| R VPL | total_free_recall | 8.19E-07 | 6.23E-07 | 1.313457 | 0.190233035 | 250 | 1.72517 | 0.006853 | 0.006901 | 0.006853 | ns | 0.190233035 | ns |
| R MDm | (Intercept) | 0.000516 | 1.18E-05 | 43.61759 | 7.14E-119 | 250 | 1902.494 | 0.883856 | 7.609977 | 0.883856 | *** | 3.71E-118 | *** |
| R MDm | total_free_recall | 2.83E-06 | 6.69E-07 | 4.226502 | 3.33E-05 | 250 | 17.86332 | 0.066688 | 0.071453 | 0.066688 | *** | 0.000111468 | *** |
| L MDm | (Intercept) | 0.000519 | 1.27E-05 | 40.91058 | 9.09E-113 | 250 | 1673.676 | 0.87004 | 6.694703 | 0.87004 | *** | 3.38E-112 | *** |
| L MDm | total_free_recall | 2.87E-06 | 7.17E-07 | 4.002694 | 8.26E-05 | 250 | 16.02156 | 0.060227 | 0.064086 | 0.060227 | *** | 0.000214713 | *** |
| R PuM | (Intercept) | 0.000798 | 1.77E-05 | 45.08676 | 4.58E-122 | 250 | 2032.816 | 0.890486 | 8.131265 | 0.890486 | *** | 3.97E-121 | *** |
| R PuM | total_free_recall | 4.47E-06 | 1.00E-06 | 4.457938 | 1.25E-05 | 250 | 19.87321 | 0.073639 | 0.079493 | 0.073639 | *** | 5.42E-05 | *** |
| L PuM | (Intercept) | 0.000799 | 2.01E-05 | 39.71993 | 5.49E-110 | 250 | 1577.673 | 0.863214 | 6.310692 | 0.863214 | *** | 1.43E-109 | *** |
| L PuM | total_free_recall | 3.65E-06 | 1.14E-06 | 3.204844 | 0.001527123 | 250 | 10.27102 | 0.039463 | 0.041084 | 0.039463 | ** | 0.002335601 | ** |
