# Supplementary material for: Structural MRI across lifespan reveals differential thalamic trajectories in Down syndrome
Source: Alzheimers Dement. 2026 Jul 14;22(7):e71671. doi: 10.1002/alz.71671 (PMC13369009; doi:10.1002/alz.71671)
Supplement: Supplementary file 8 — Supporting Information [file ALZ-22-e71671-s005.docx]

| **Term** | **b (SE)** | **t (df)** | **p** | **95% CI** |
| --- | --- | --- | --- | --- |
| **(Intercept)** | 0.005526 (0.000209) | 26.40 (485.00) |  | [0.005115, 0.005937] |
| **visit_age** | -0.00002082 (0.00000467) | -4.46 (485.00) |  | [-0.00002999, -0.00001165] |
| **hemisphereRight** | 0.0001199 (0.0002911) | 0.41 (485.00) | 0.681 | [-0.0004521, 0.0006919] |
| **apoe41** | 0.0007379 (0.0004349) | 1.70 (485.00) | 0.090 | [-0.0001167, 0.001592] |
| **apoe42** | 0.0005156 (0.001521) | 0.34 (485.00) | 0.735 | [-0.002473, 0.003504] |
| **sex2** | 0.0002584 (0.00005485) | 4.71 (485.00) | 3.23e-06 | [0.0001506, 0.0003662] |
| **visit_age:hemisphereRight** | -0.000001946 (0.000006589) | -0.30 (485.00) | 0.768 | [-0.00001489, 0.00001100] |
| **visit_age:apoe41** | -0.00001798 (0.000009776) | -1.84 (485.00) | 0.066 | [-0.00003719, 0.000001225] |
| **visit_age:apoe42** | -0.00001164 (0.00003948) | -0.30 (485.00) | 0.768 | [-0.00008920, 0.00006593] |
| **hemisphereRight:apoe41** | -0.0001979 (0.0006137) | -0.32 (485.00) | 0.747 | [-0.001404, 0.001008] |
| **hemisphereRight:apoe42** | -0.001284 (0.002149) | -0.60 (485.00) | 0.551 | [-0.005507, 0.002940] |
| **visit_age:hemisphereRight:apoe41** | 0.000004493 (0.00001380) | 0.33 (485.00) | 0.745 | [-0.00002262, 0.00003161] |
| **visit_age:hemisphereRight:apoe42** | 0.00002743 (0.00005579) | 0.49 (485.00) | 0.623 | [-0.00008220, |

Supplementary Table S6: APOE4 GLM statistics. Note: Coefficients are unstandardised (b) with standard error (SE). Df: 497 Total (Null); 485 Residual. Null deviance: 0.000219. Residual deviance: 0.0001757. AIC: -5958.
